# Supplementary material for: Ischemic ECG Pattern Recognition to Facilitate Interpretation While Task Switching: A Parallel Curriculum
Source: MedEdPORTAL. 2021 Sep 7;17:11182. doi: 10.15766/mep_2374-8265.11182 (PMC8421424; doi:10.15766/mep_2374-8265.11182)
Supplement: Supplementary file 1 — Introduction Lecture.pptxKnowledge Pretest Answer Sheet.docxECG Handout.docxECG Handout Answers.docxReview Lecture.pptxPresurvey of Confidence.docxPostsurvey of Confidence.docxCourse Evaluation.docxDelayed Knowledge Posttest.docx [file mep_2374-8265.11182-s001.zip › A. Introduction Lecture.pptx]

## Slide 1
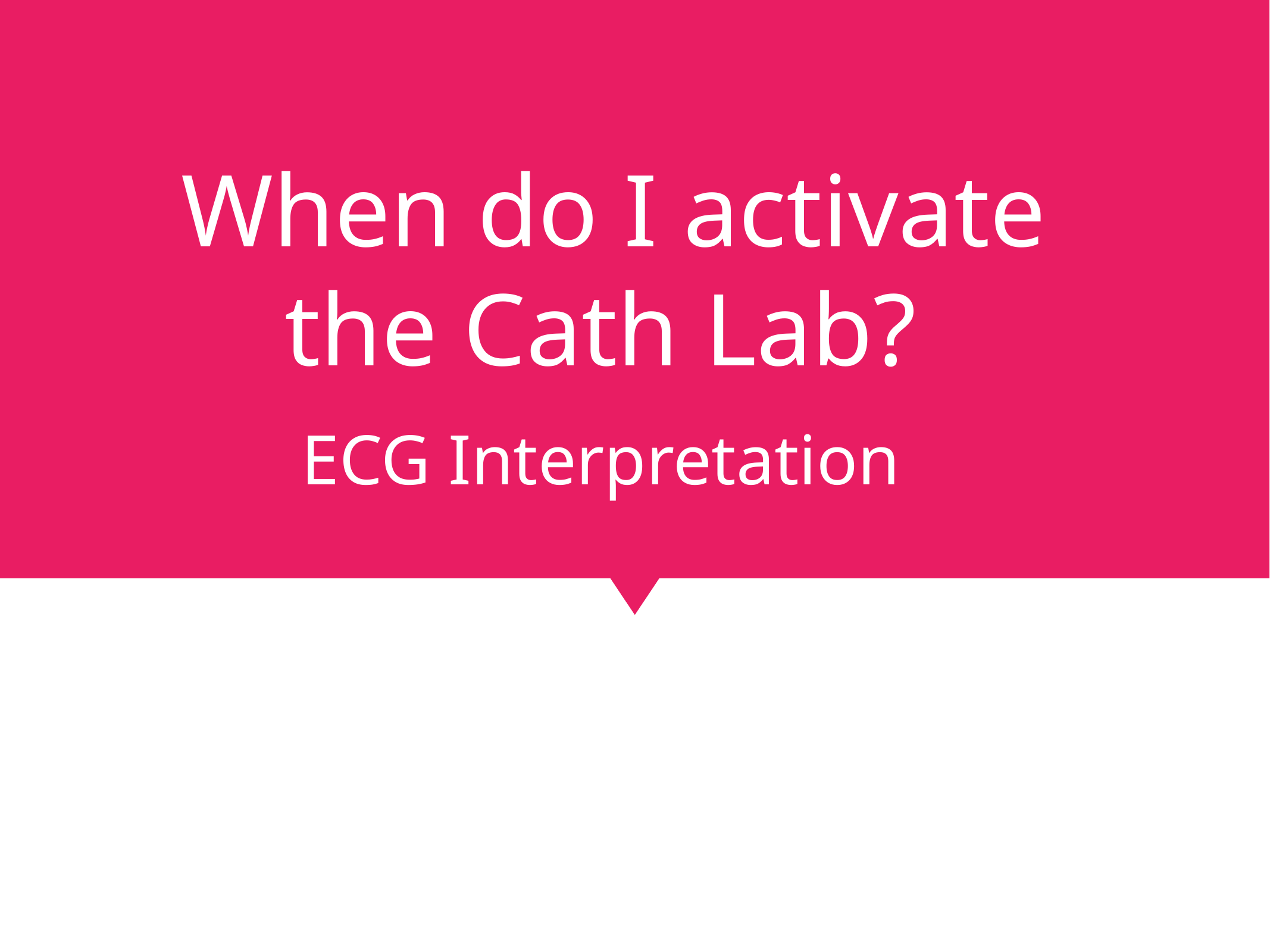

# When do I activate the Cath Lab?
ECG Interpretation

## Slide 2
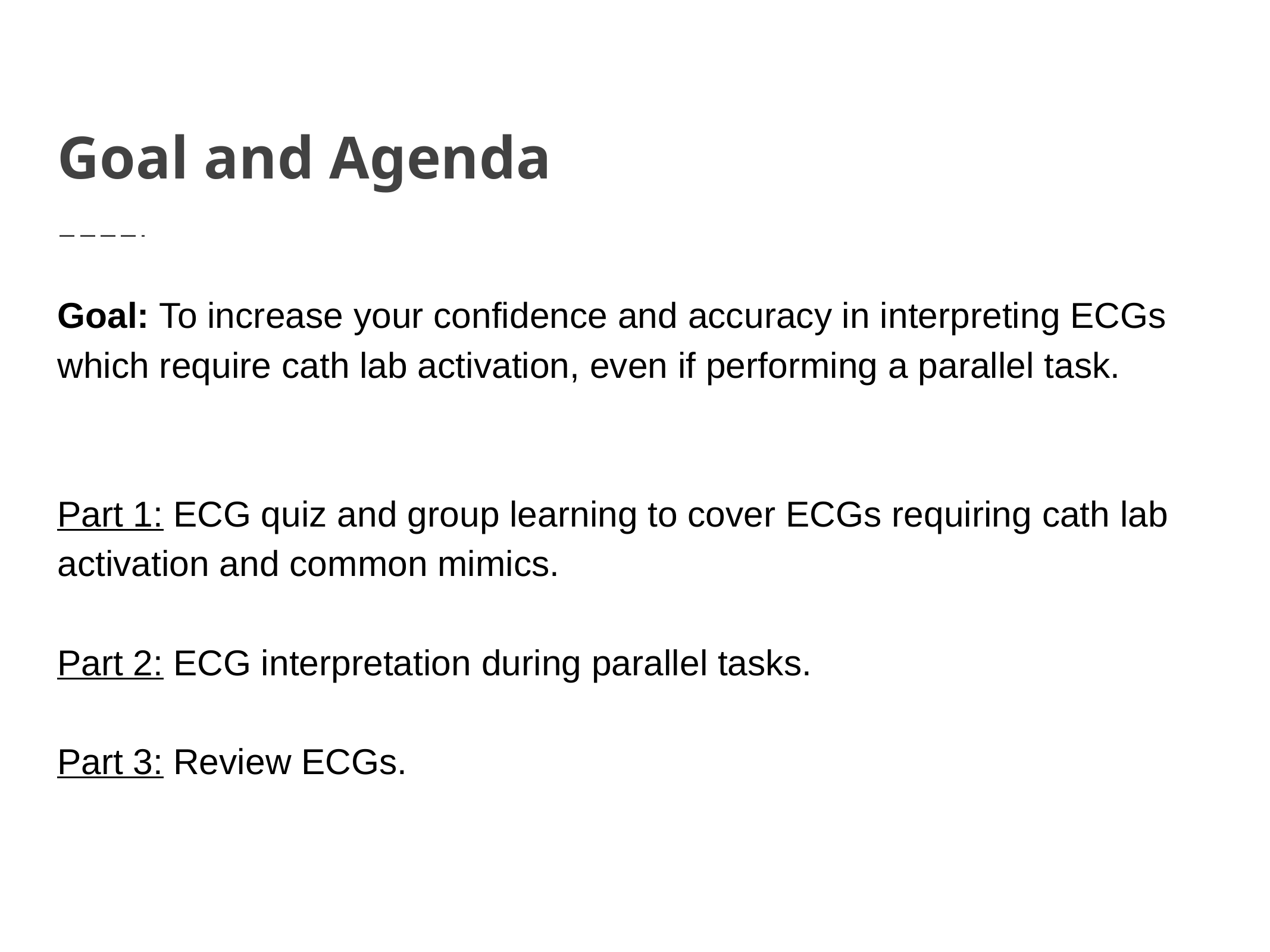

# Goal and Agenda
Goal: To increase your confidence and accuracy in interpreting ECGs which require cath lab activation, even if performing a parallel task.
Part 1: ECG quiz and group learning to cover ECGs requiring cath lab activation and common mimics.
Part 2: ECG interpretation during parallel tasks.
Part 3: Review ECGs.

## Slide 3
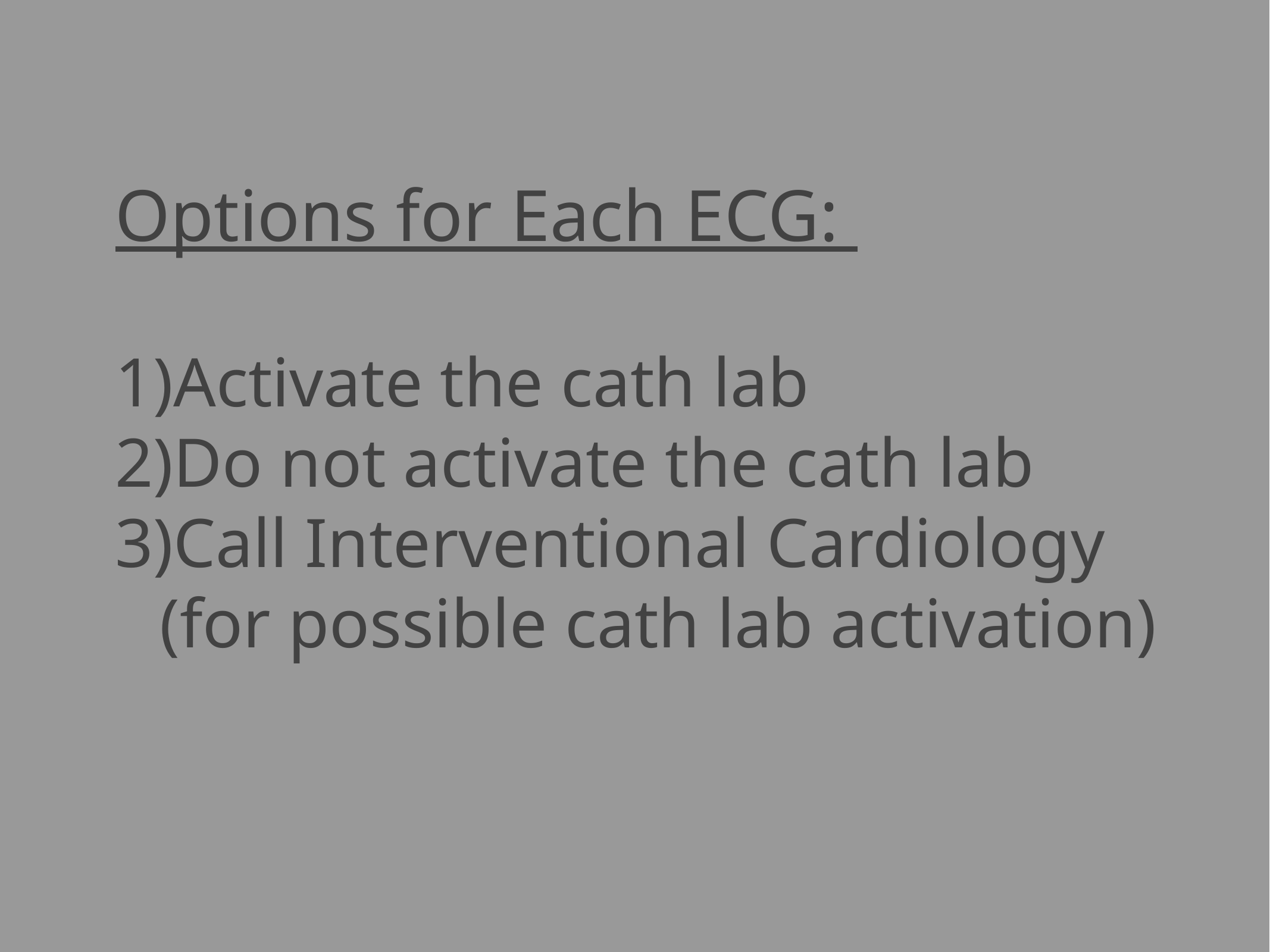

# Options for Each ECG:
Activate the cath lab
Do not activate the cath lab
Call Interventional Cardiology (for possible cath lab activation)

## Slide 4
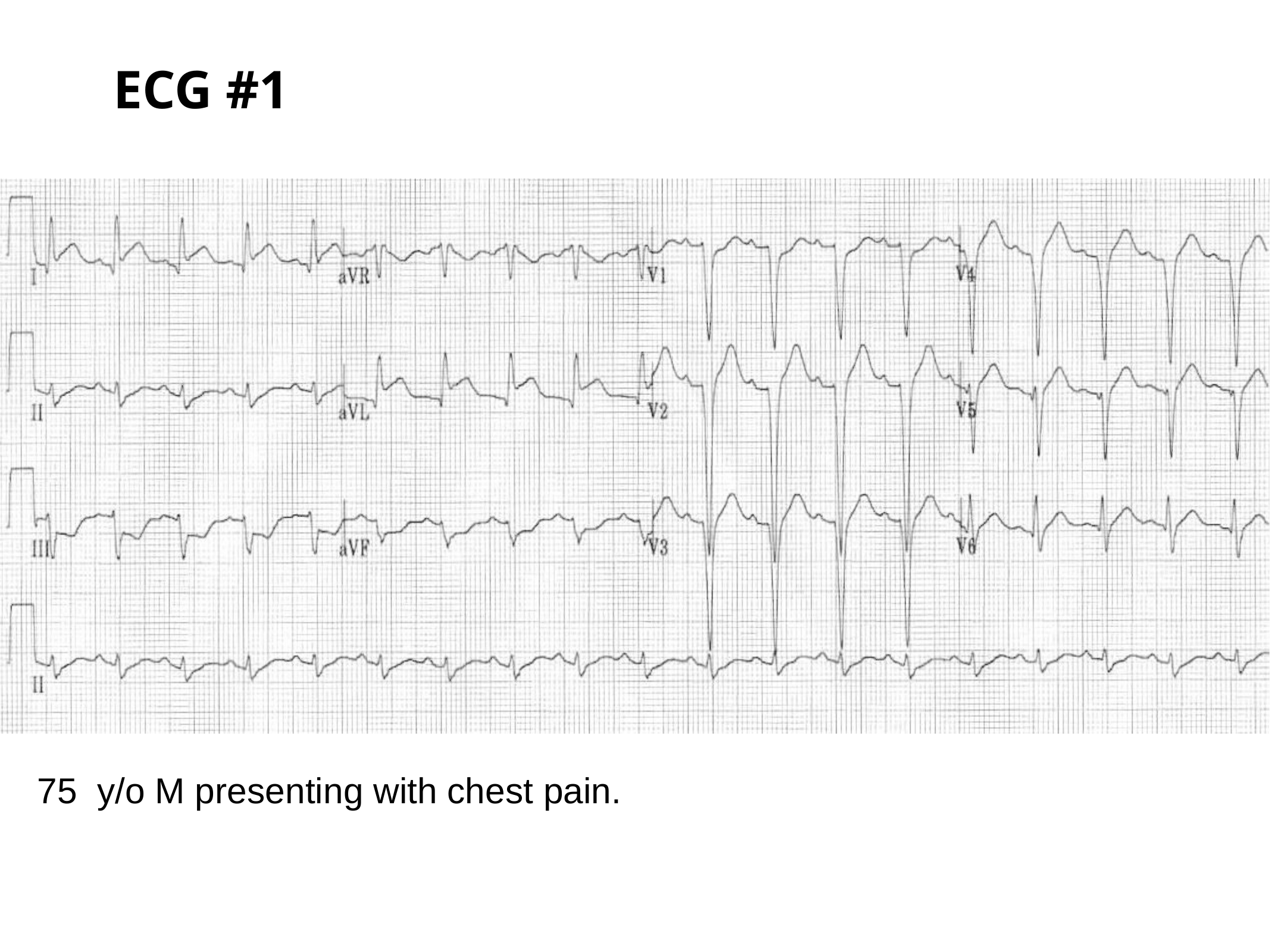

ECG #1
75 y/o M presenting with chest pain.

## Slide 5
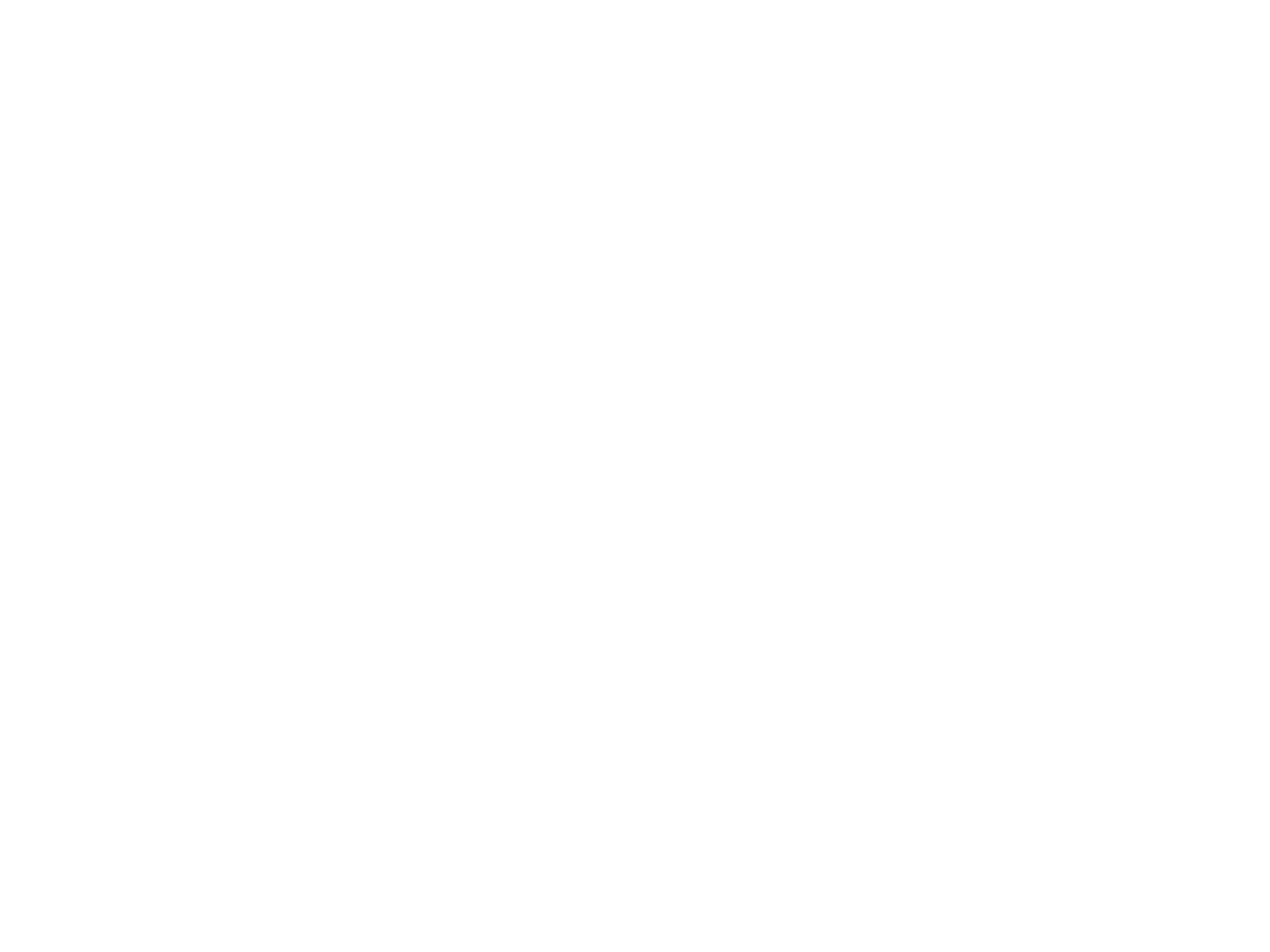

## Slide 6
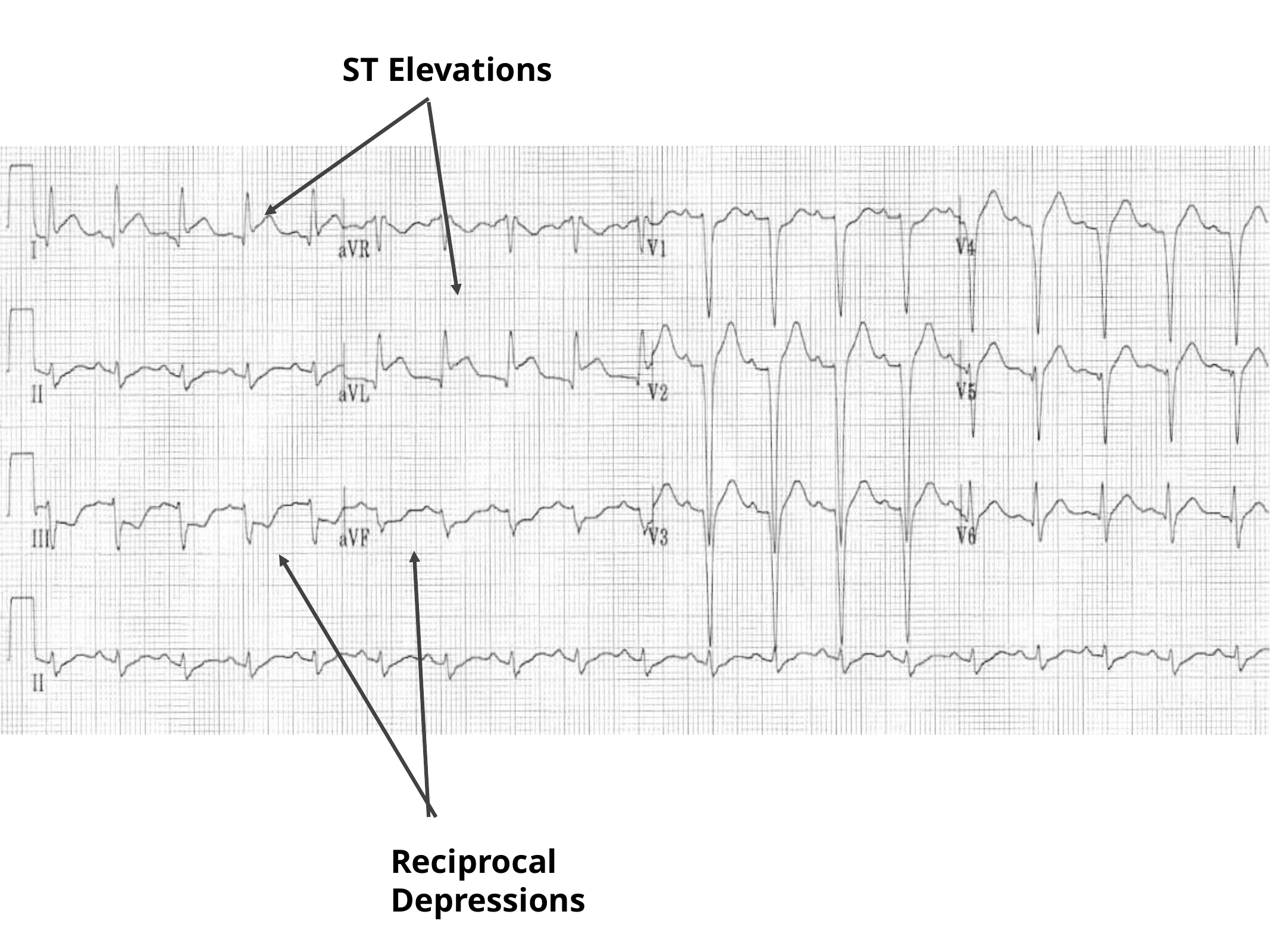

ST Elevations
Reciprocal Depressions

## Slide 7
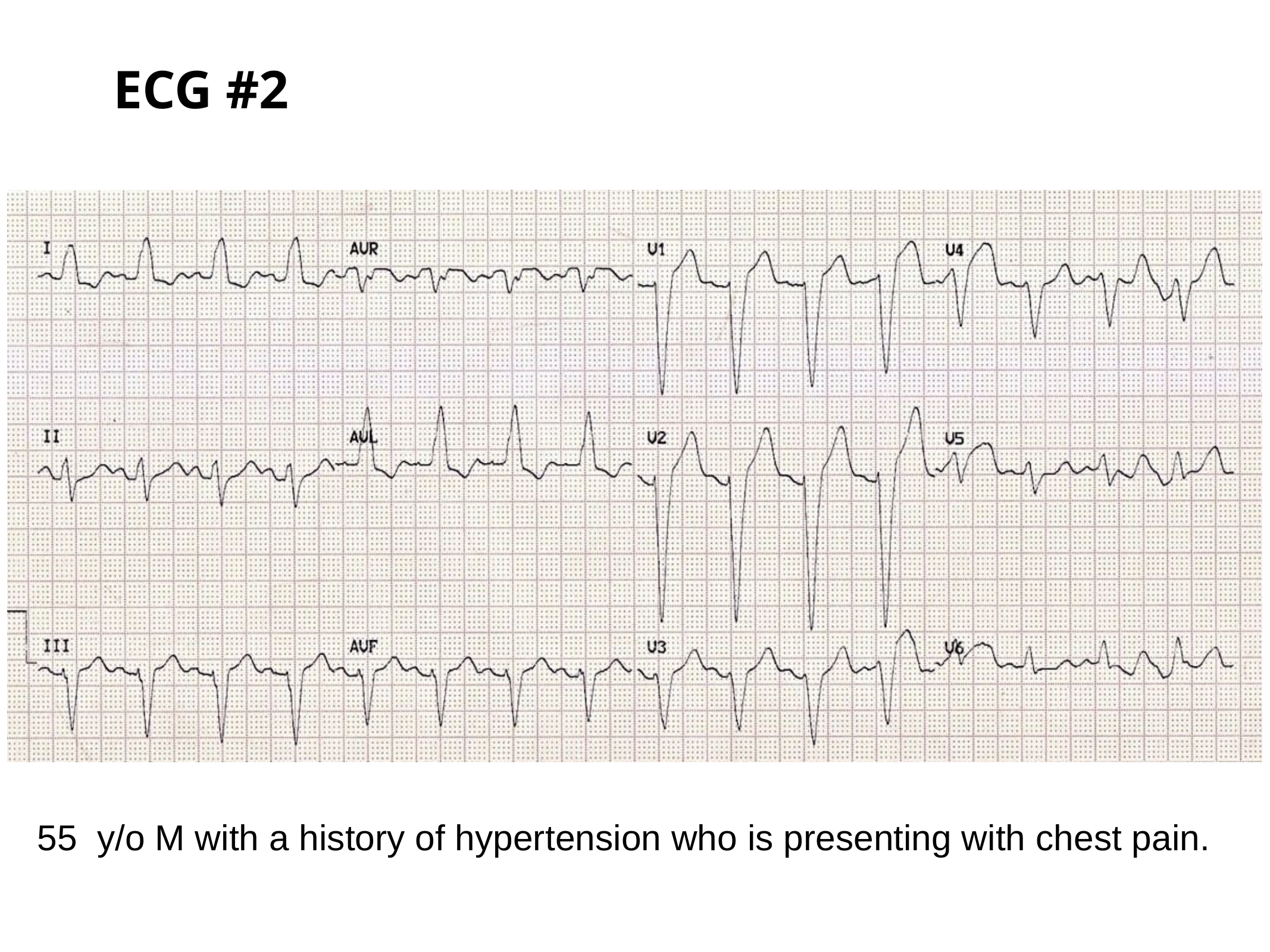

ECG #2
55 y/o M with a history of hypertension who is presenting with chest pain.

## Slide 8
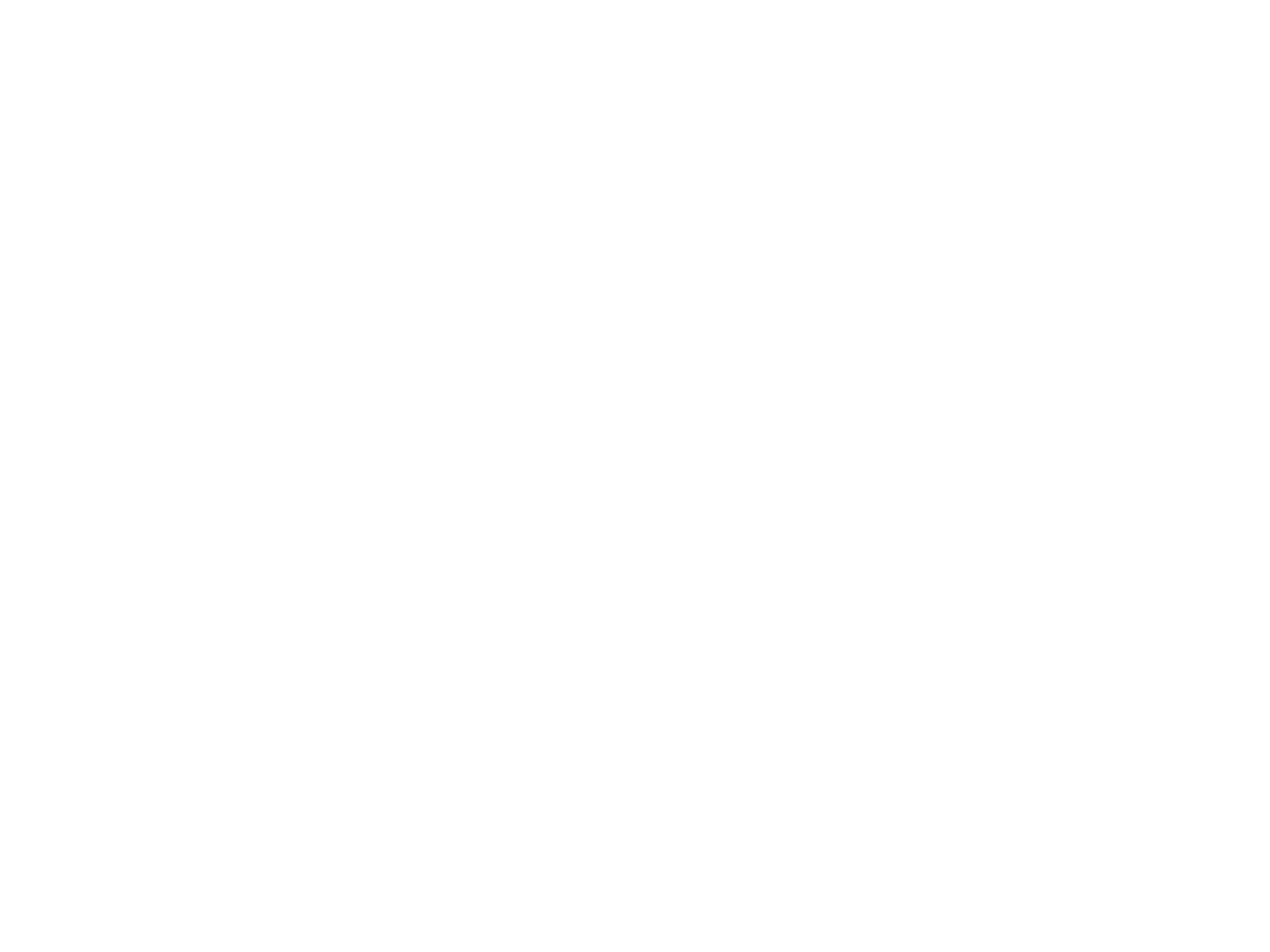

## Slide 9
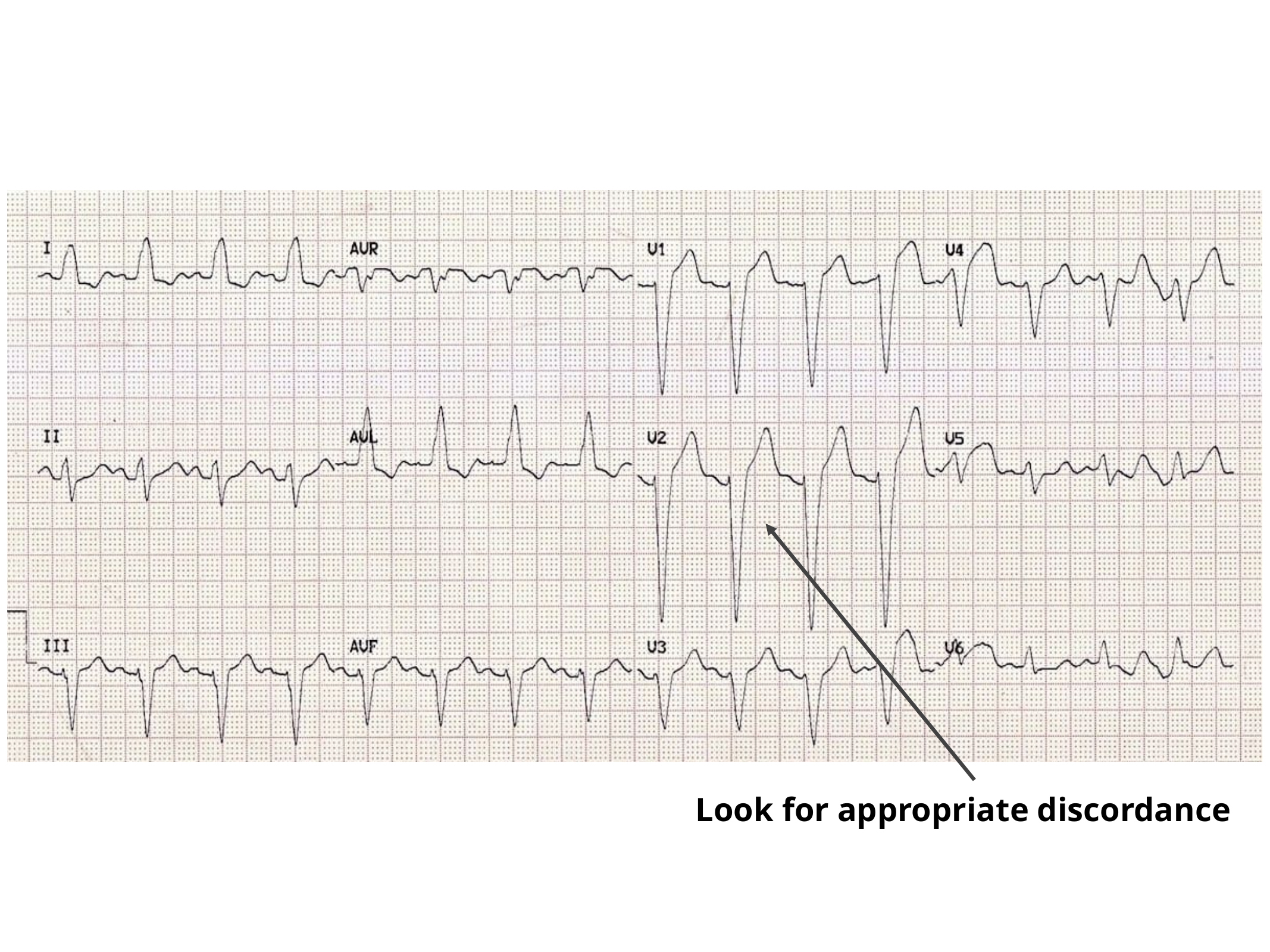

Look for appropriate discordance

## Slide 10
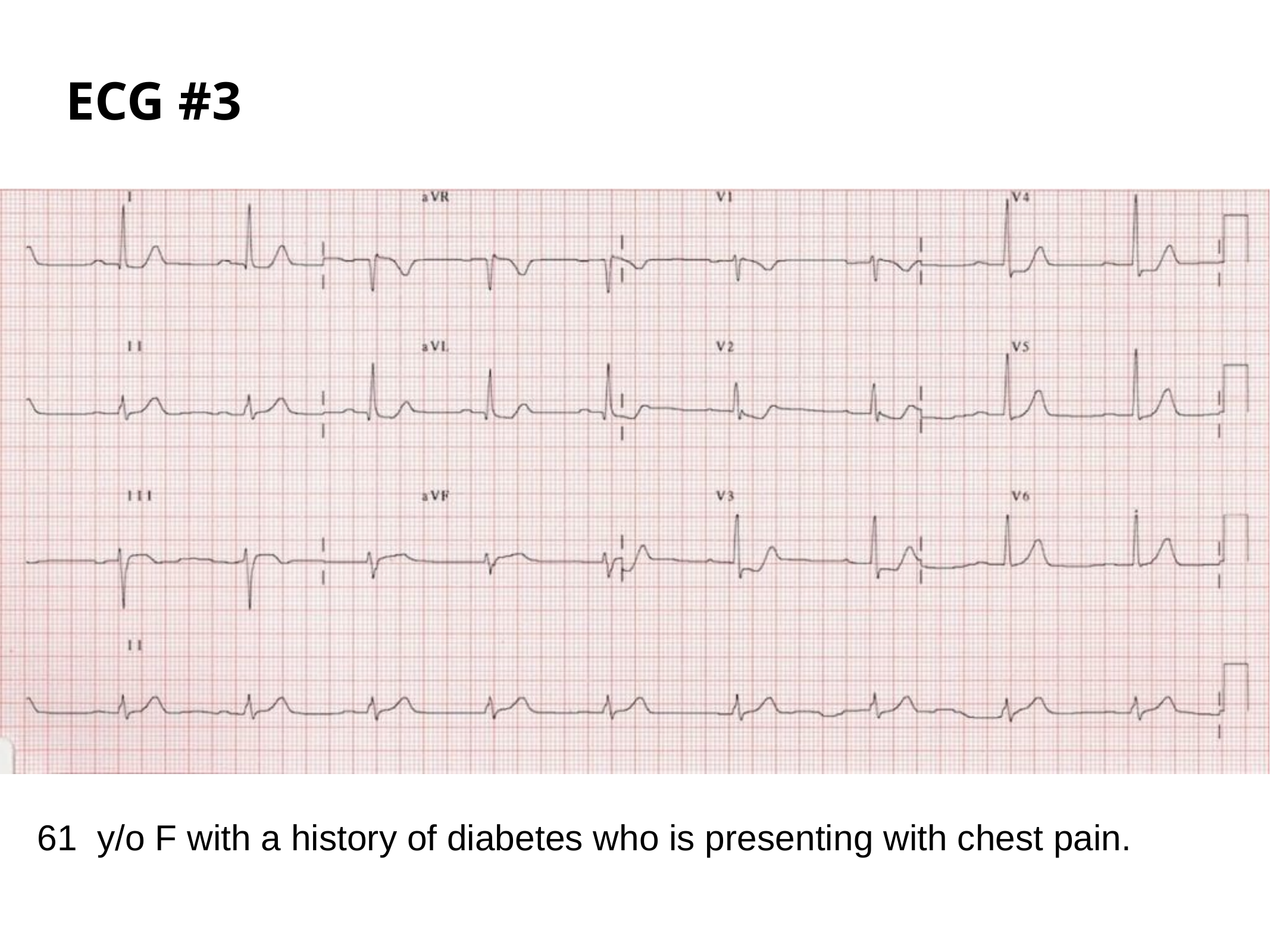

ECG #3
61 y/o F with a history of diabetes who is presenting with chest pain.

## Slide 11
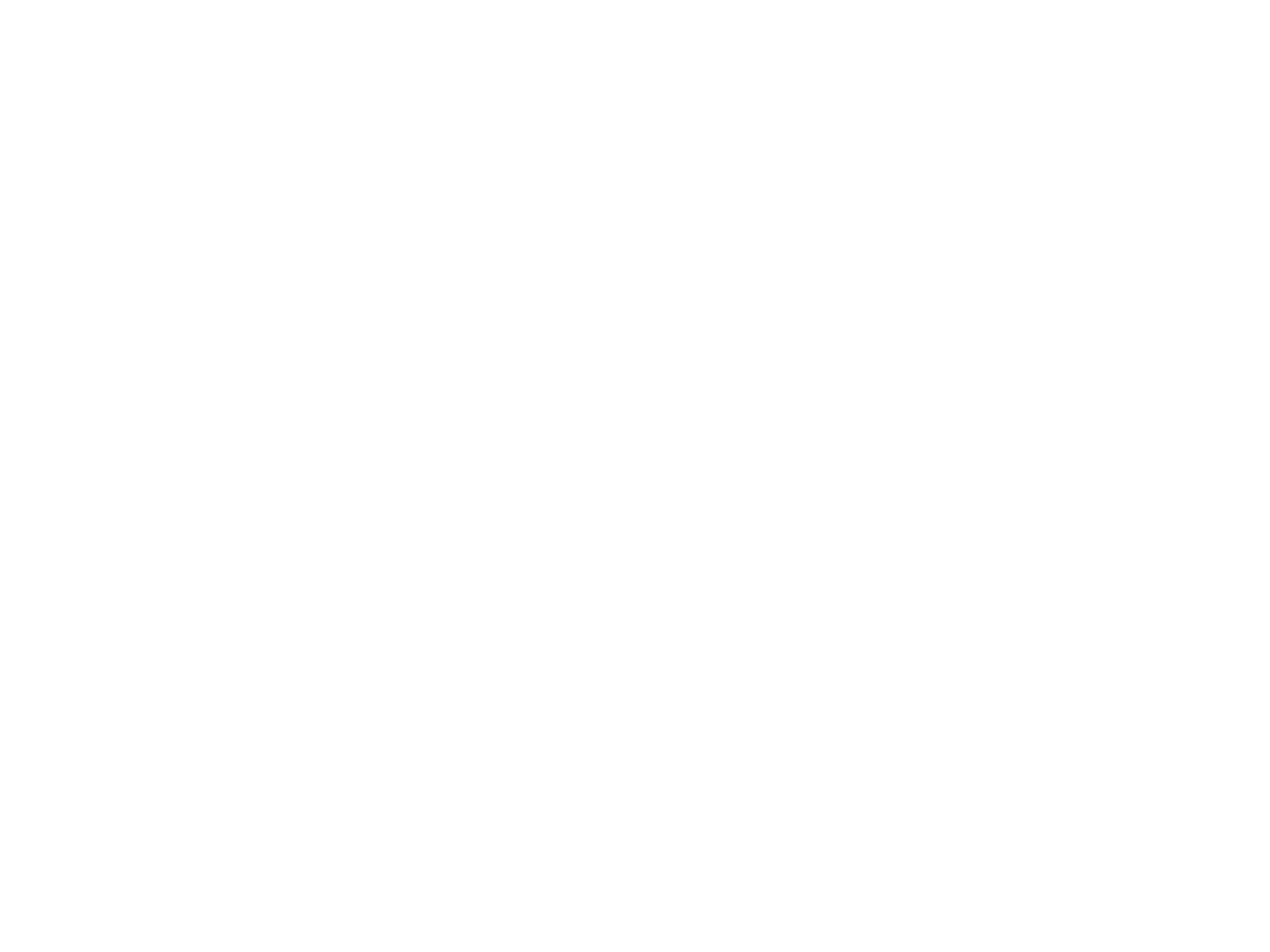

## Slide 12
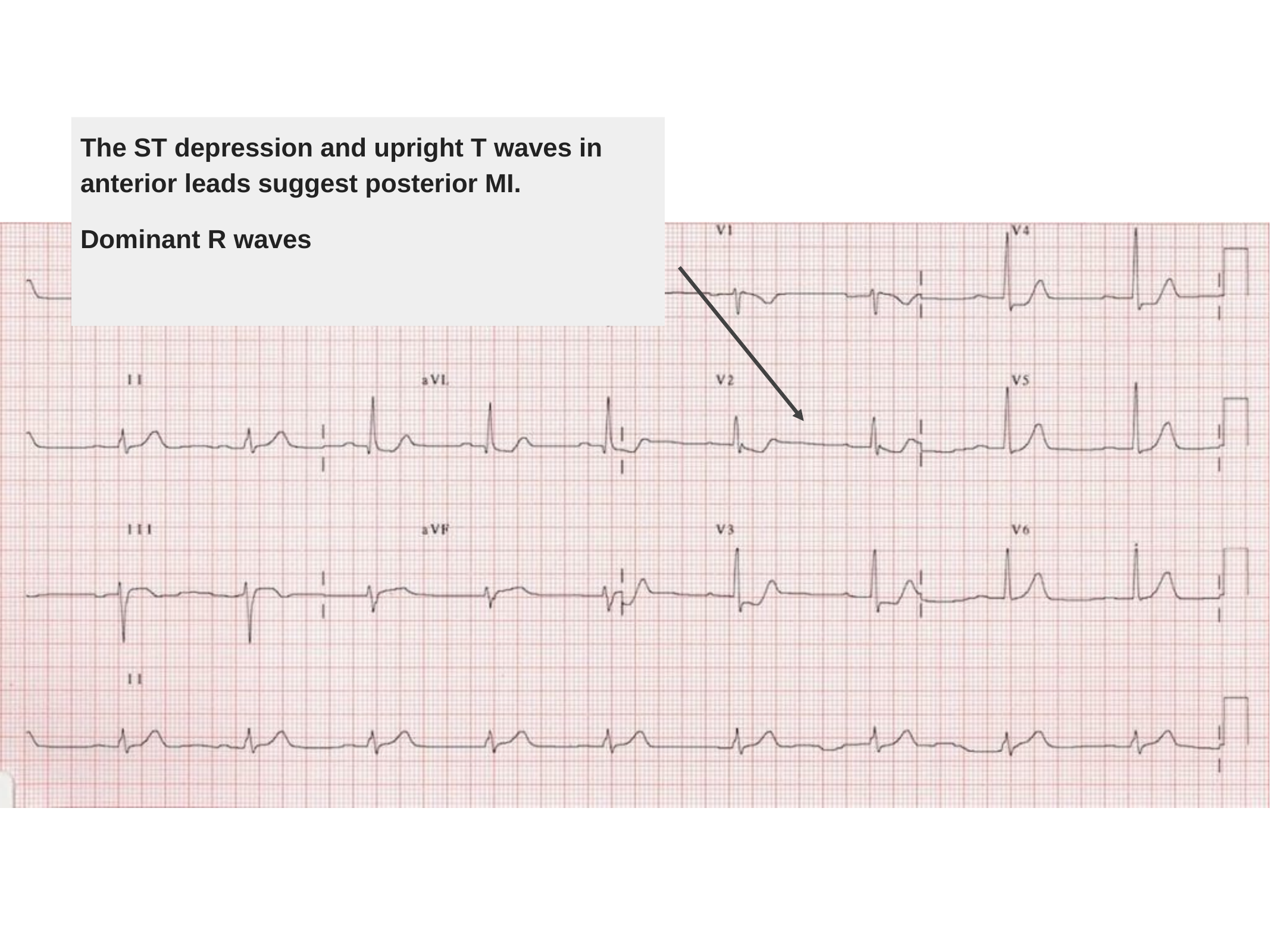

The ST depression and upright T waves in anterior leads suggest posterior MI.
Dominant R waves

## Slide 13
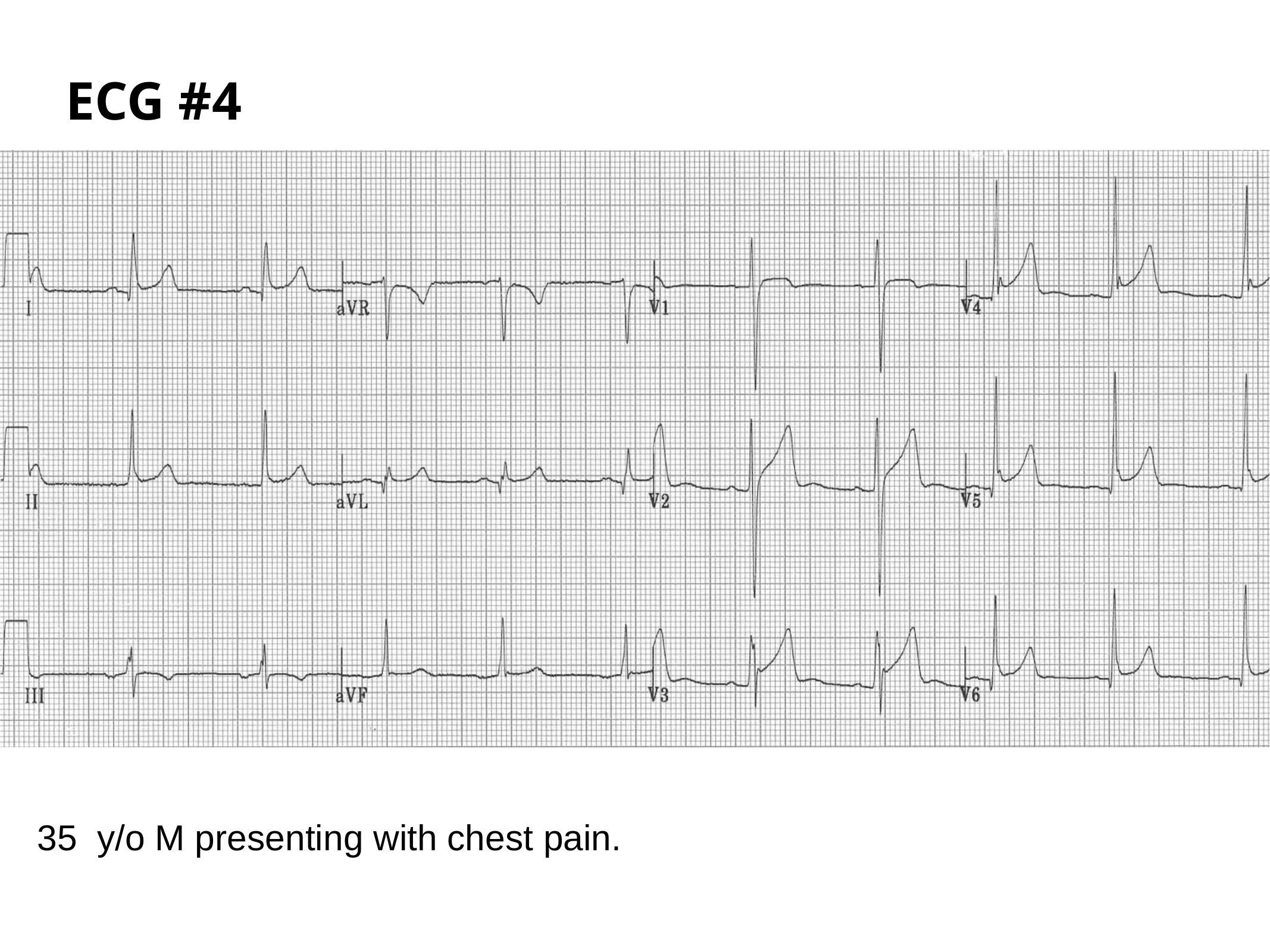

ECG #4
35 y/o M presenting with chest pain.

## Slide 14
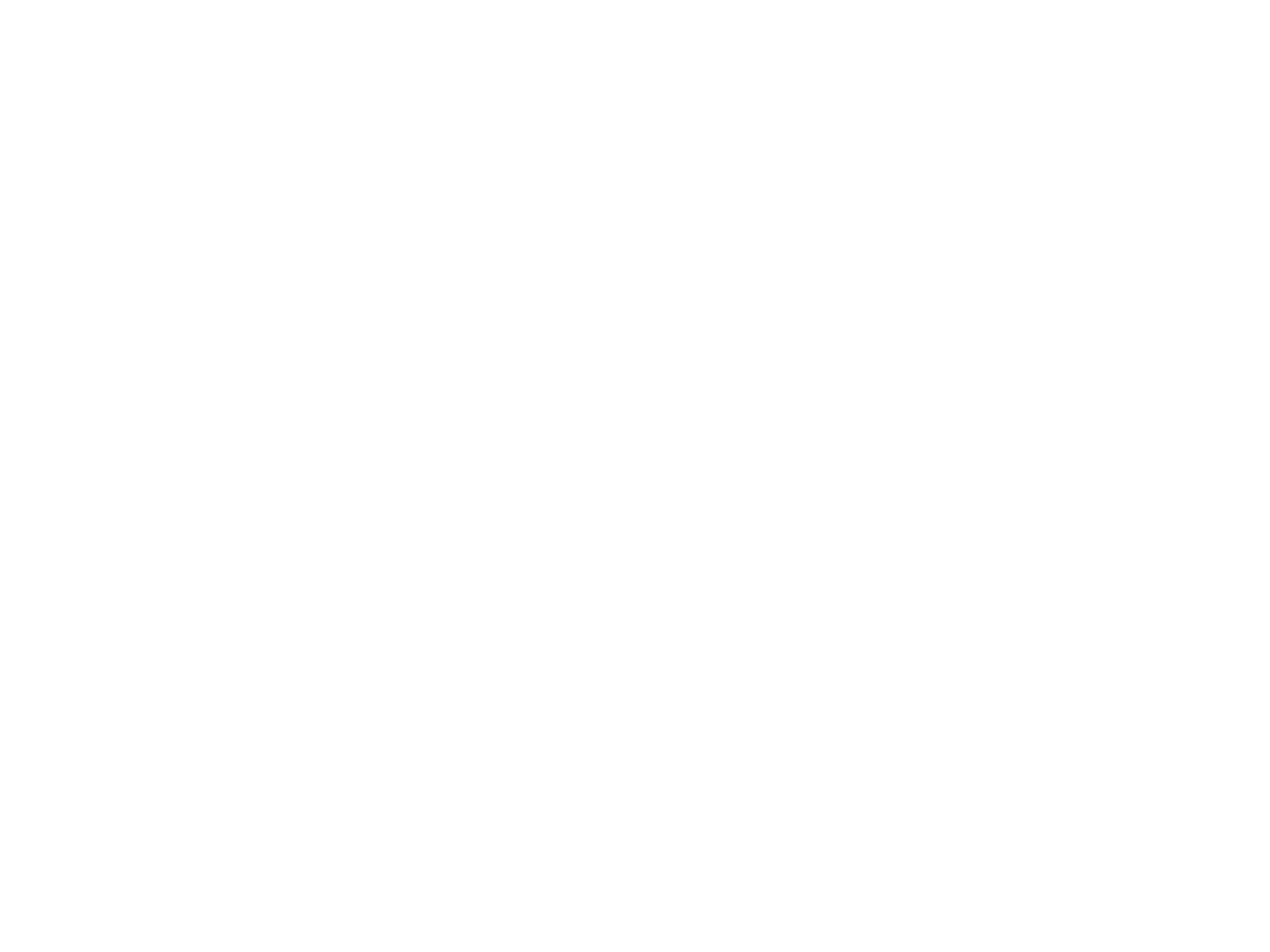

## Slide 15
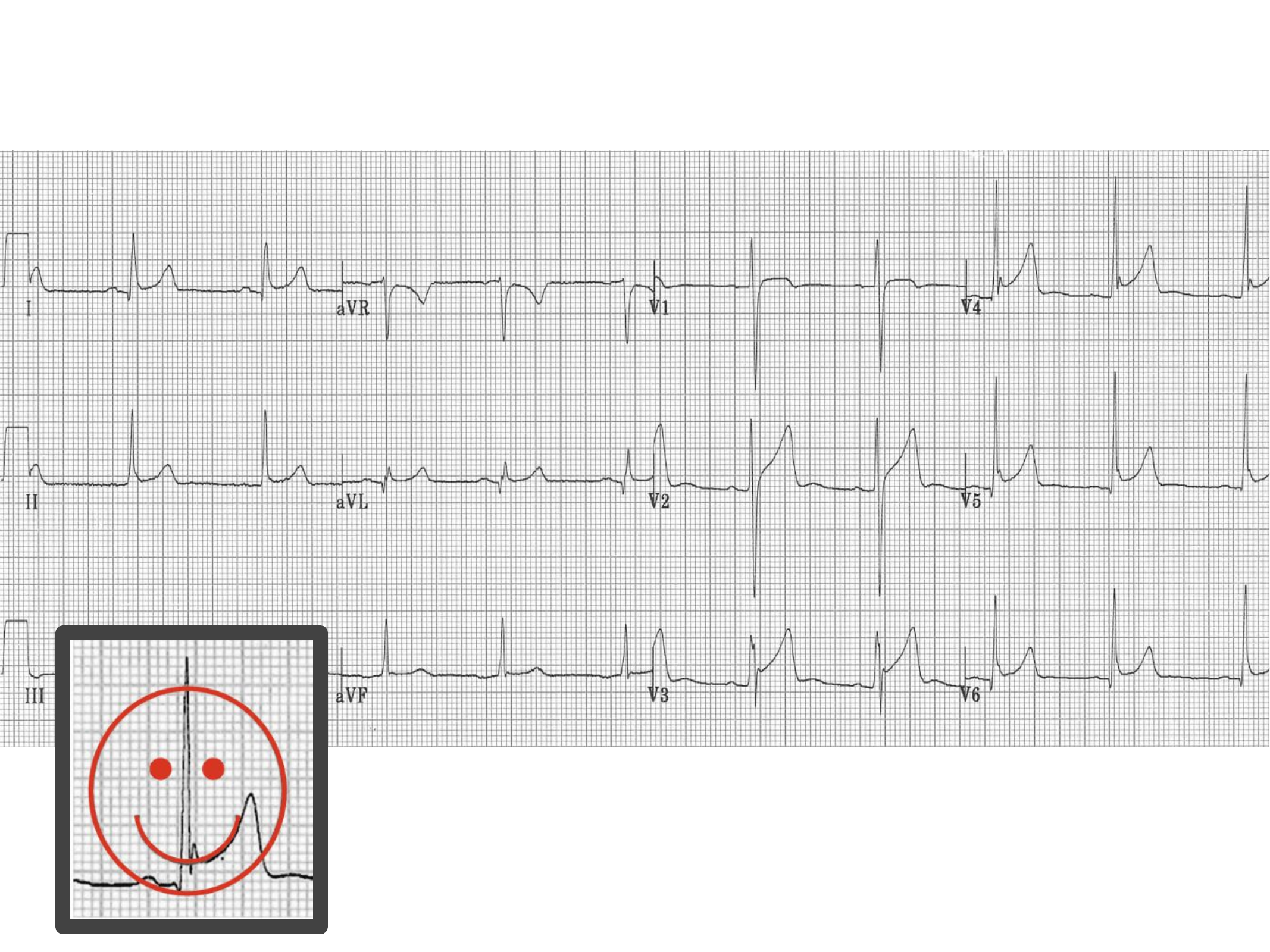

## Slide 16
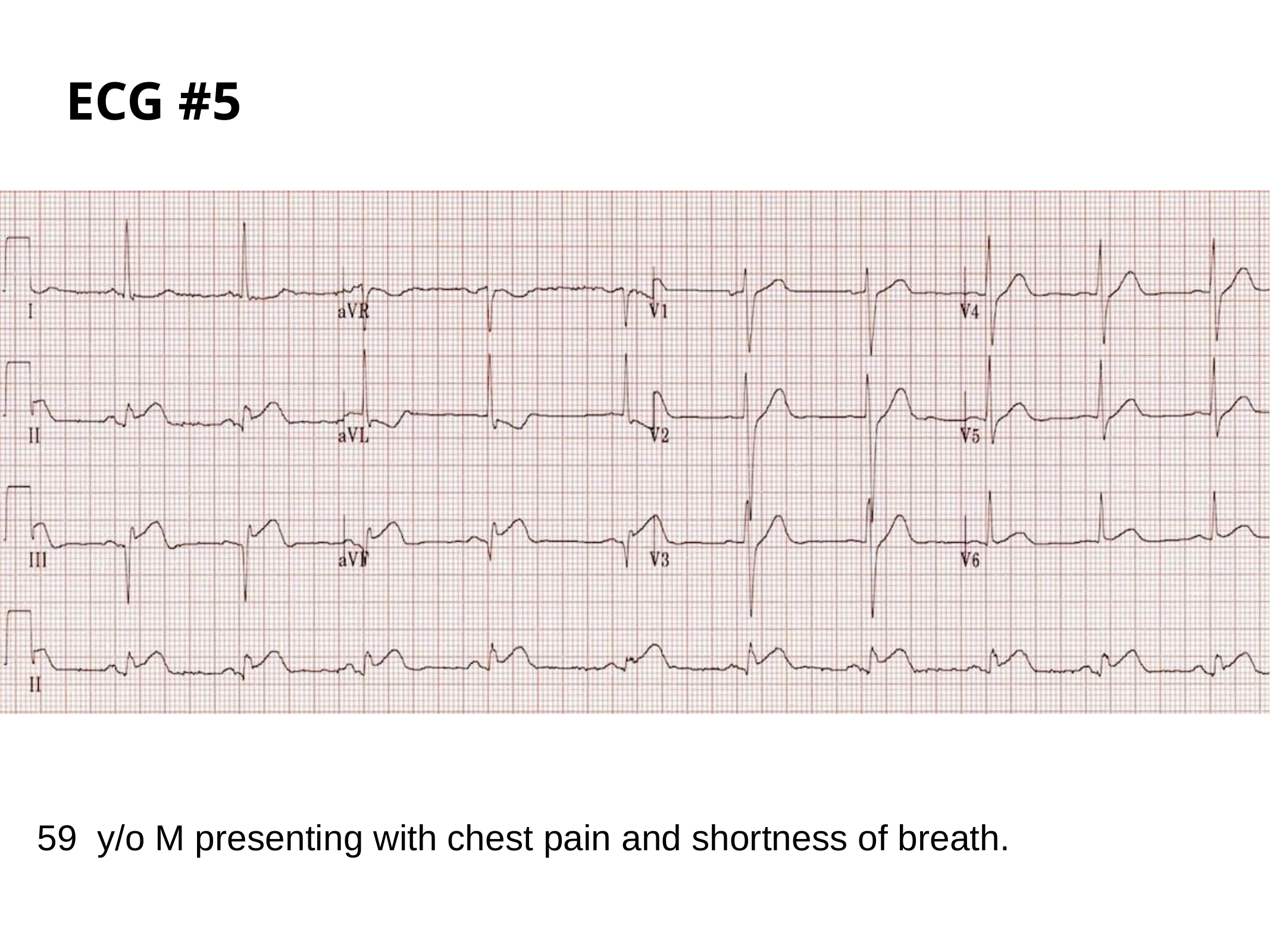

ECG #5
59 y/o M presenting with chest pain and shortness of breath.

## Slide 17
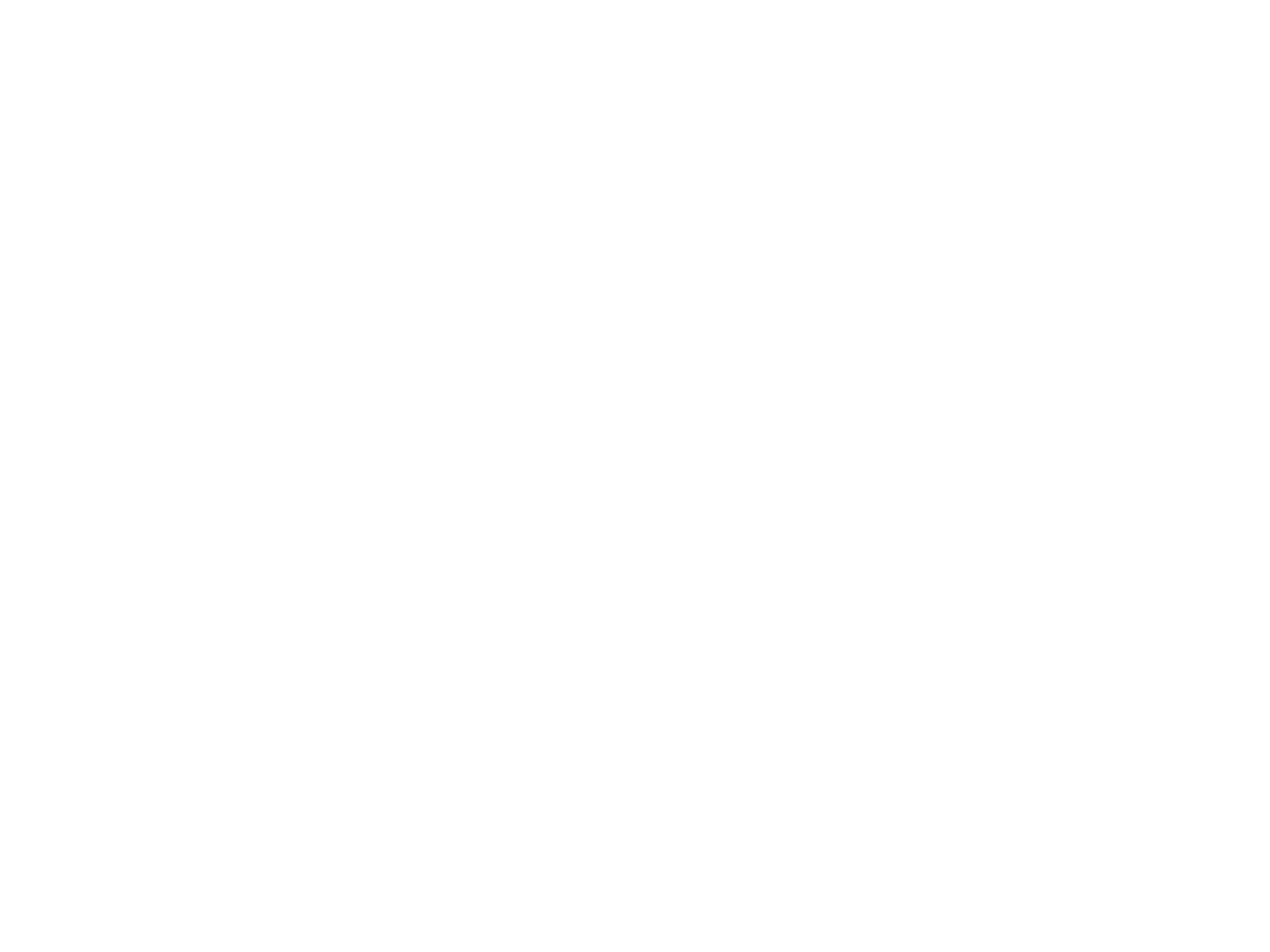

## Slide 18
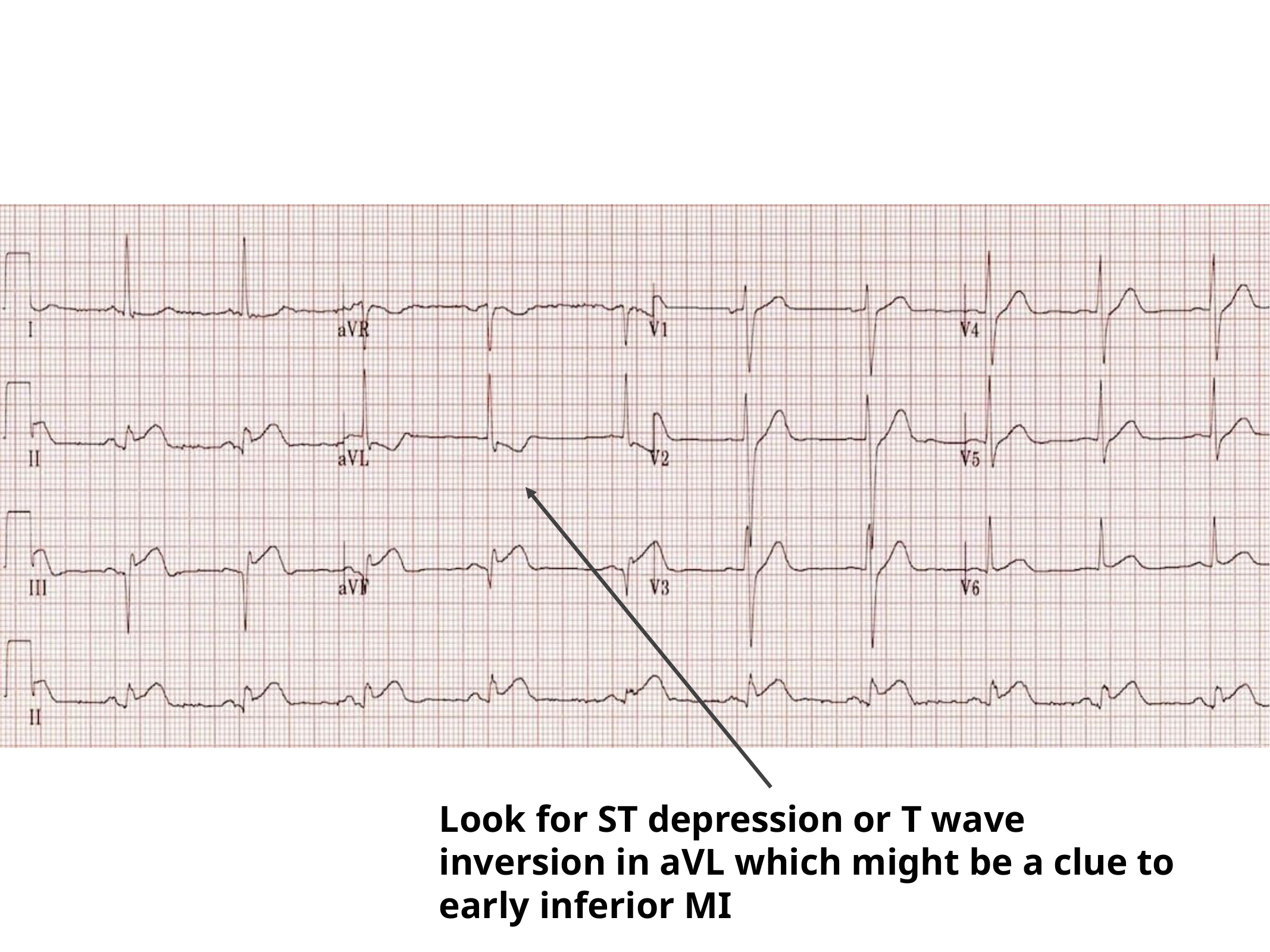

Look for ST depression or T wave inversion in aVL which might be a clue to early inferior MI

## Slide 19
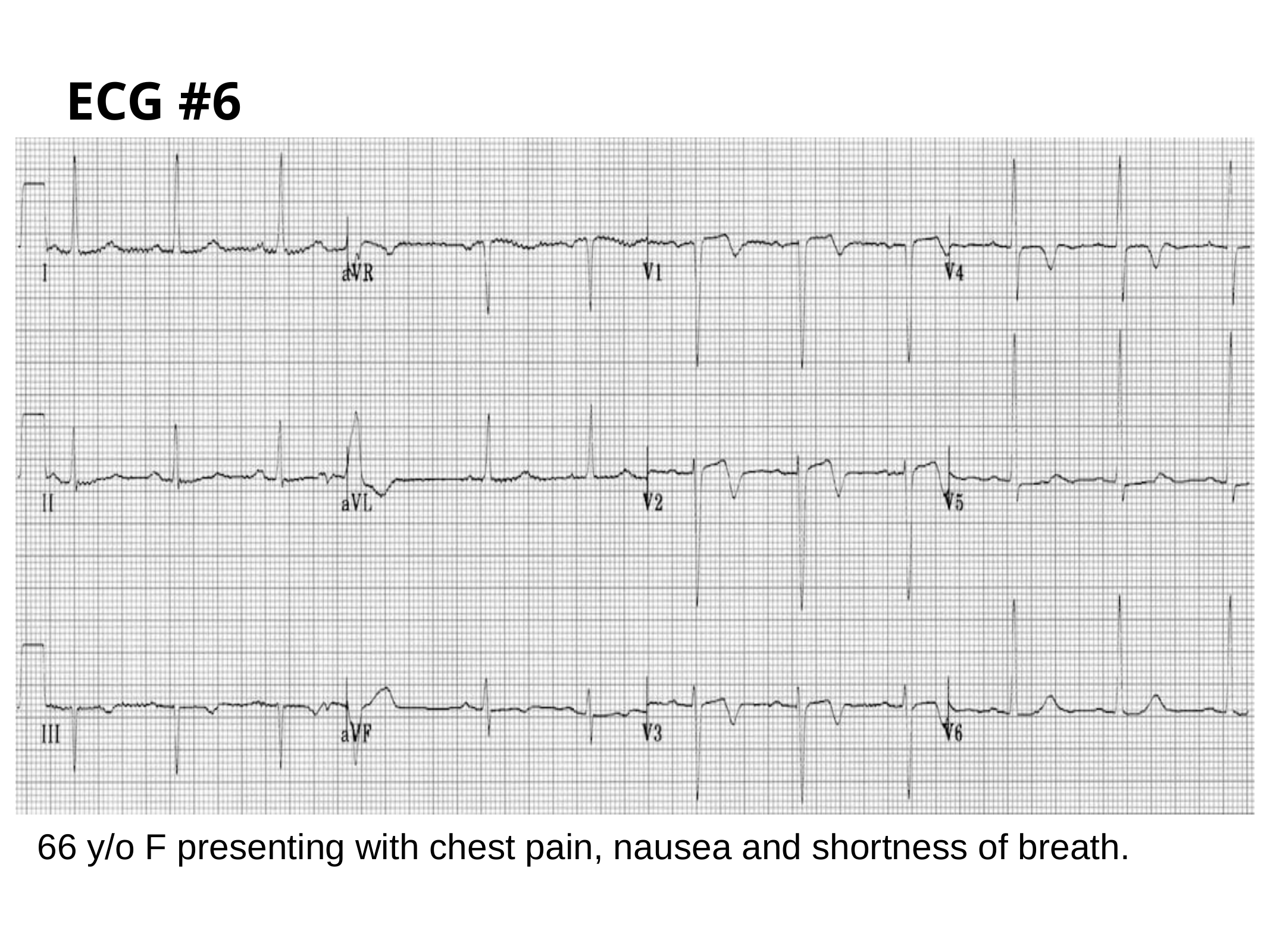

ECG #6
66 y/o F presenting with chest pain, nausea and shortness of breath.

## Slide 20
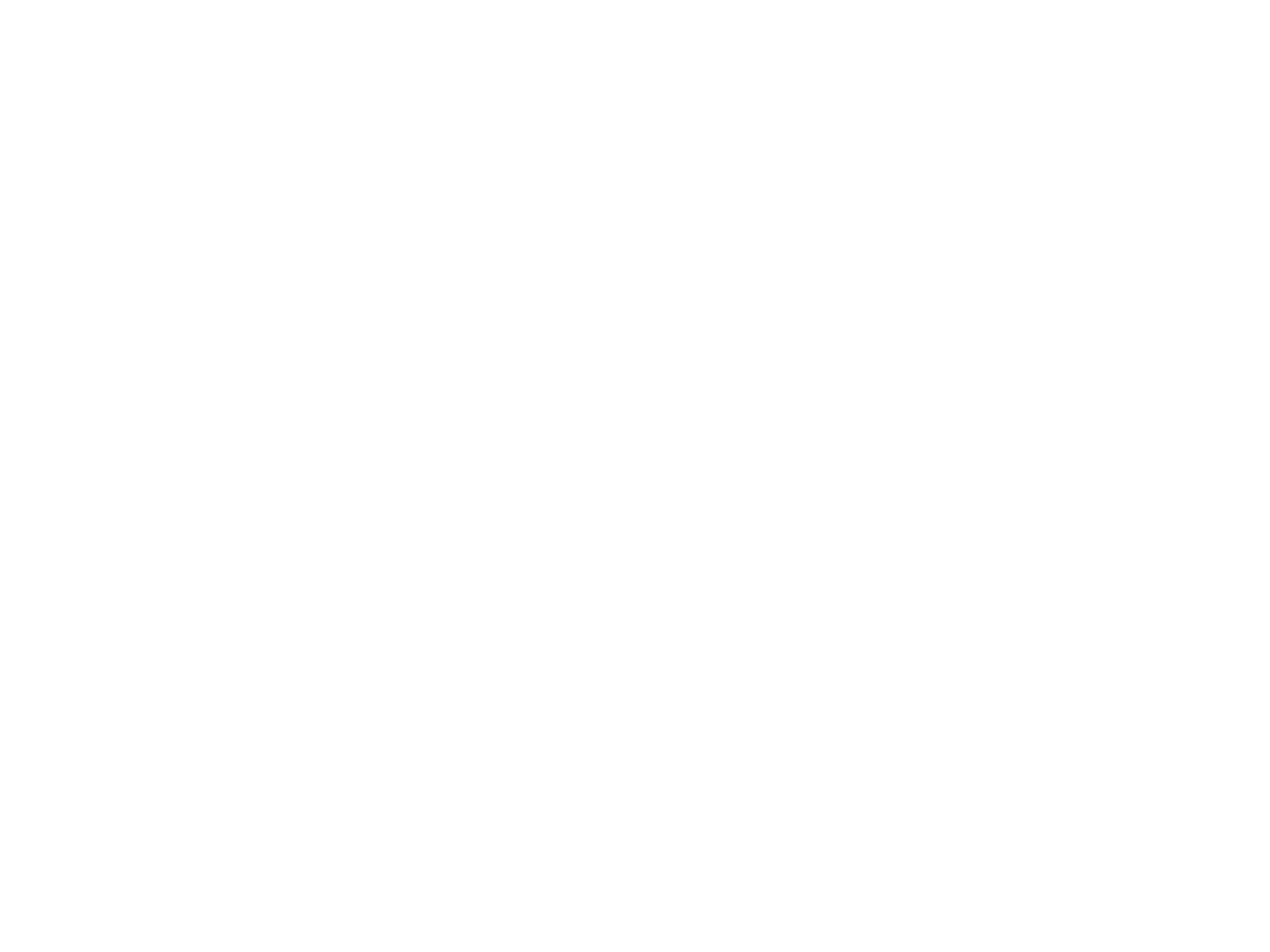

## Slide 21
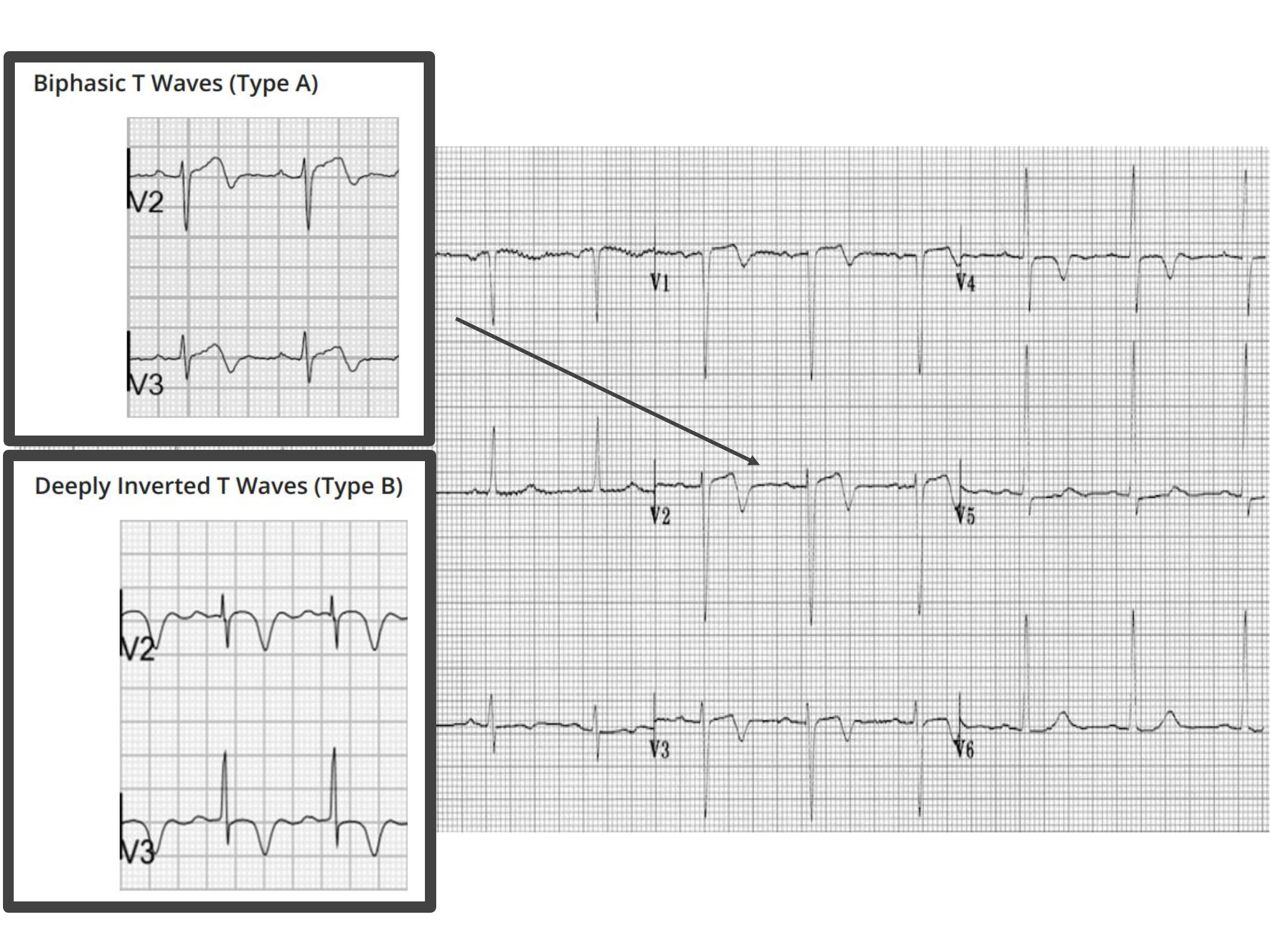

## Slide 22
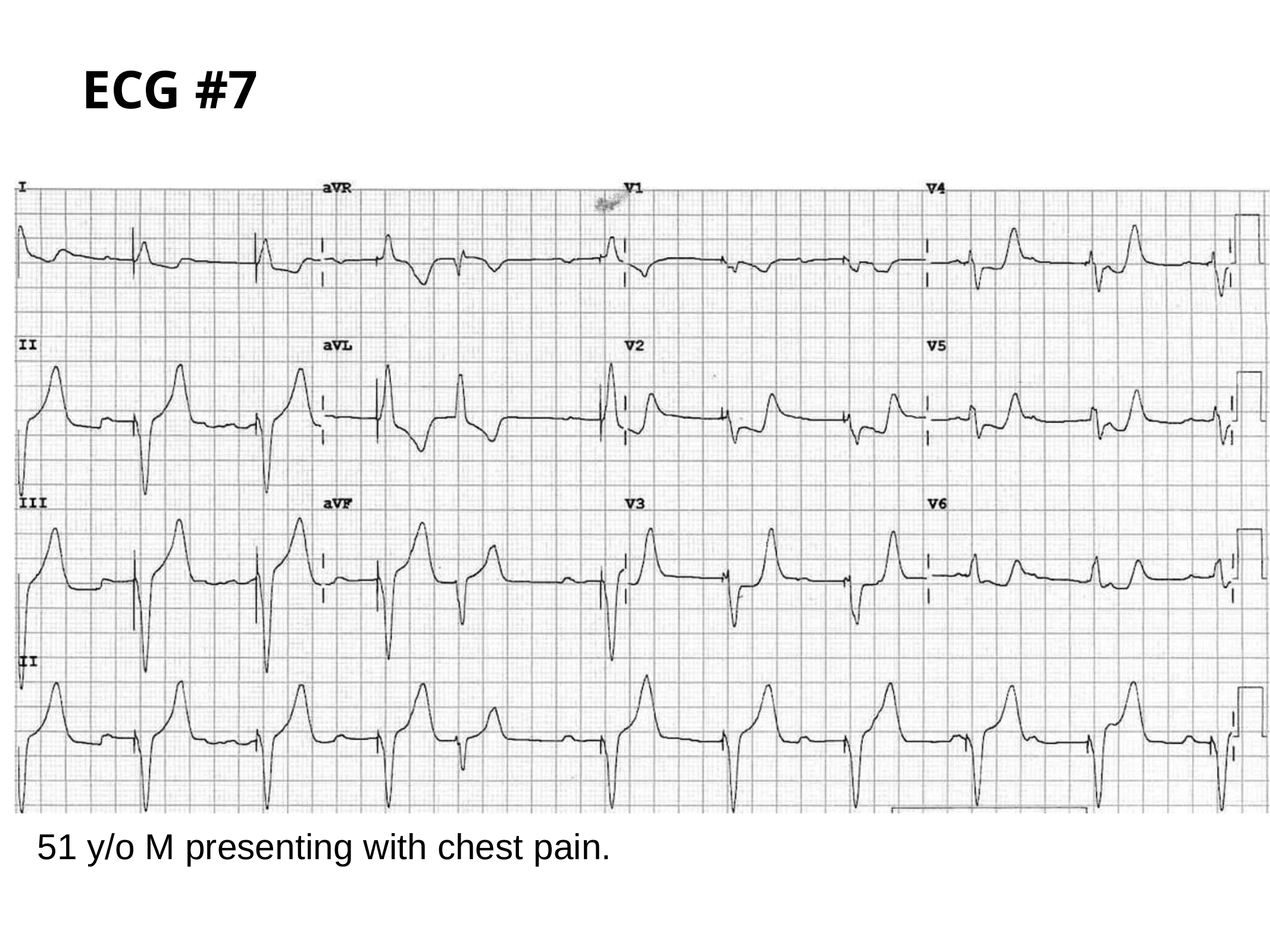

ECG #7
51 y/o M presenting with chest pain.

## Slide 23
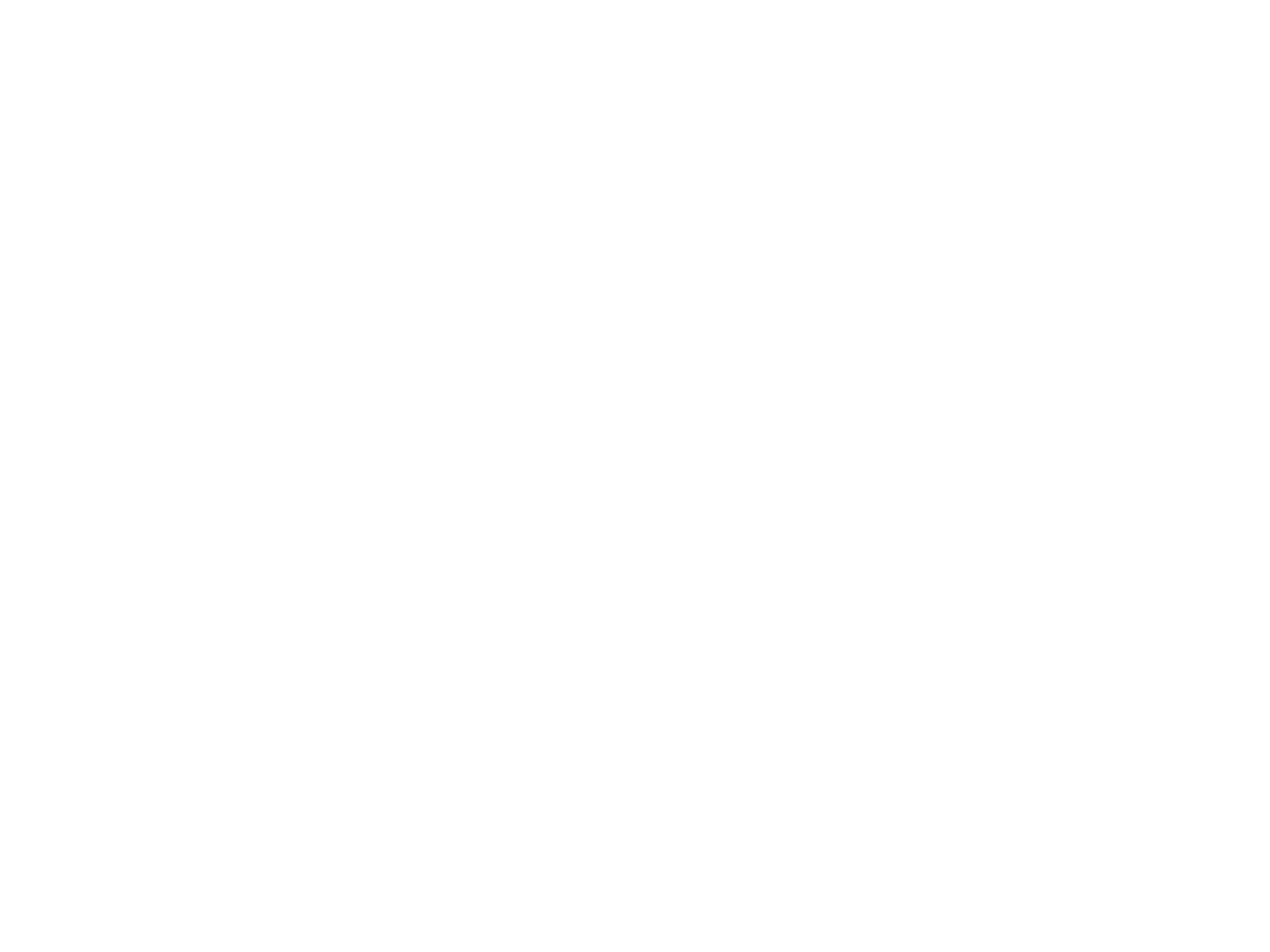

## Slide 24
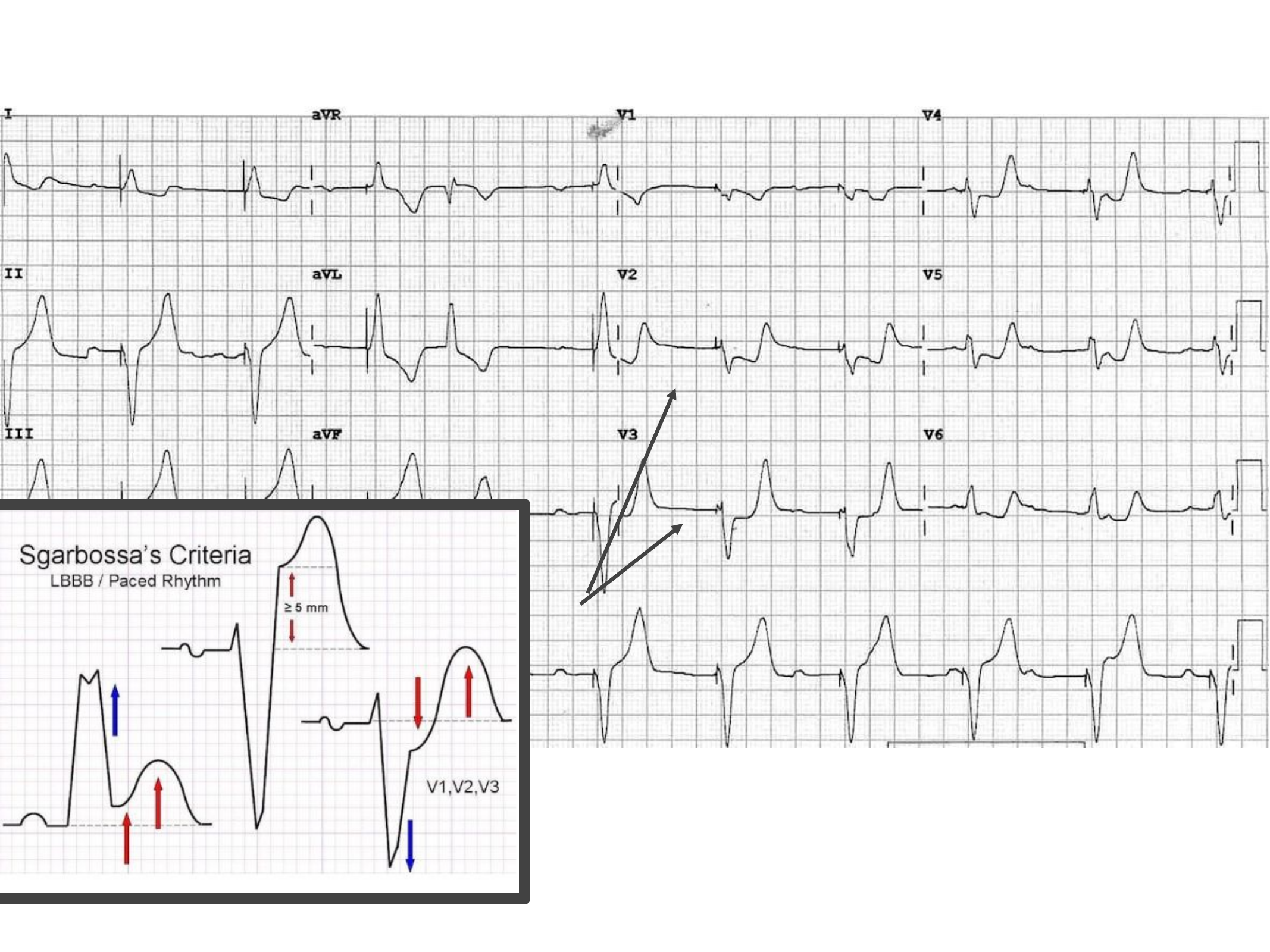

## Slide 25
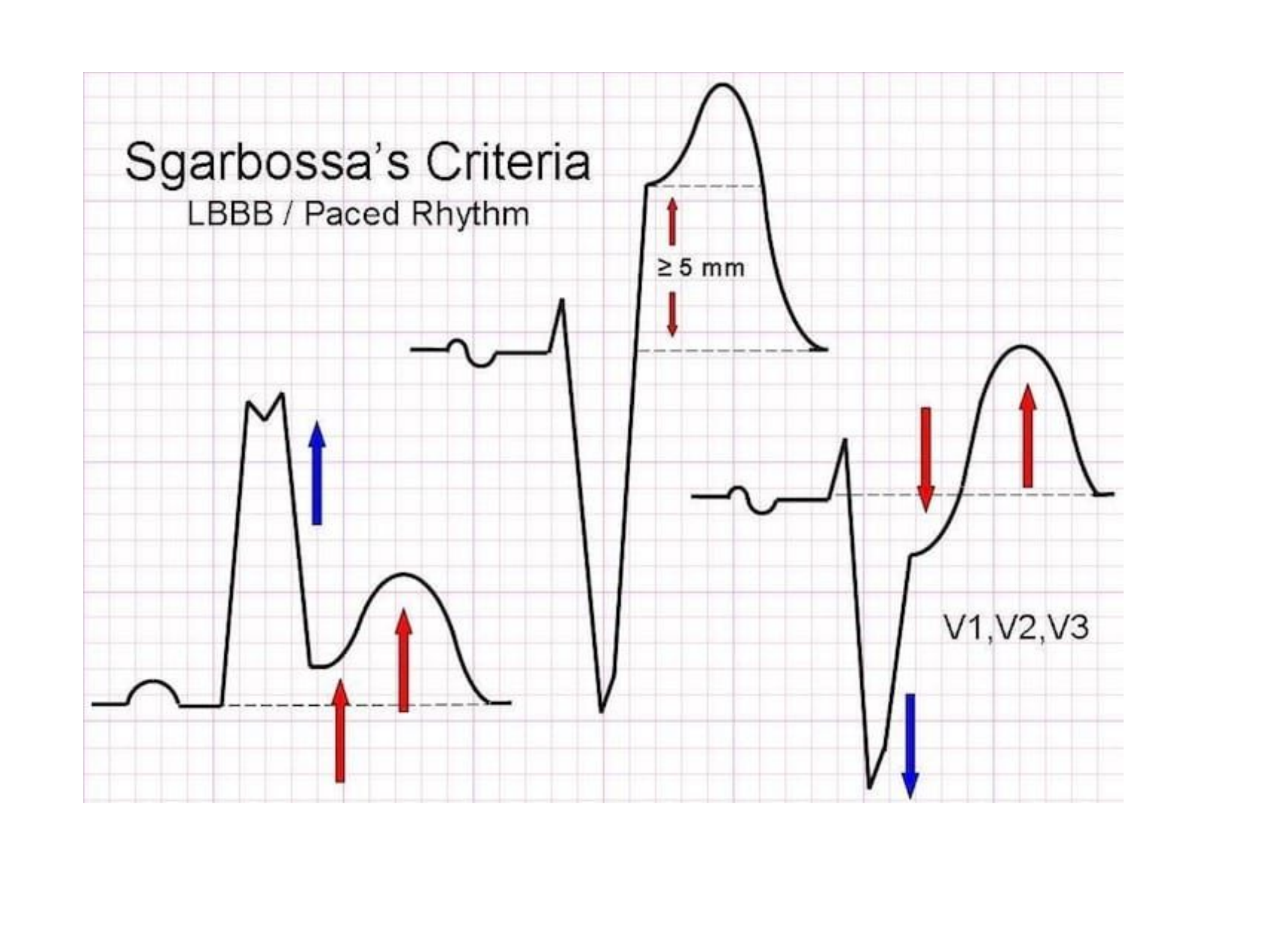

## Slide 26
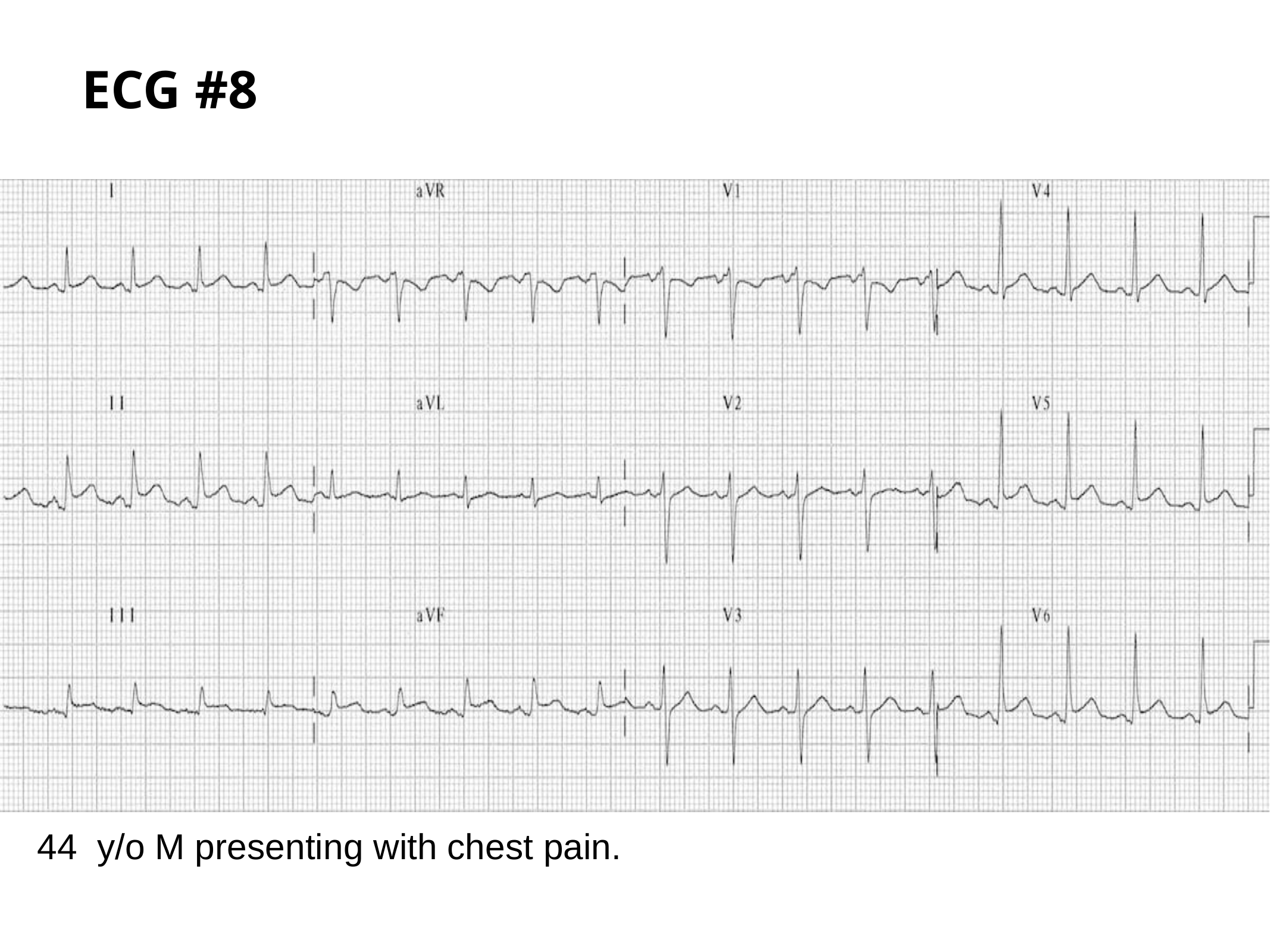

ECG #8
44 y/o M presenting with chest pain.

## Slide 27
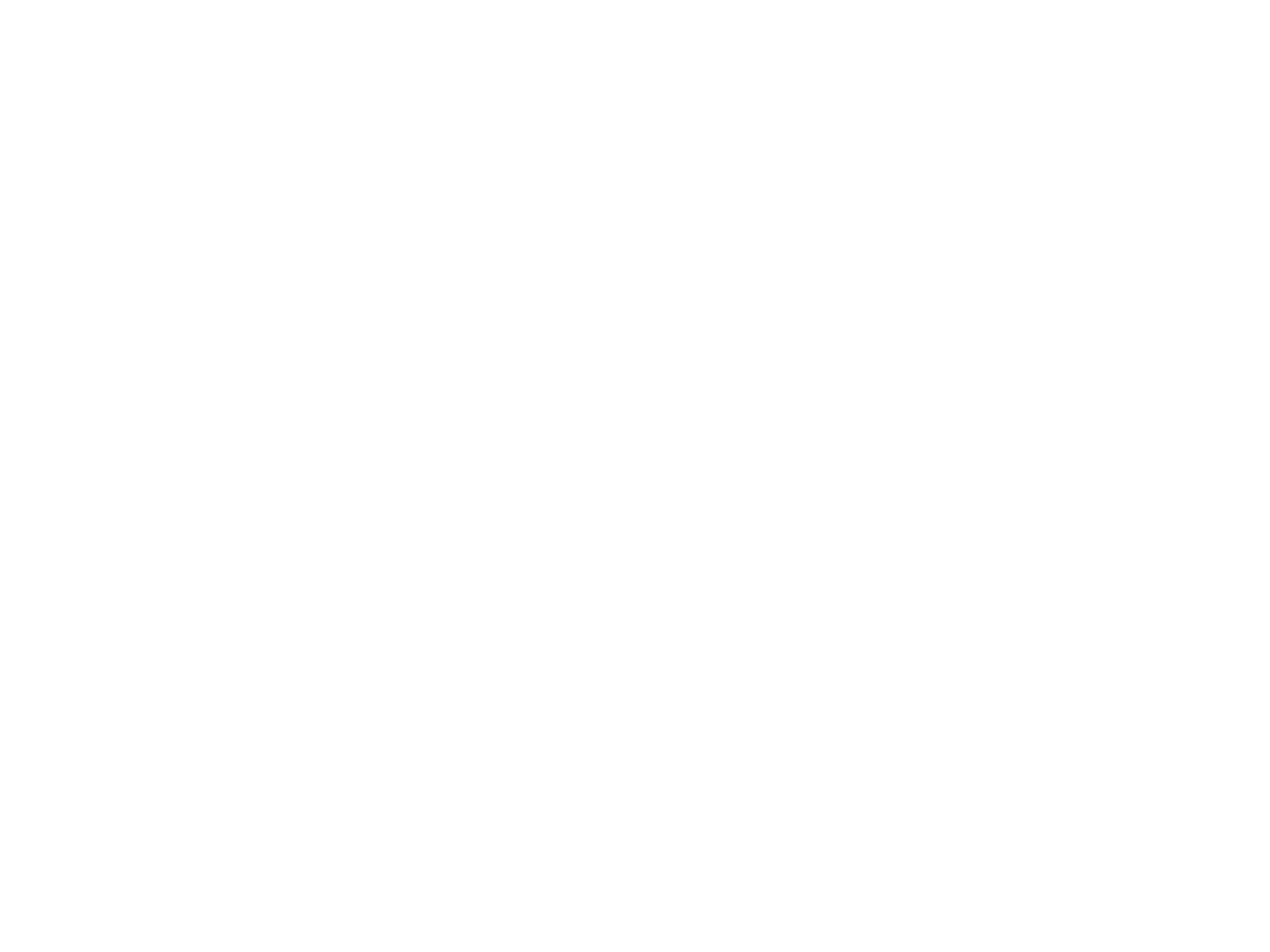

## Slide 28
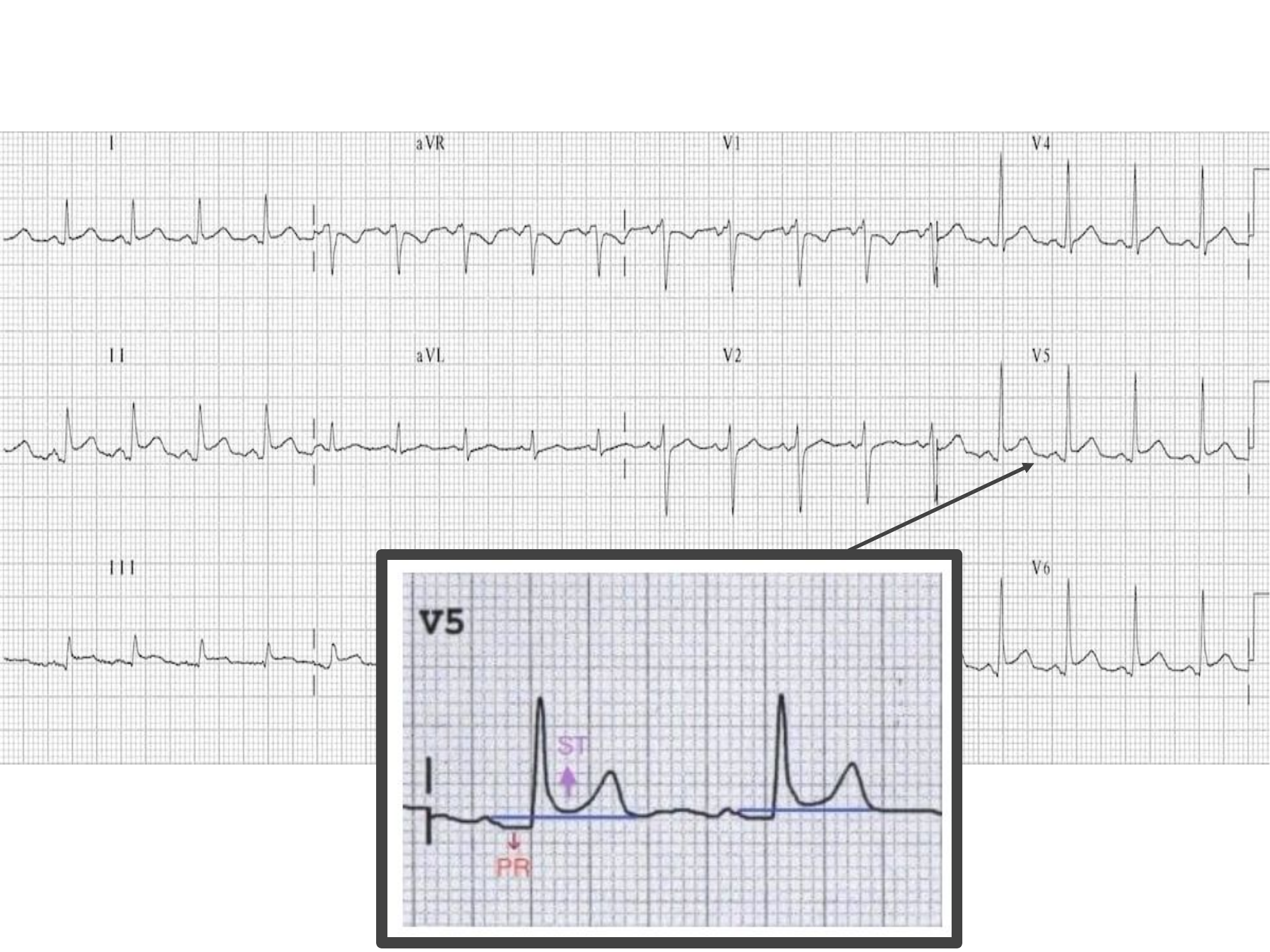

## Slide 29
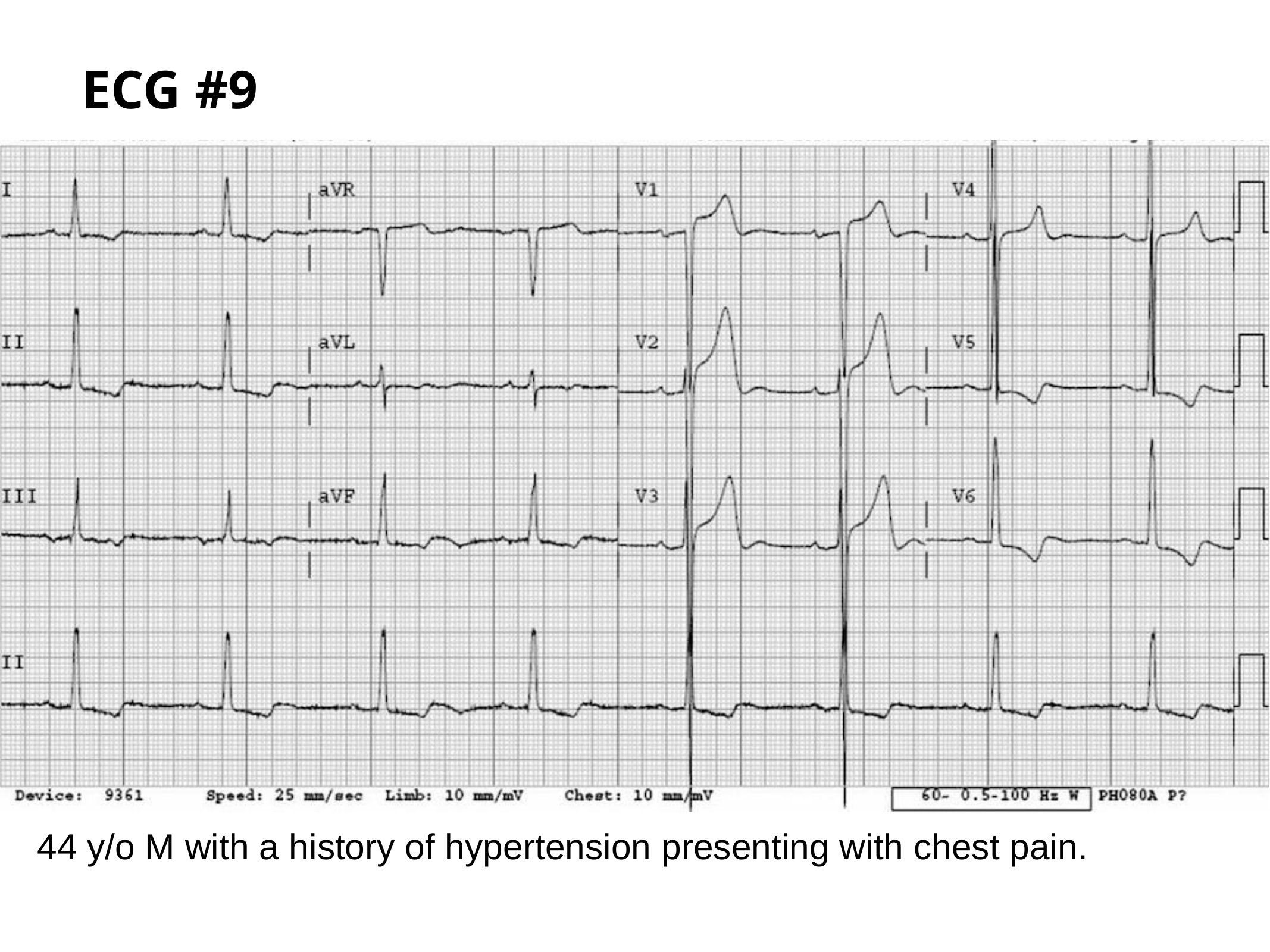

ECG #9
44 y/o M with a history of hypertension presenting with chest pain.

## Slide 30
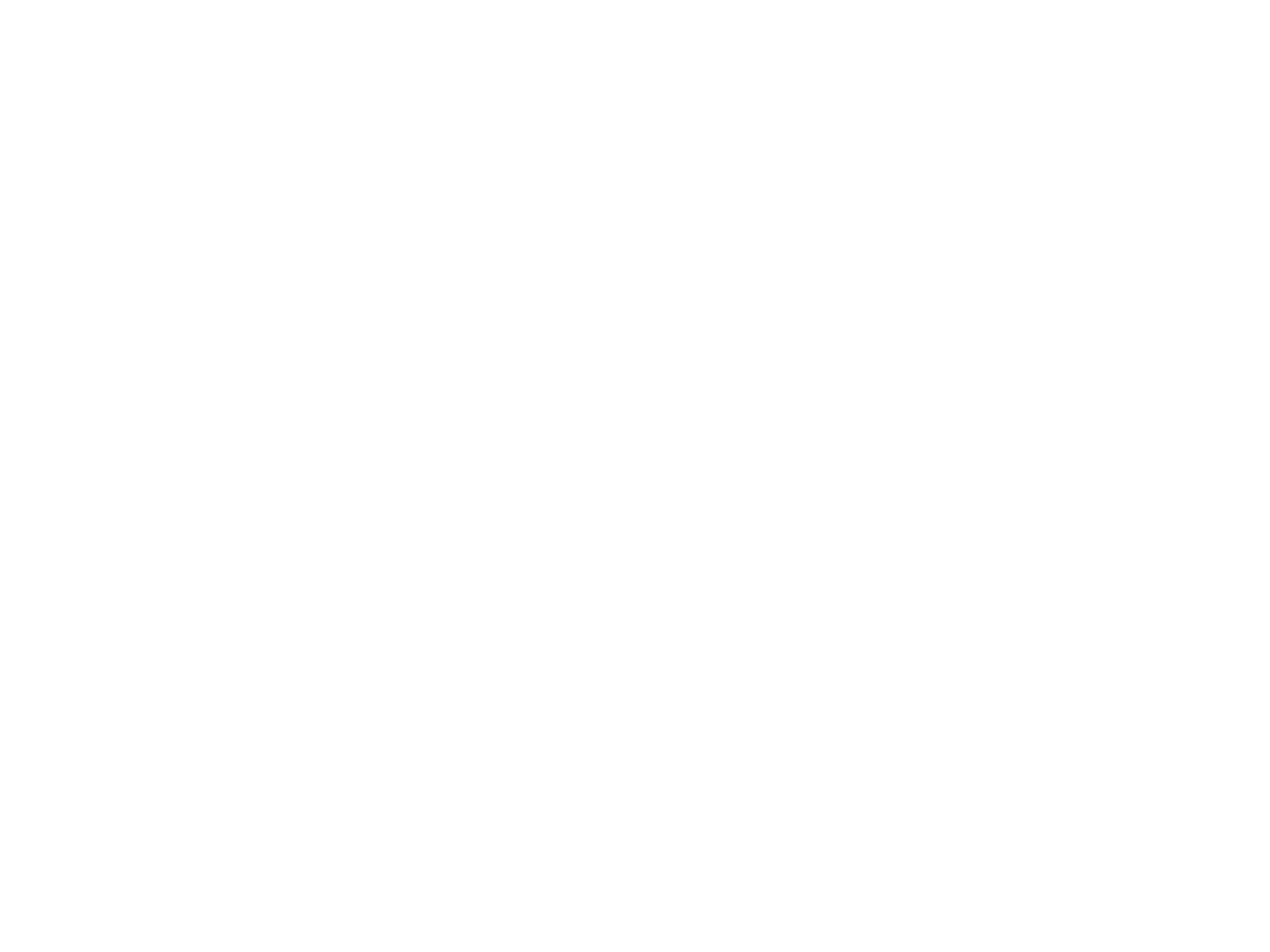

## Slide 31
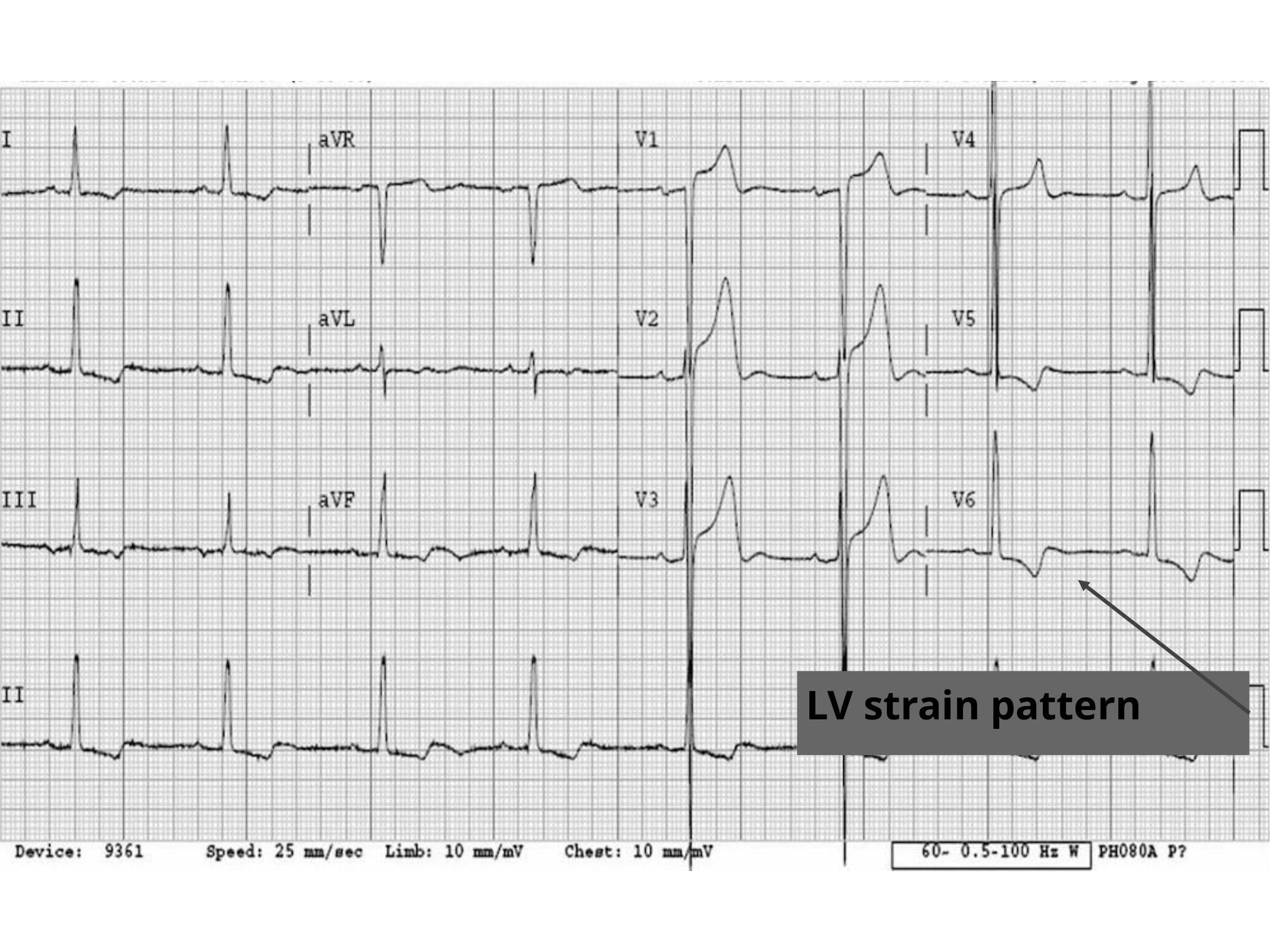

LV strain pattern

## Slide 32
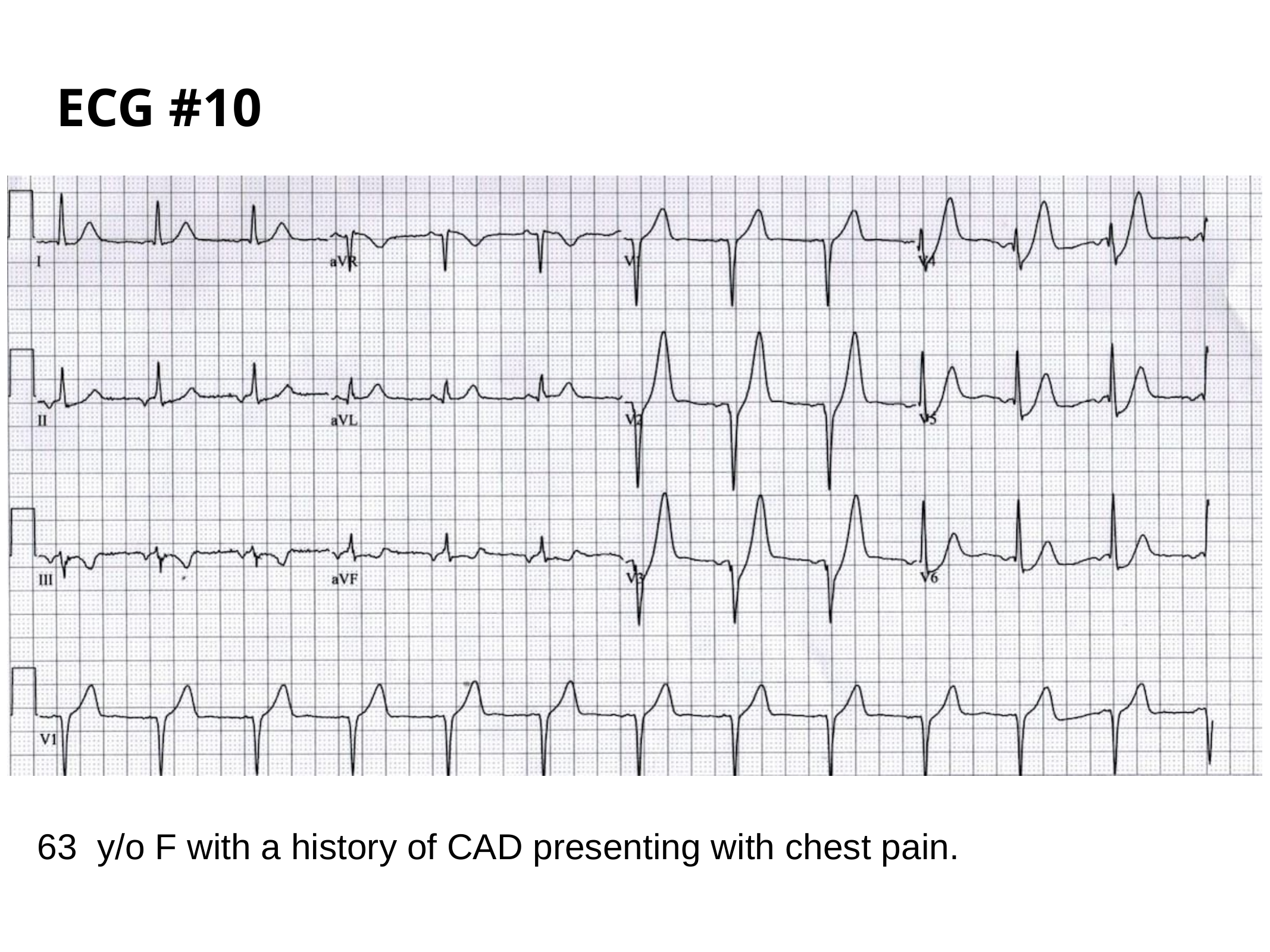

ECG #10
63 y/o F with a history of CAD presenting with chest pain.

## Slide 33
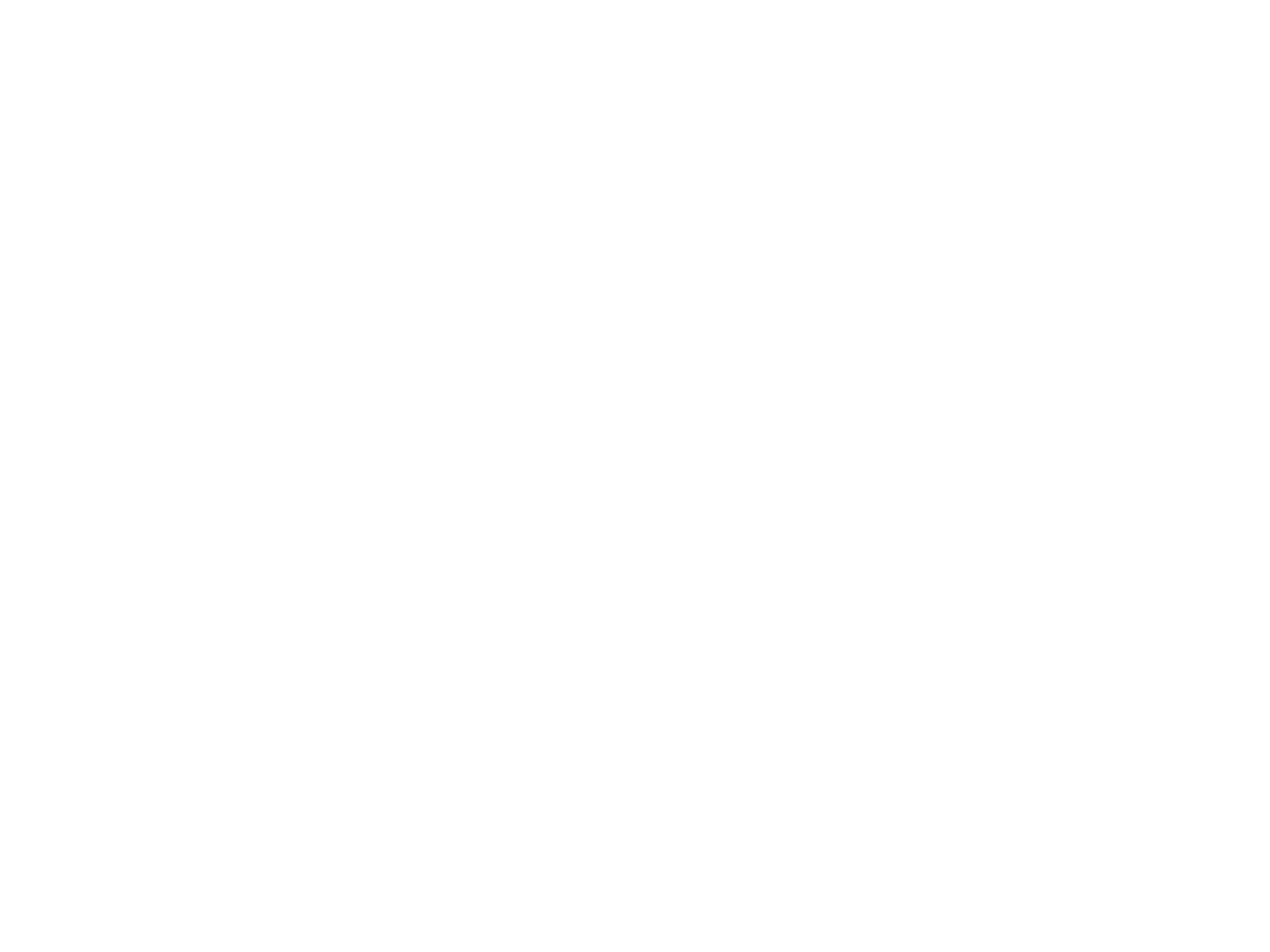

## Slide 34
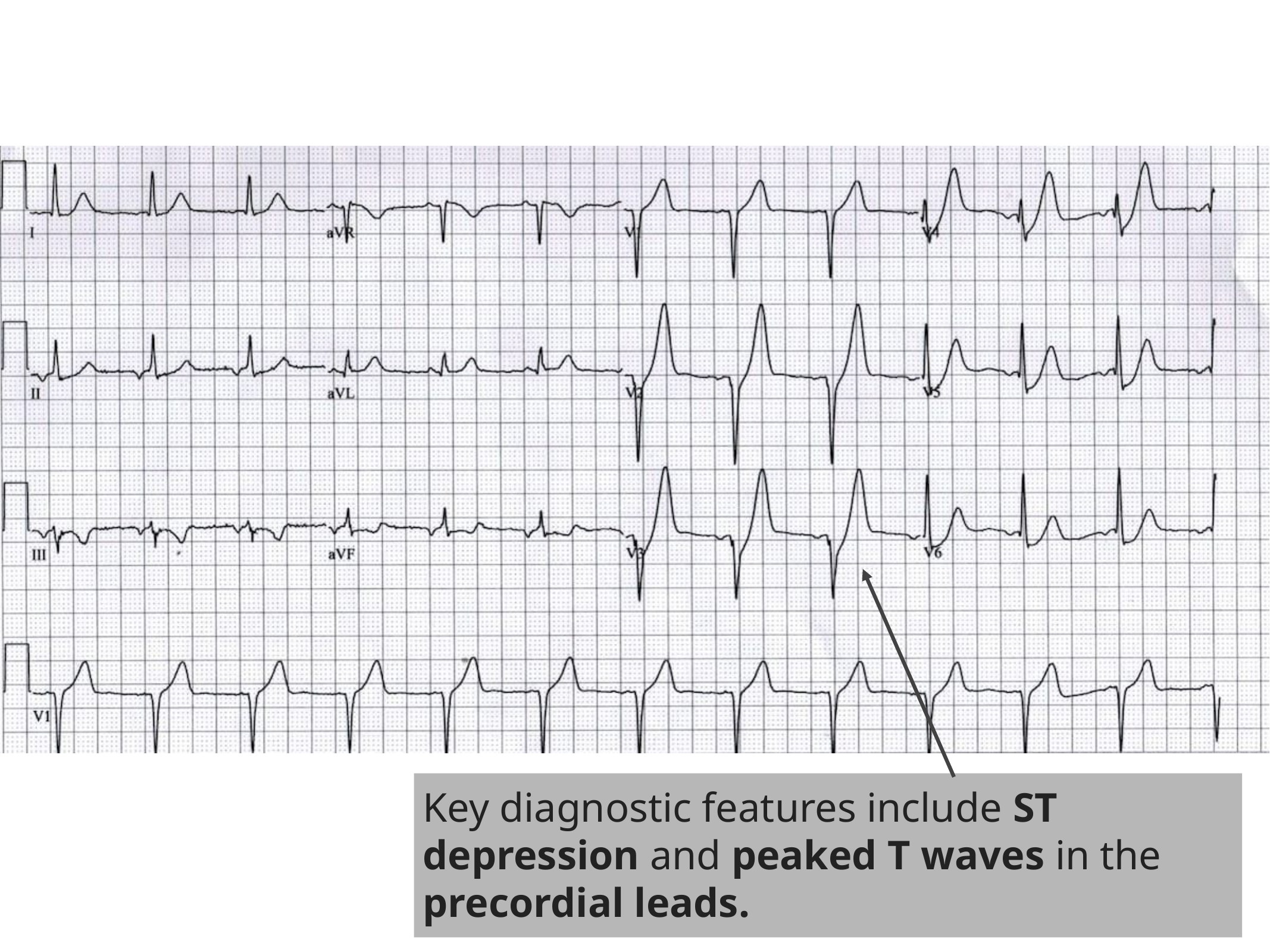

Key diagnostic features include ST depression and peaked T waves in the precordial leads.

## Slide 35
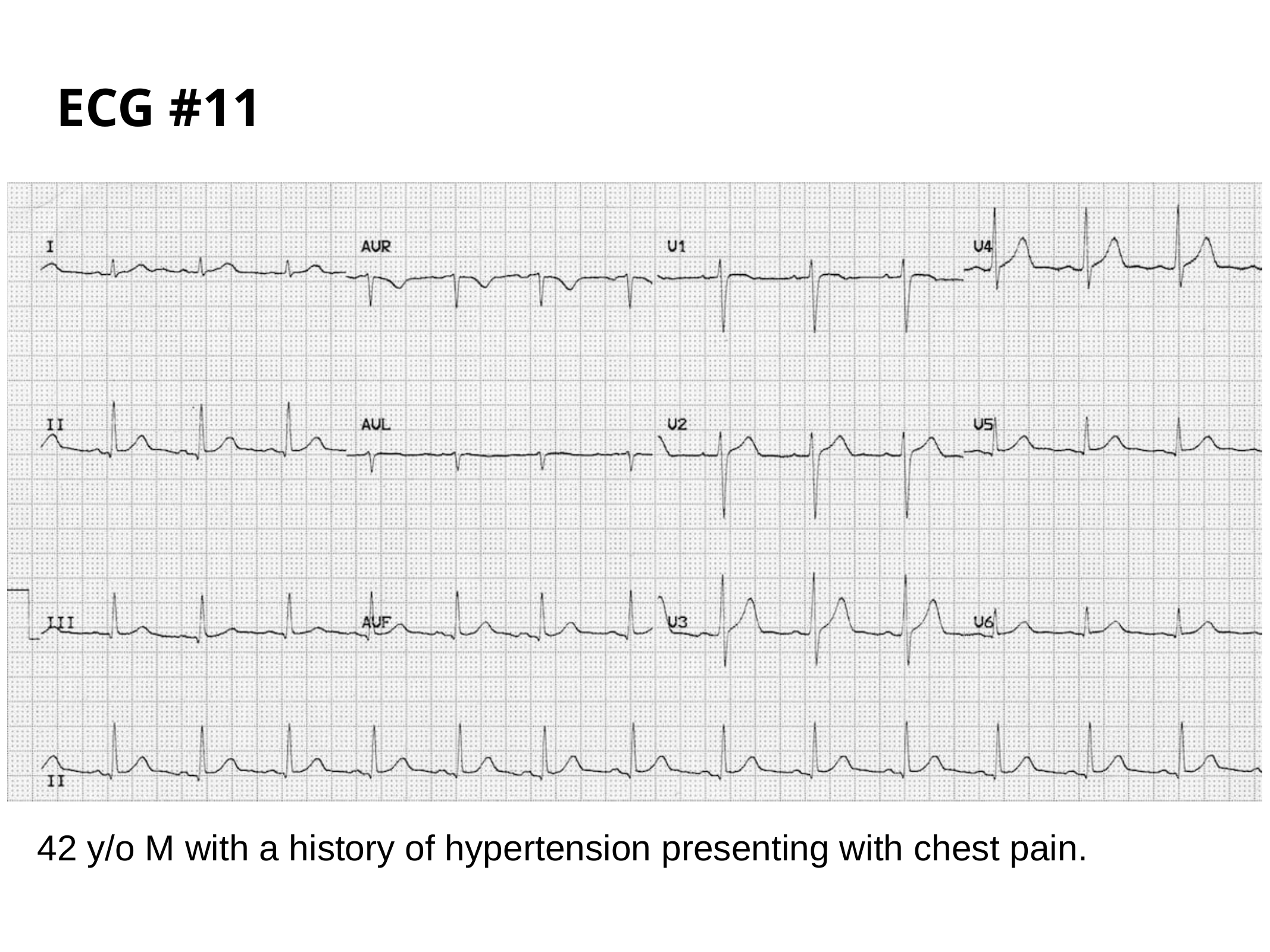

ECG #11
42 y/o M with a history of hypertension presenting with chest pain.

## Slide 36
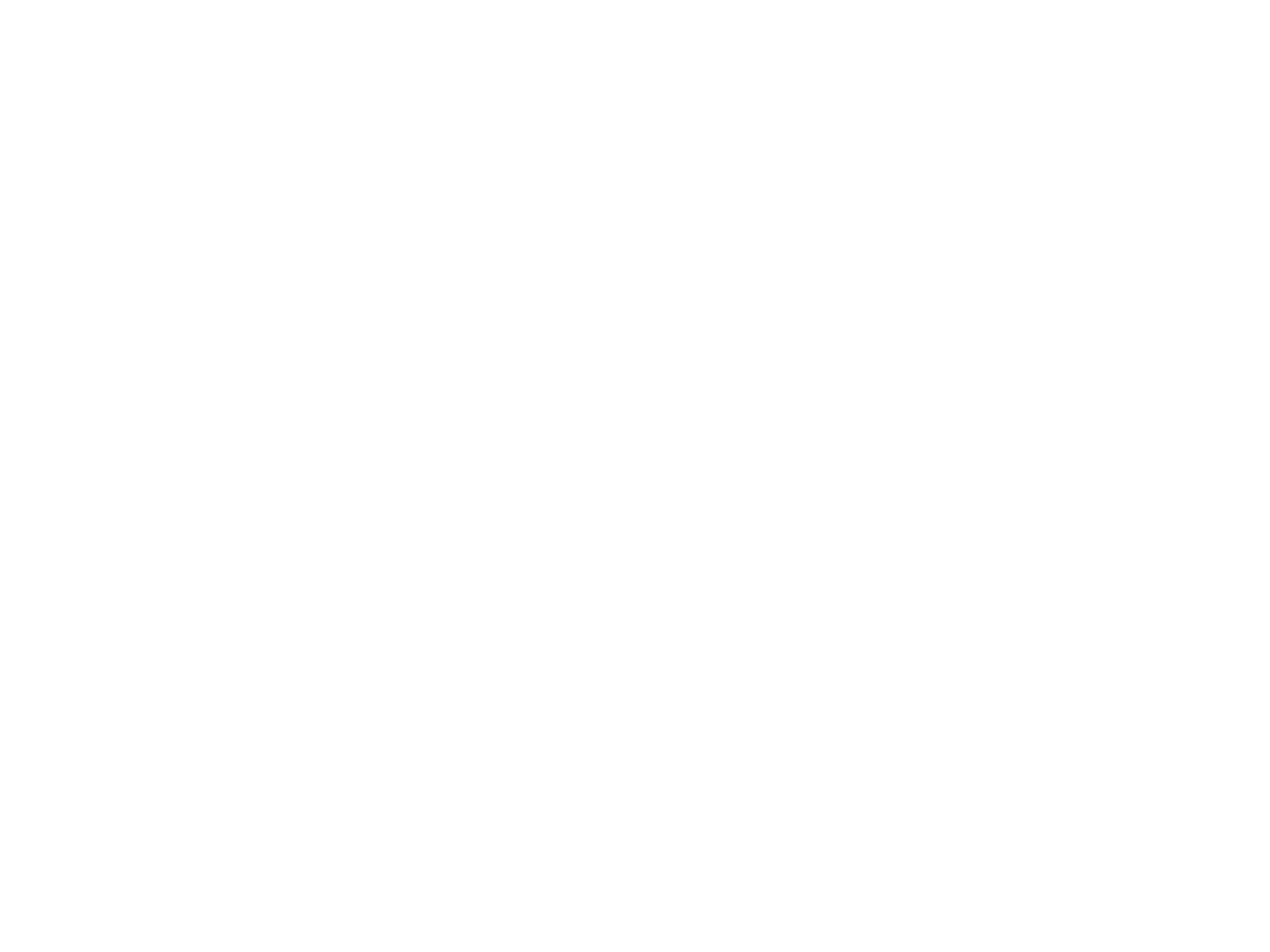

## Slide 37
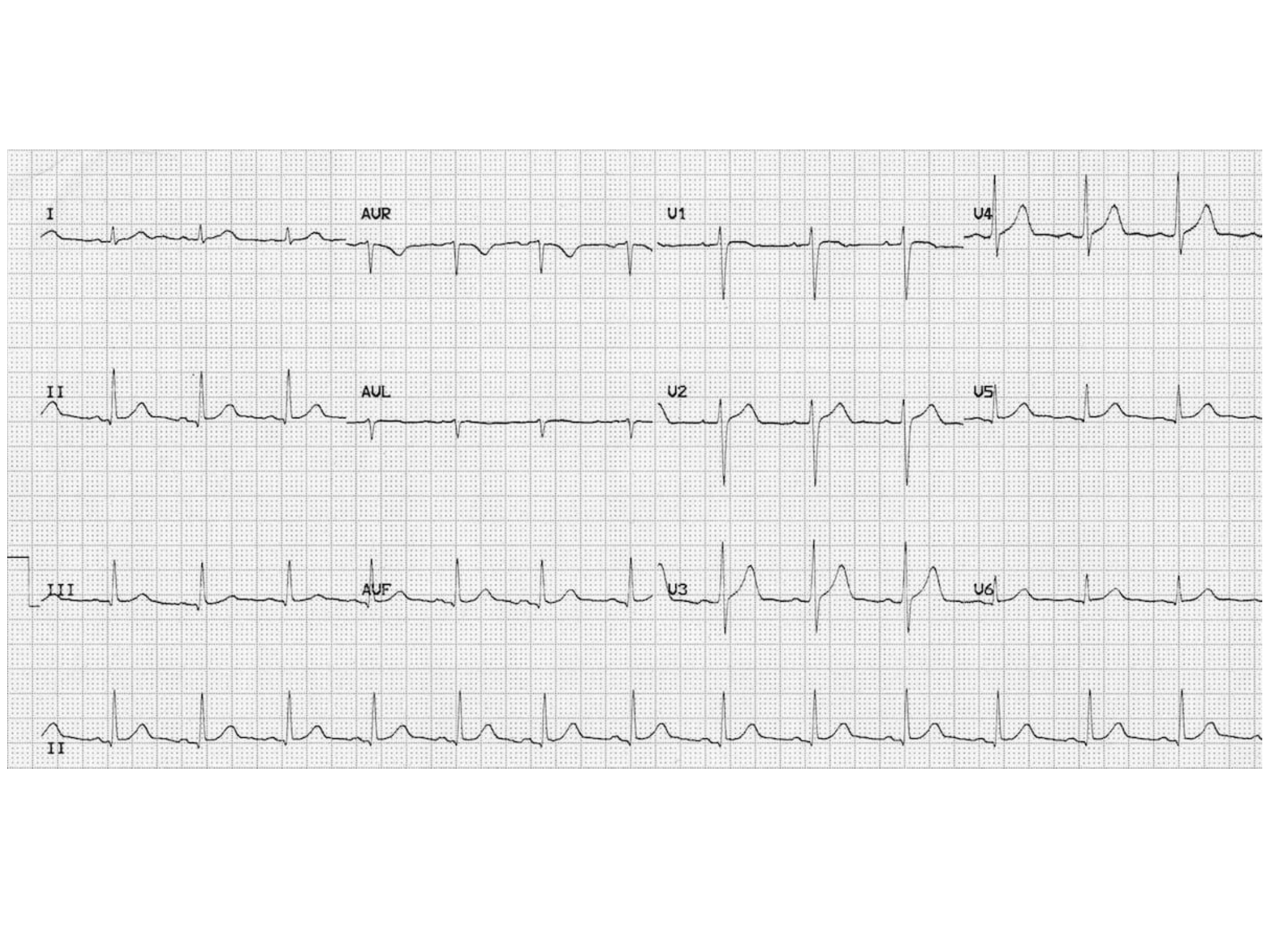

## Slide 38
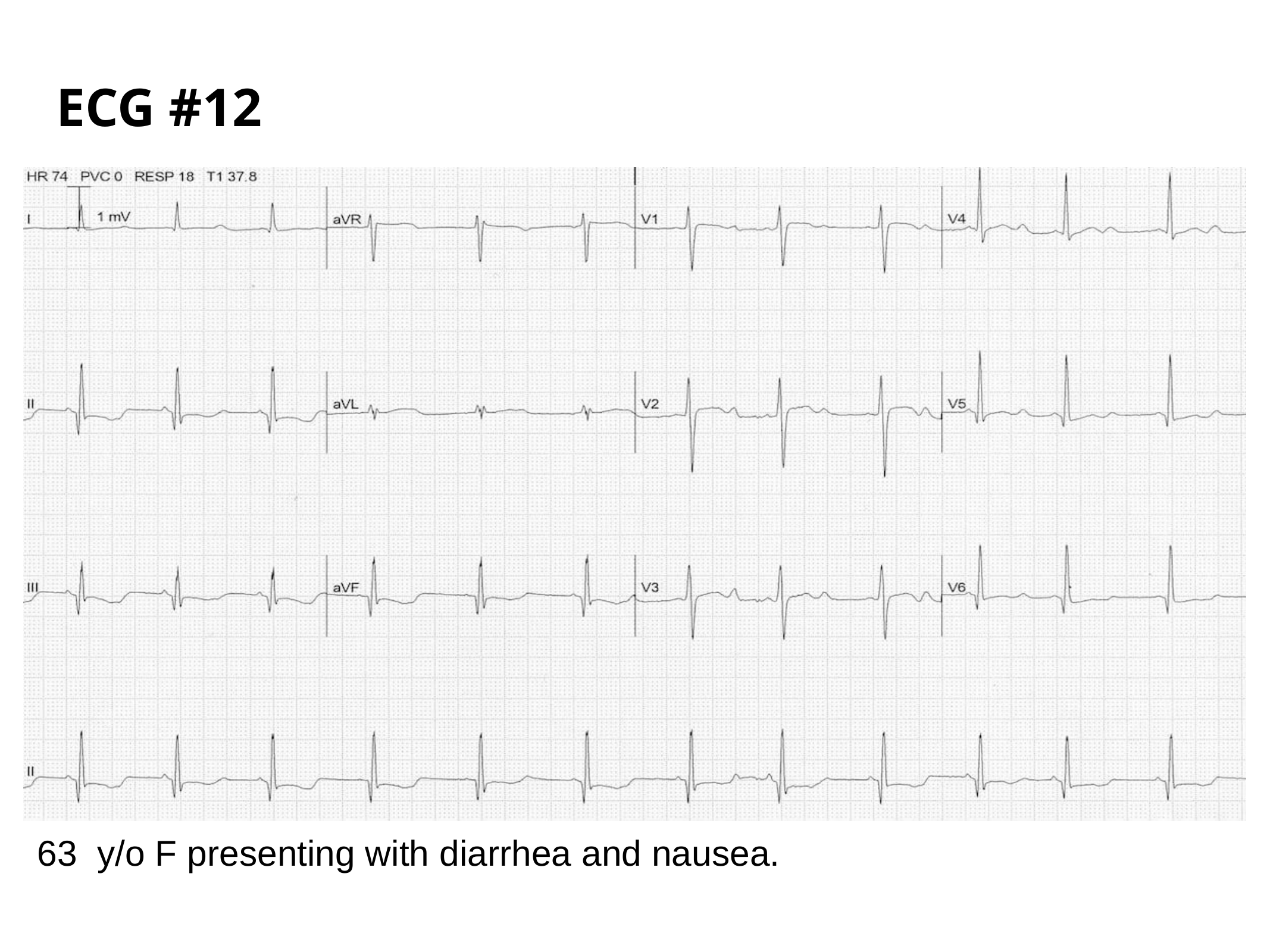

ECG #12
63 y/o F presenting with diarrhea and nausea.

## Slide 39
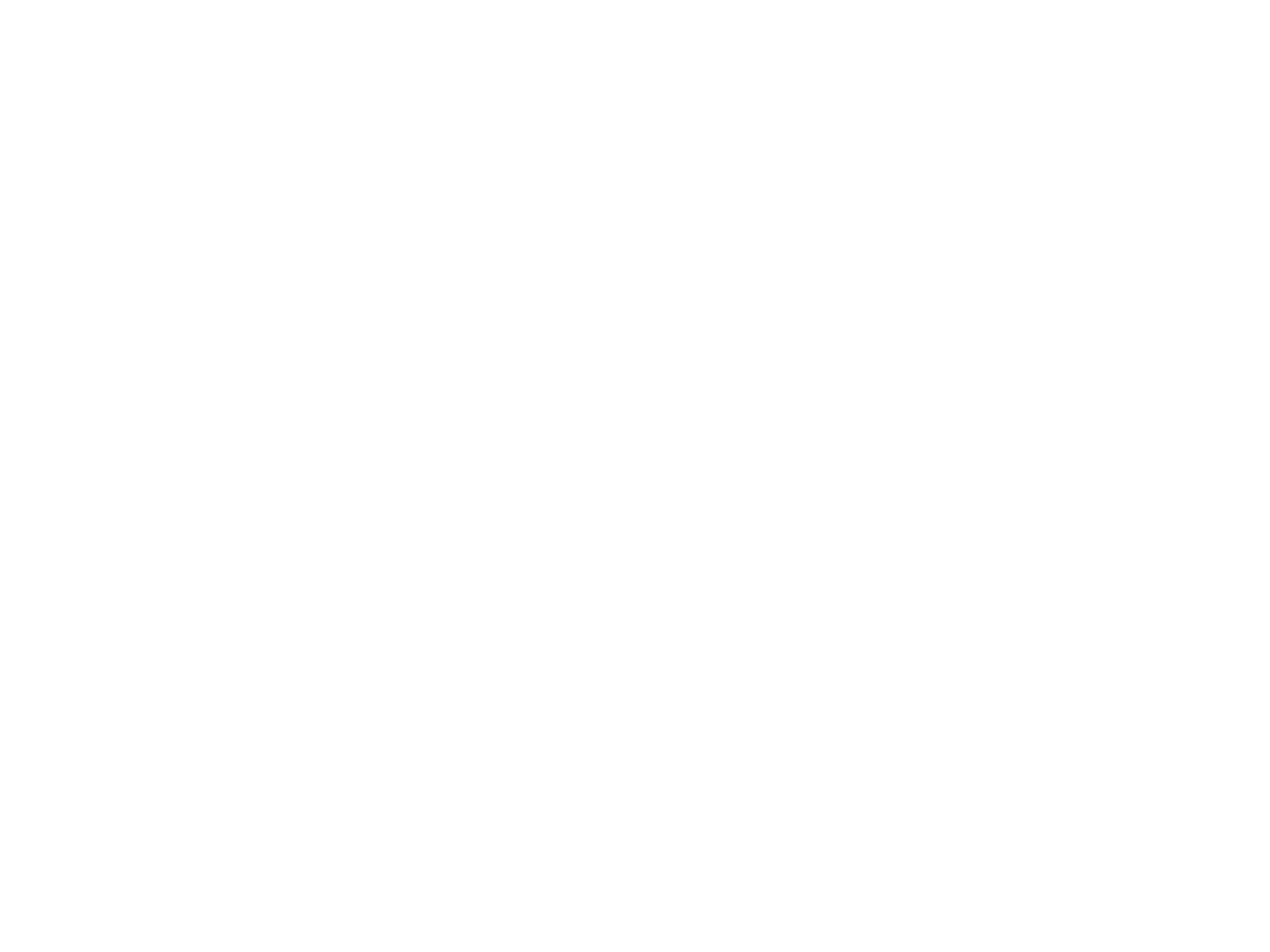

## Slide 40
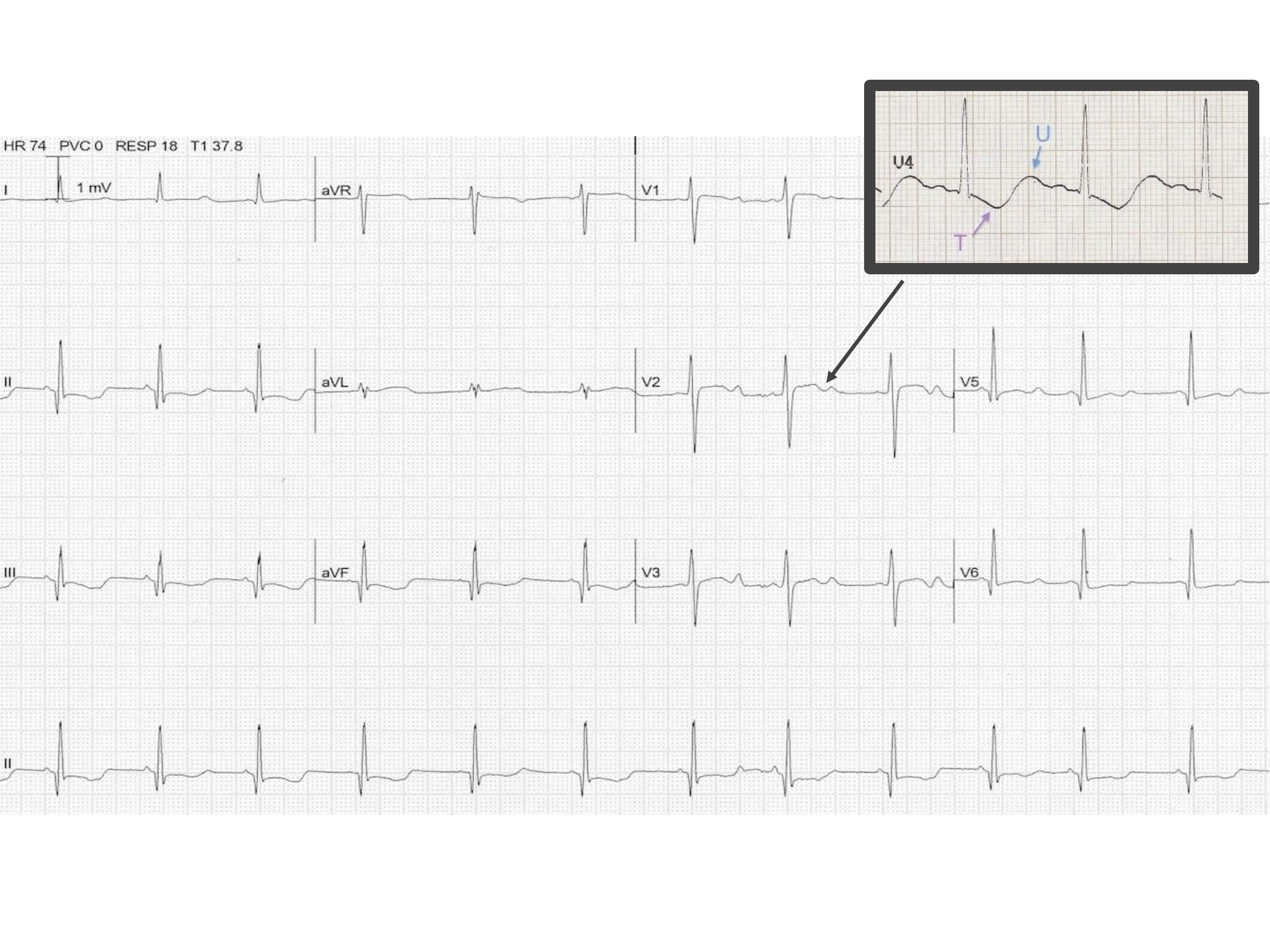

## Slide 41
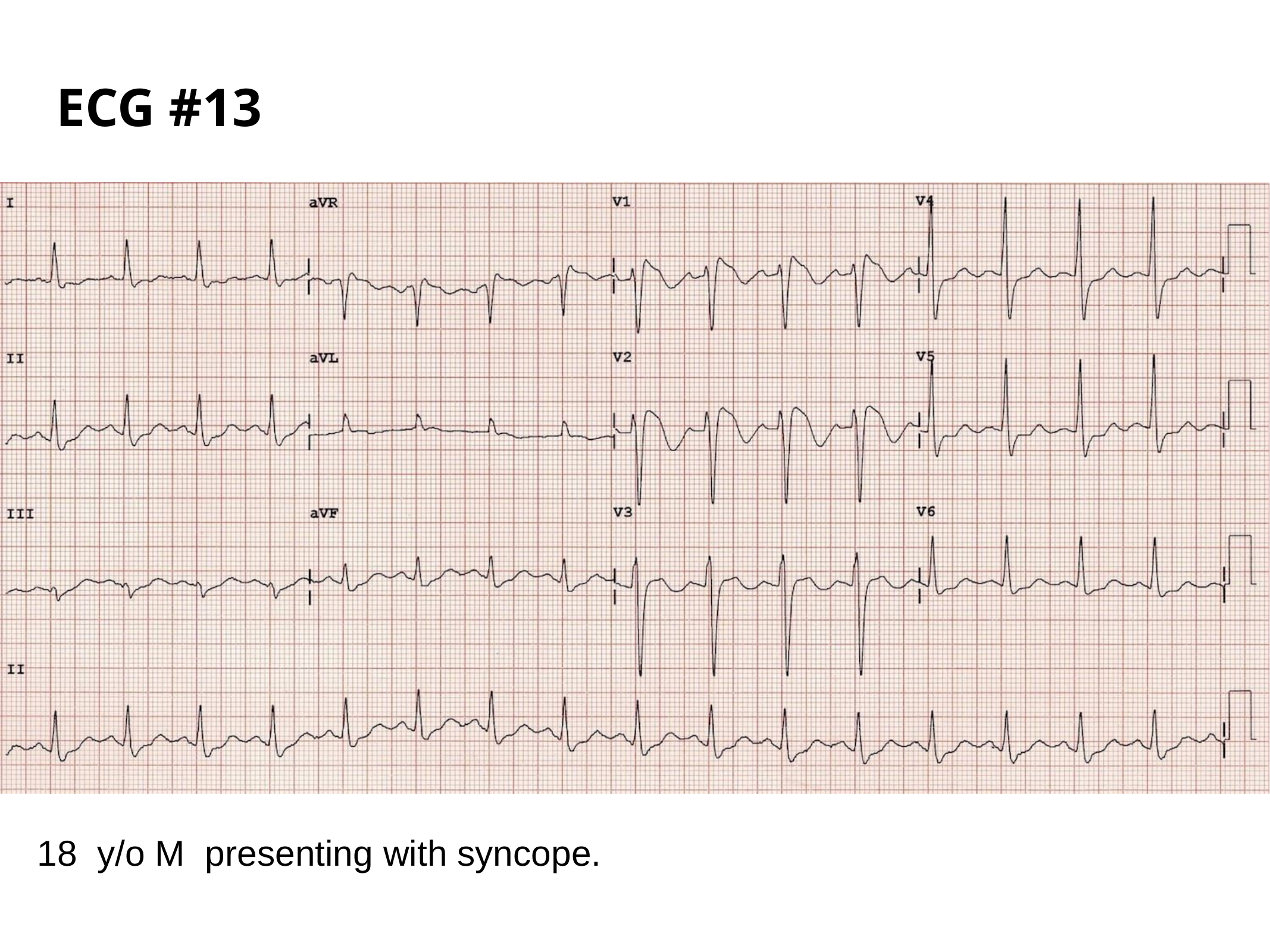

ECG #13
18 y/o M presenting with syncope.

## Slide 42
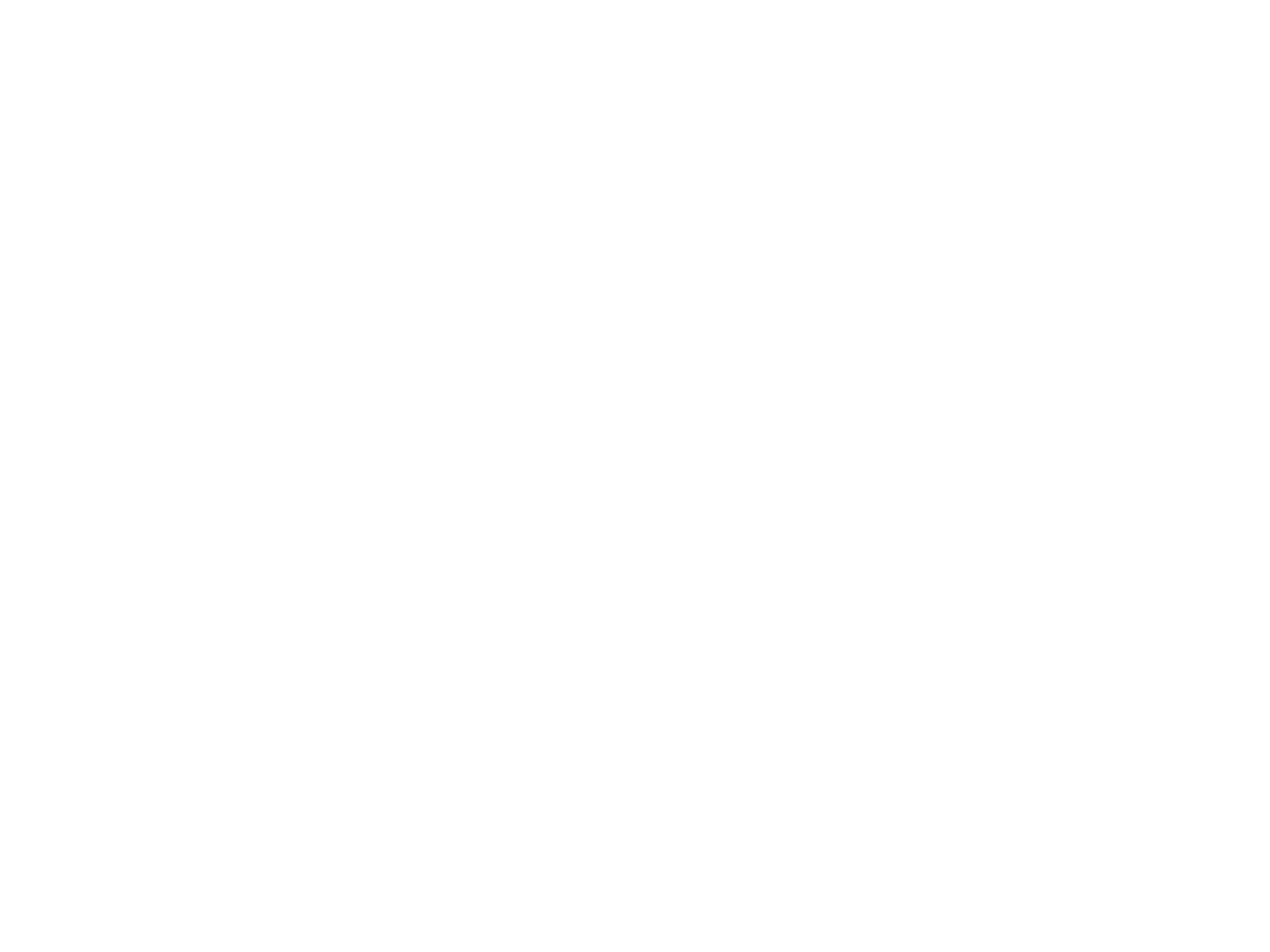

## Slide 43
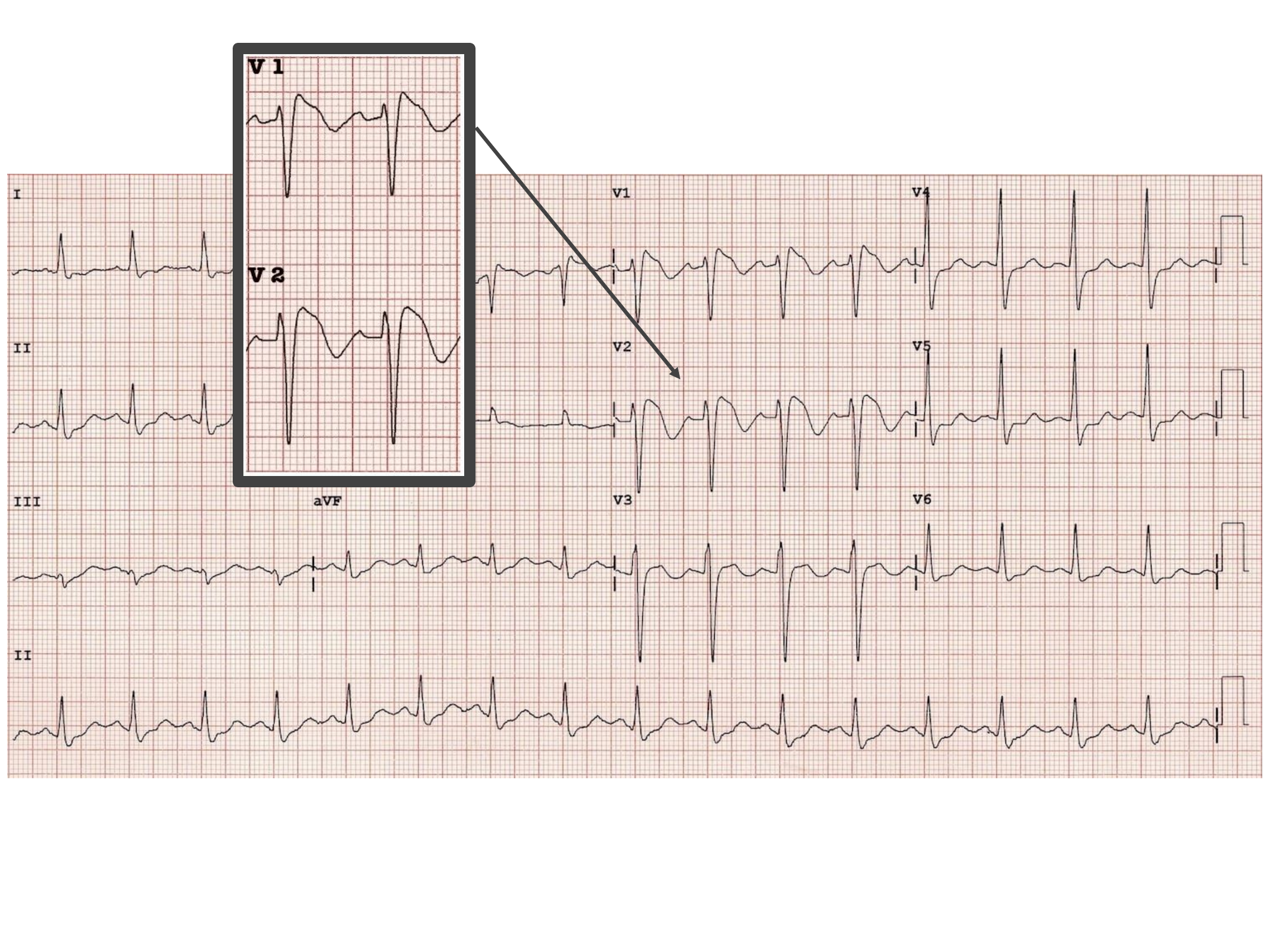

## Slide 44
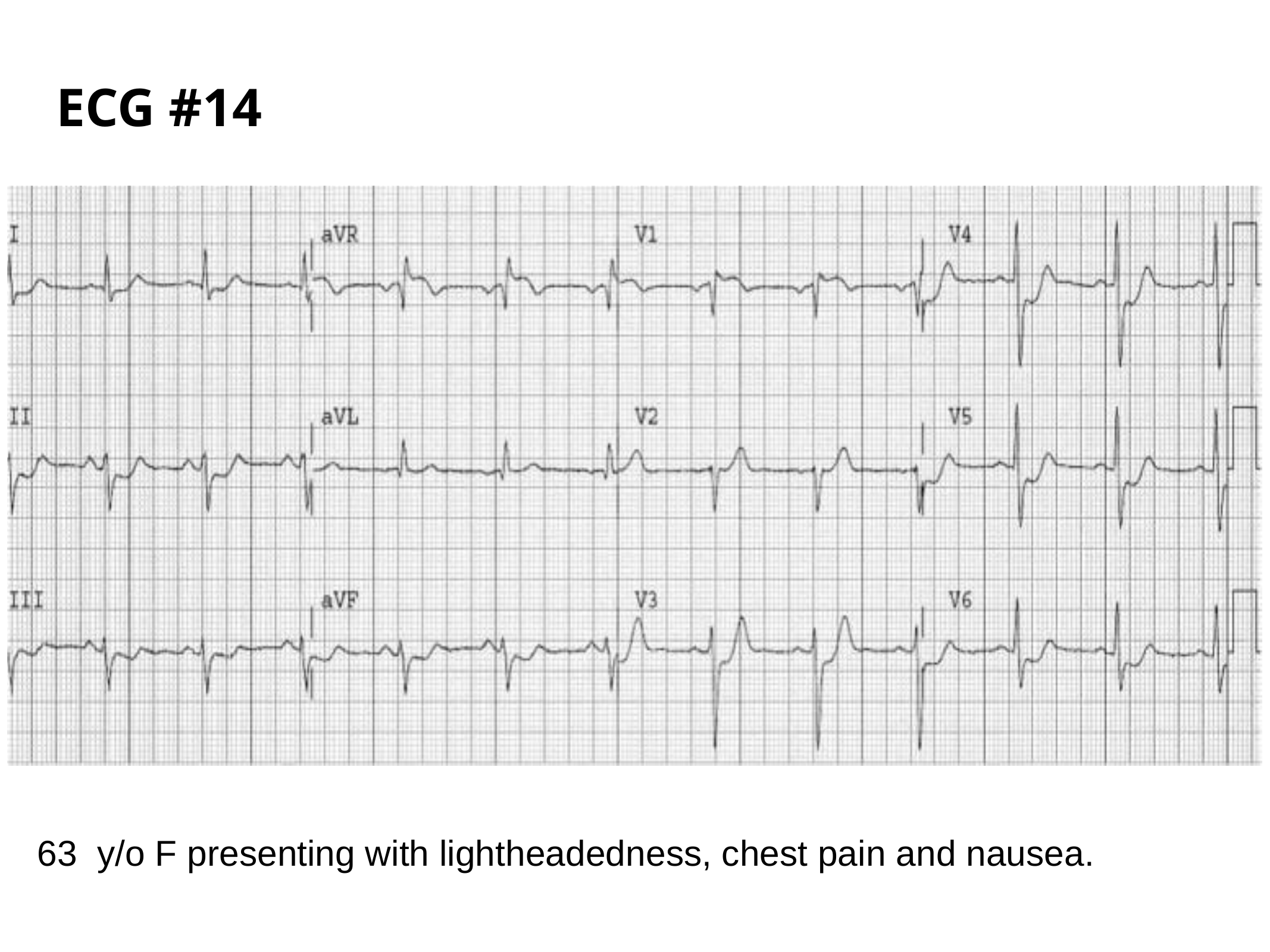

ECG #14
63 y/o F presenting with lightheadedness, chest pain and nausea.

## Slide 45
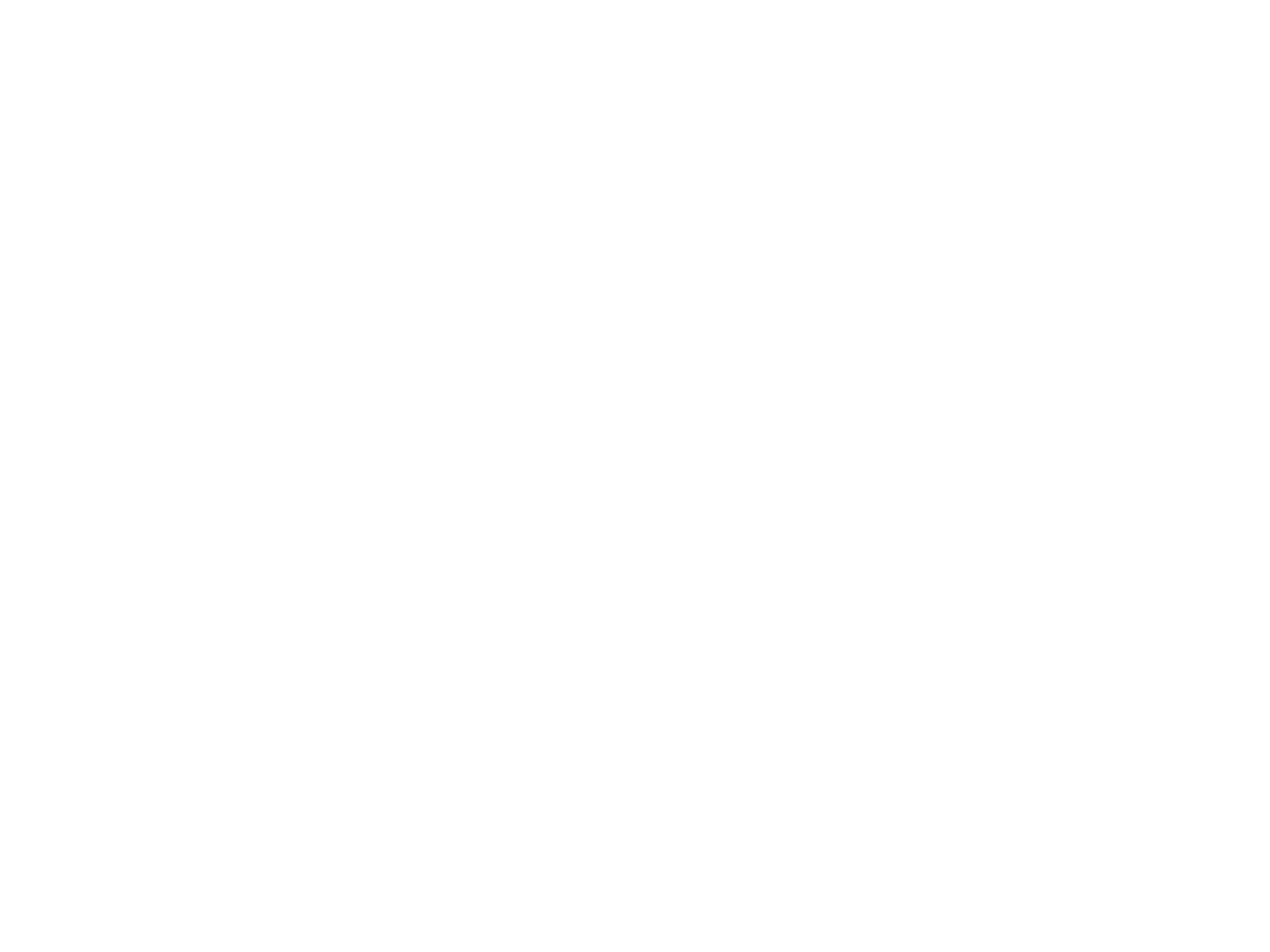

## Slide 46
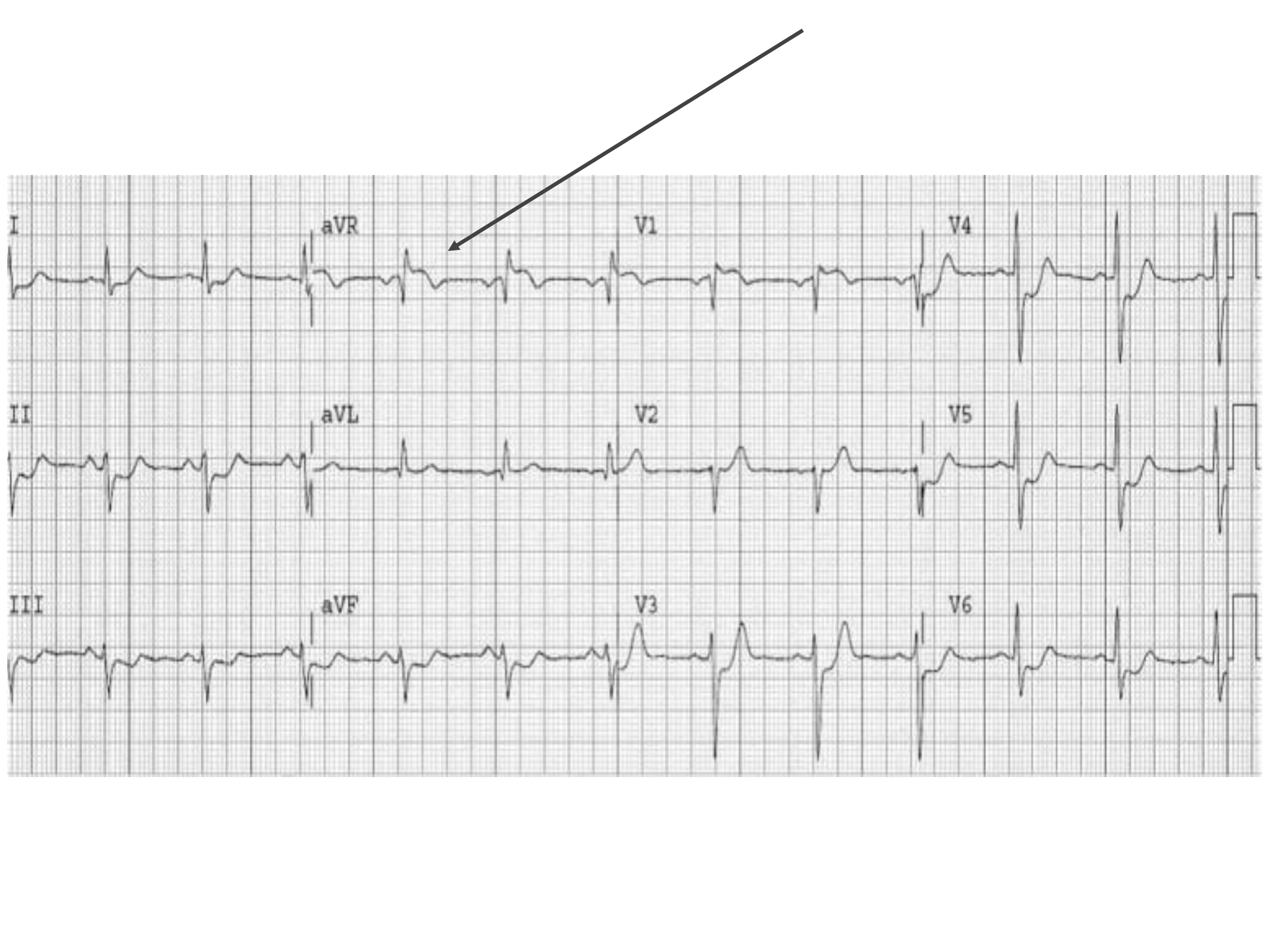

## Slide 47
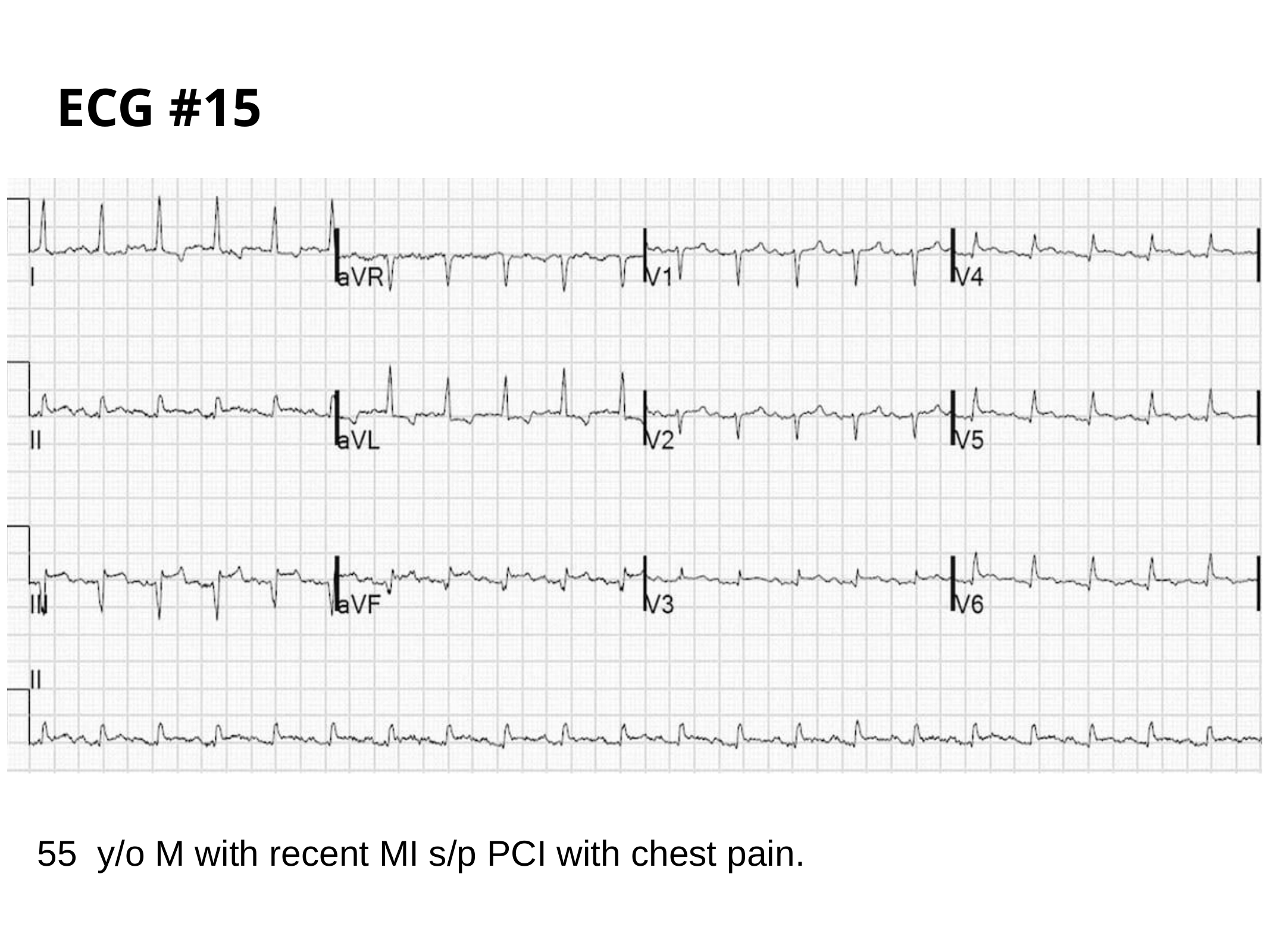

ECG #15
55 y/o M with recent MI s/p PCI with chest pain.

## Slide 48
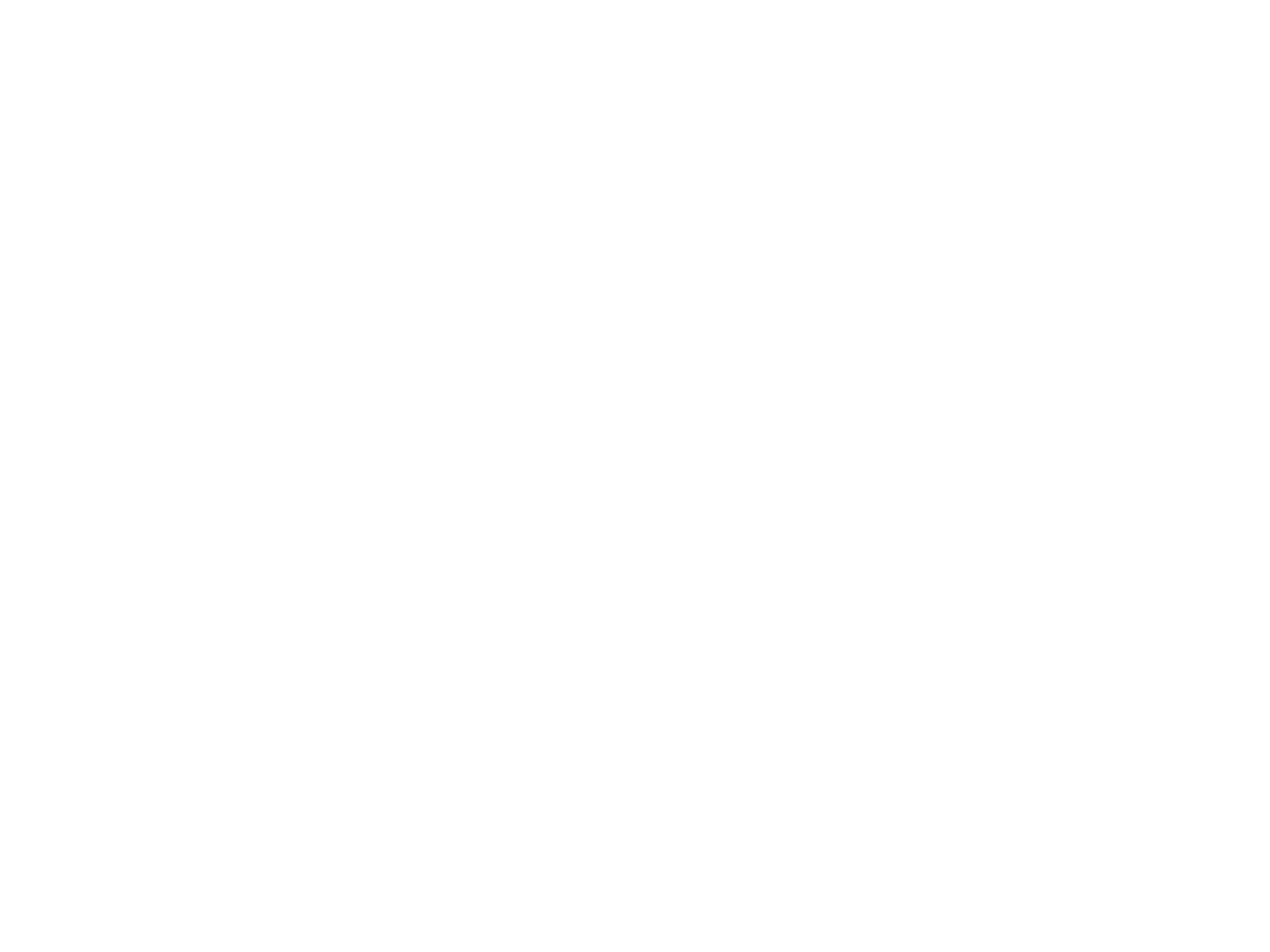

## Slide 49
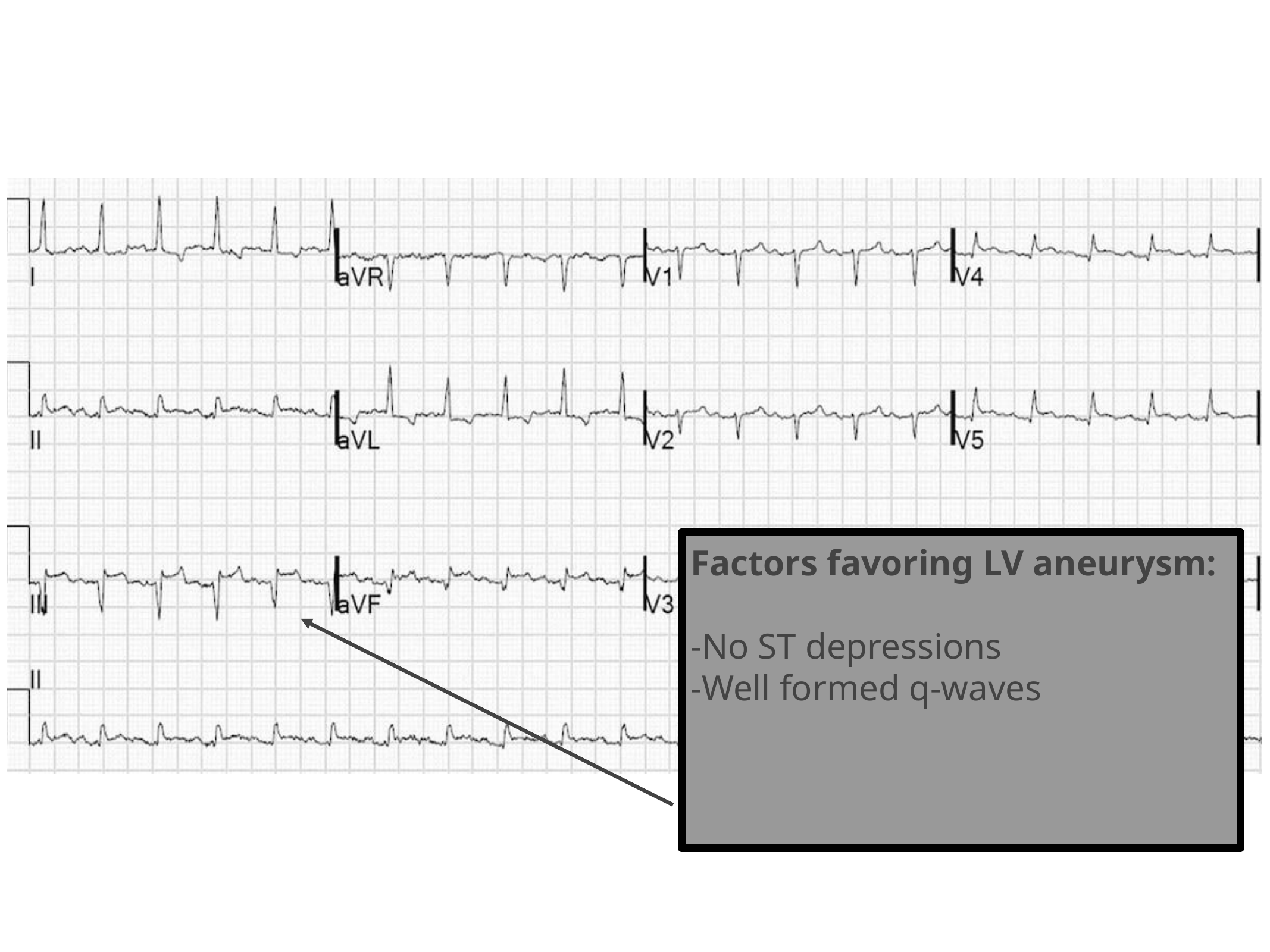

Factors favoring LV aneurysm:
-No ST depressions
-Well formed q-waves

## Slide 50
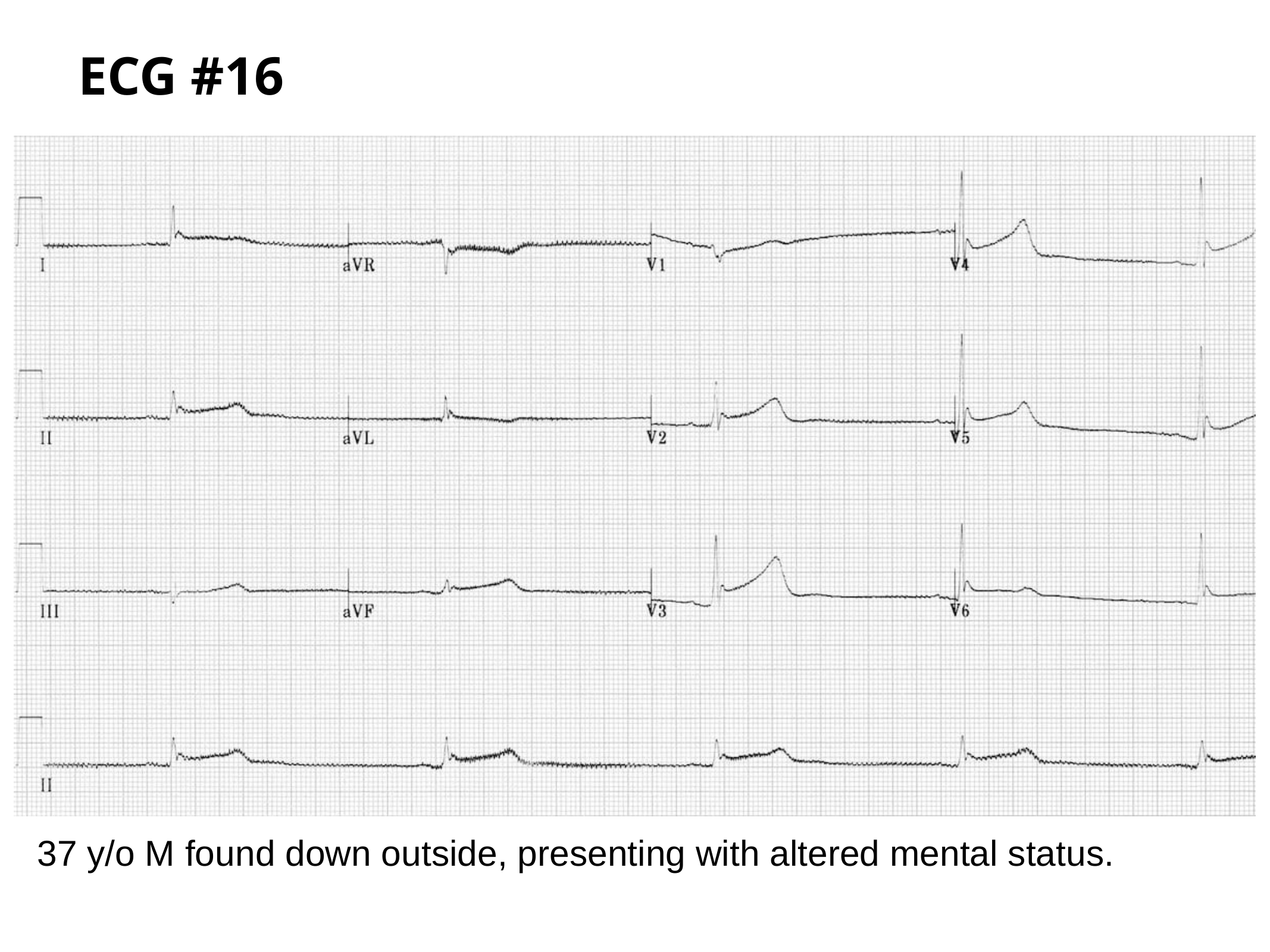

ECG #16
37 y/o M found down outside, presenting with altered mental status.

## Slide 51
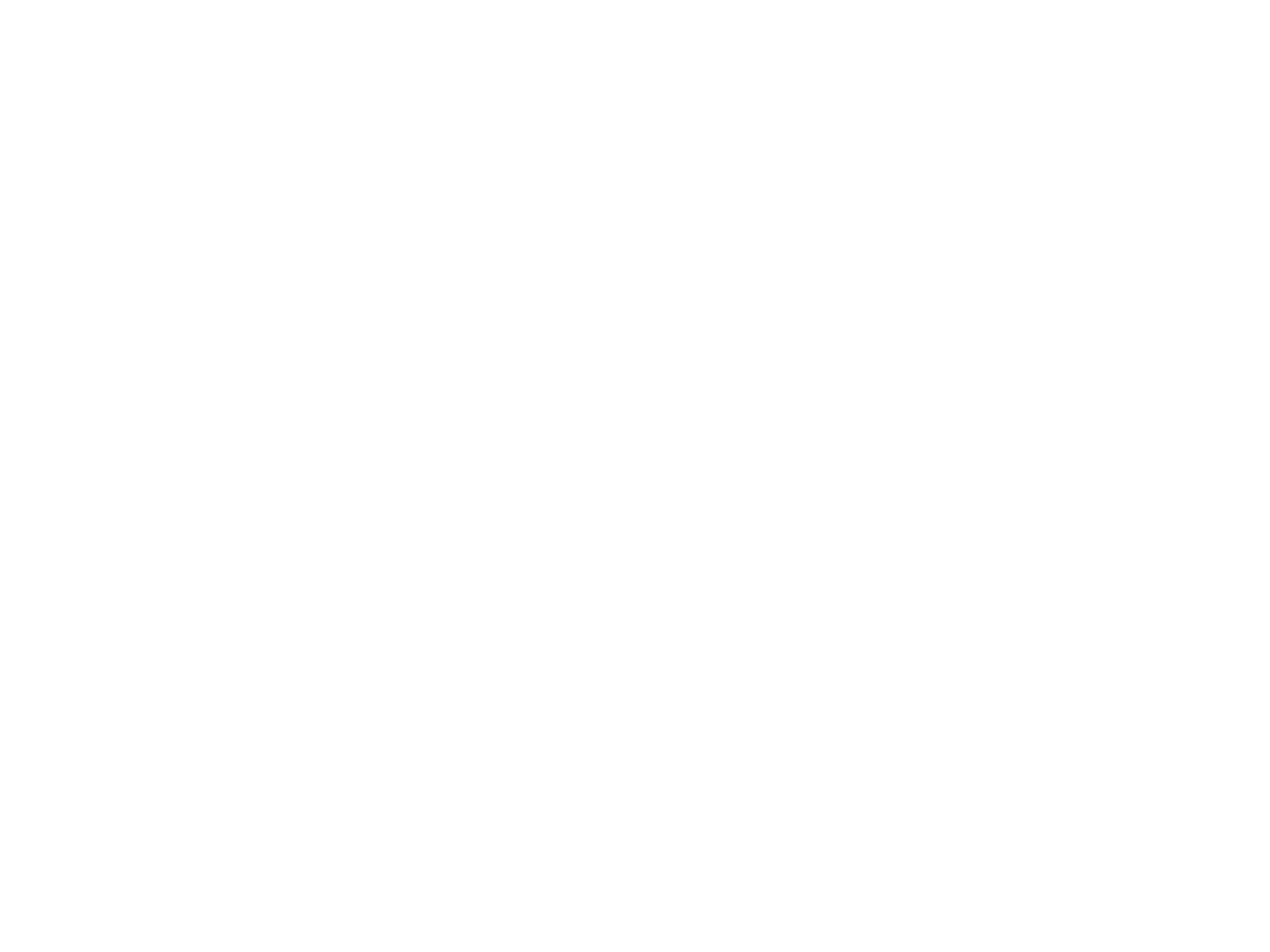

## Slide 52
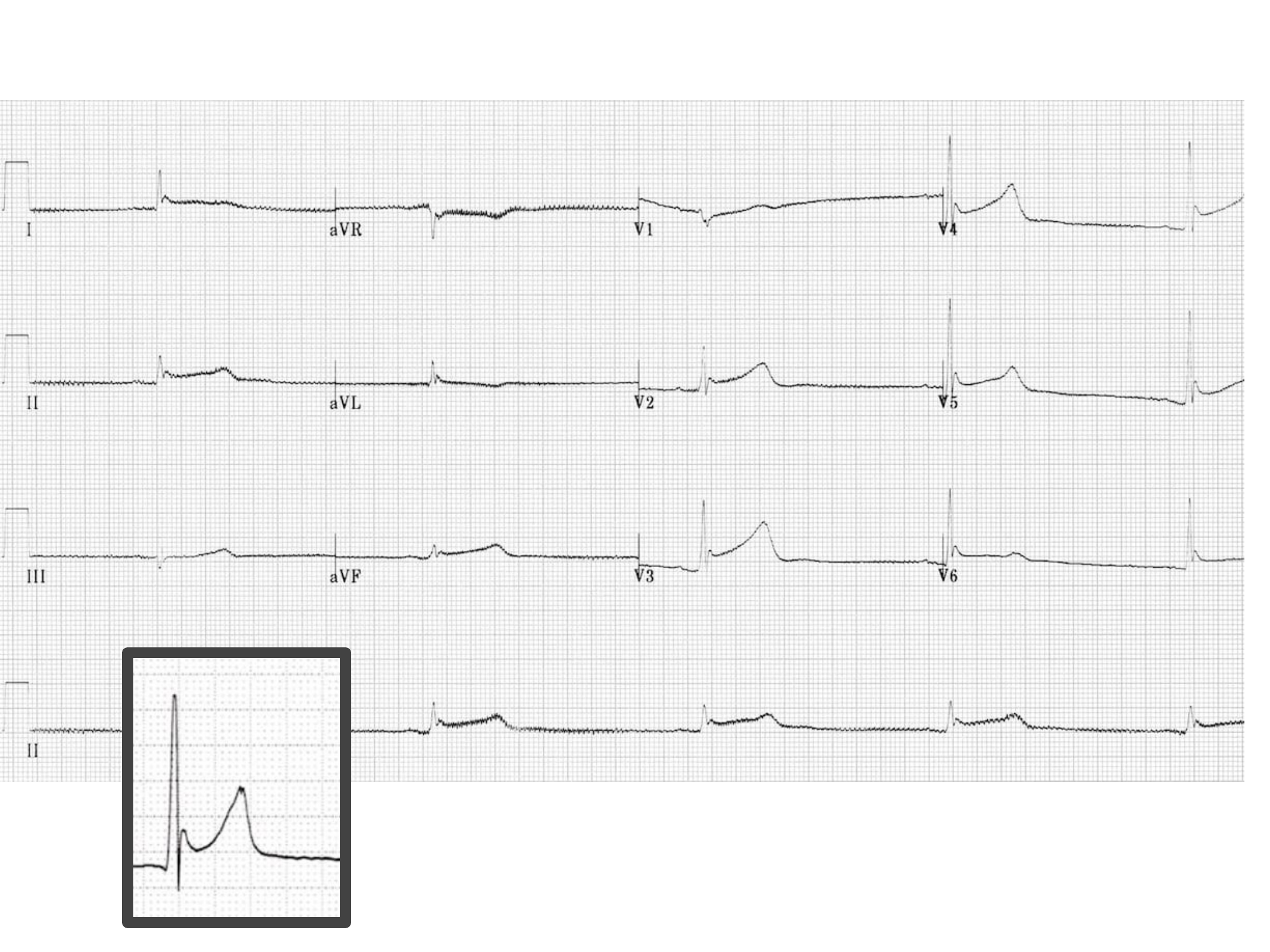

## Slide 53
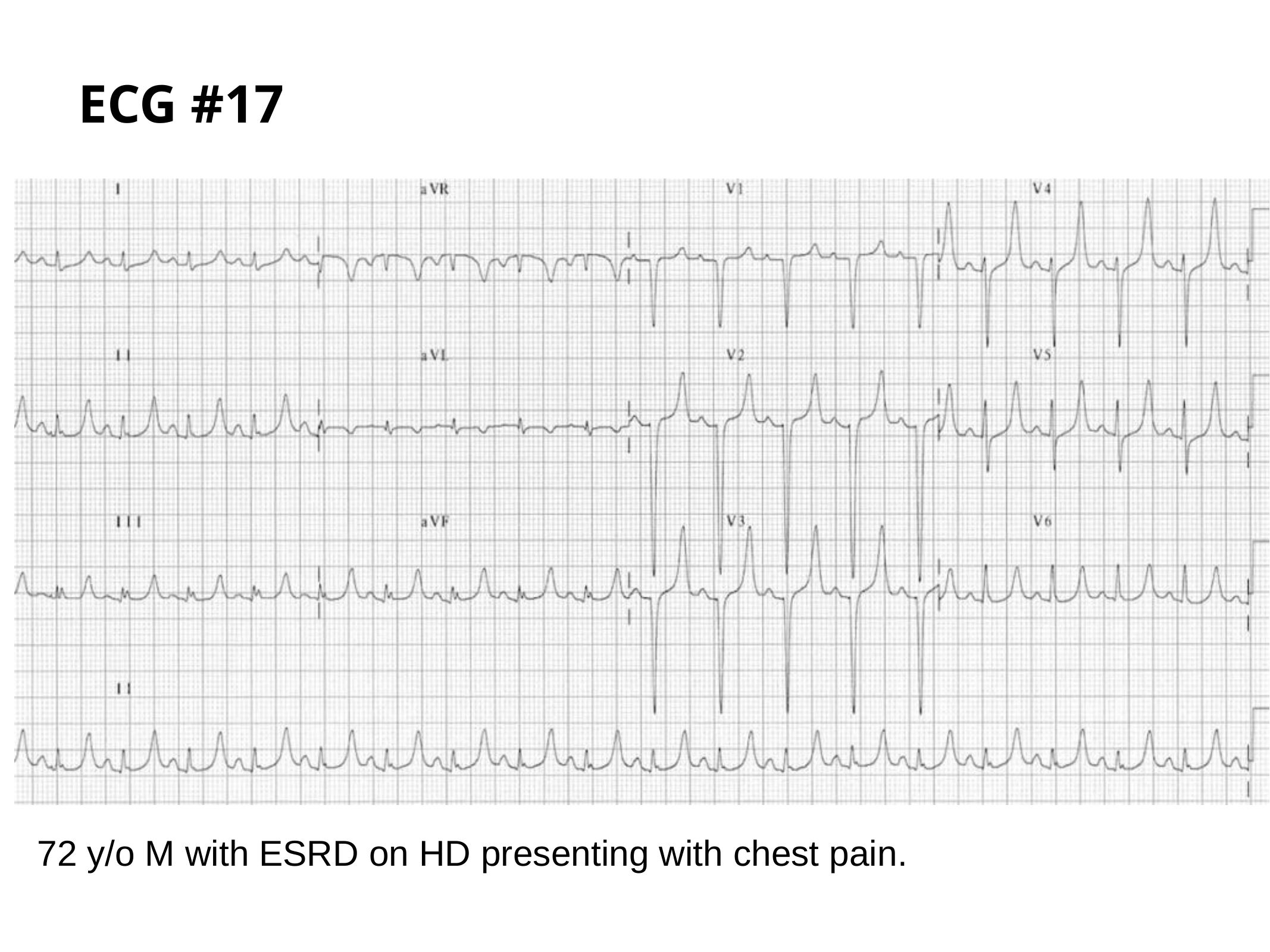

ECG #17
72 y/o M with ESRD on HD presenting with chest pain.

## Slide 54
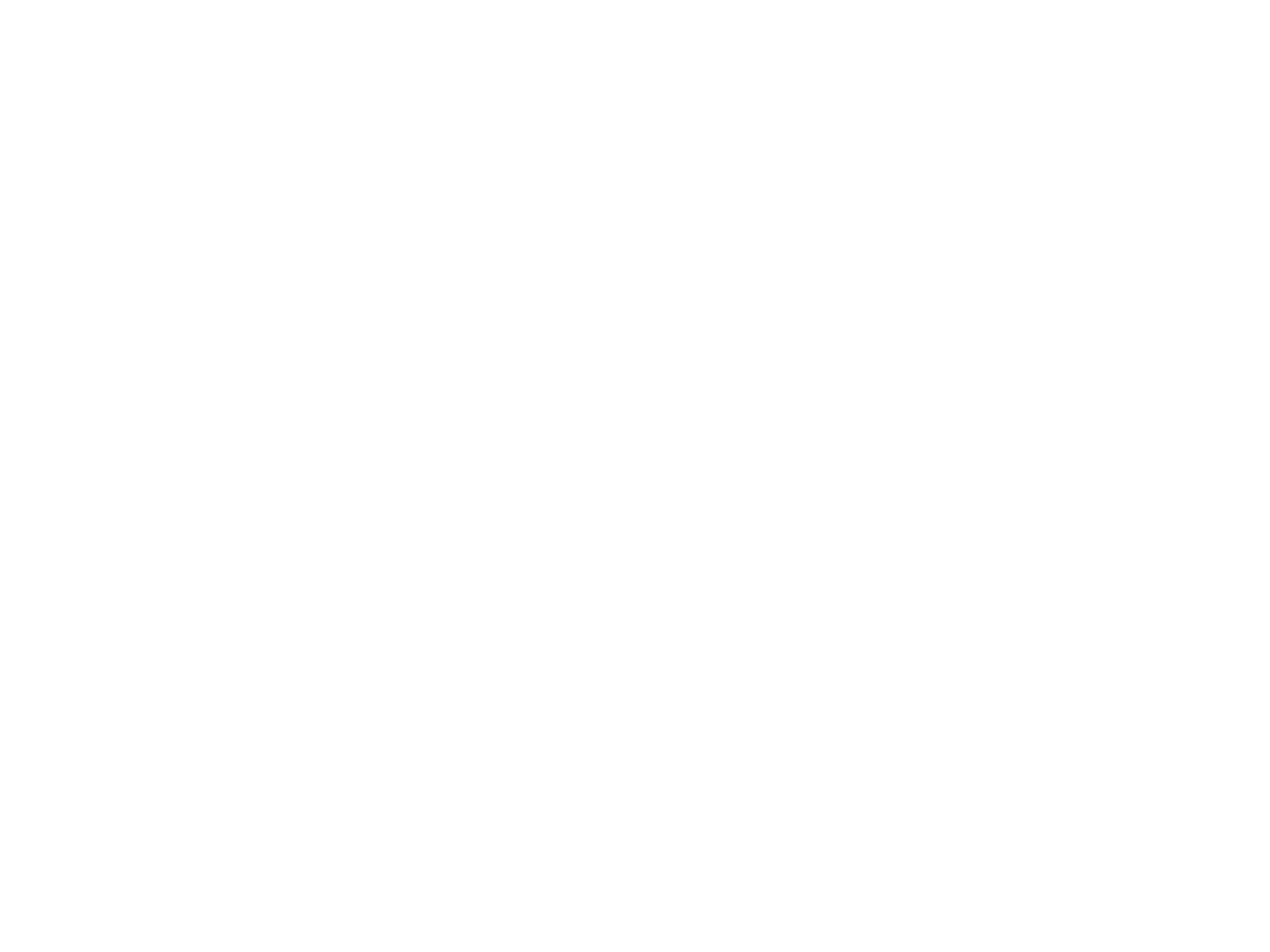

## Slide 55
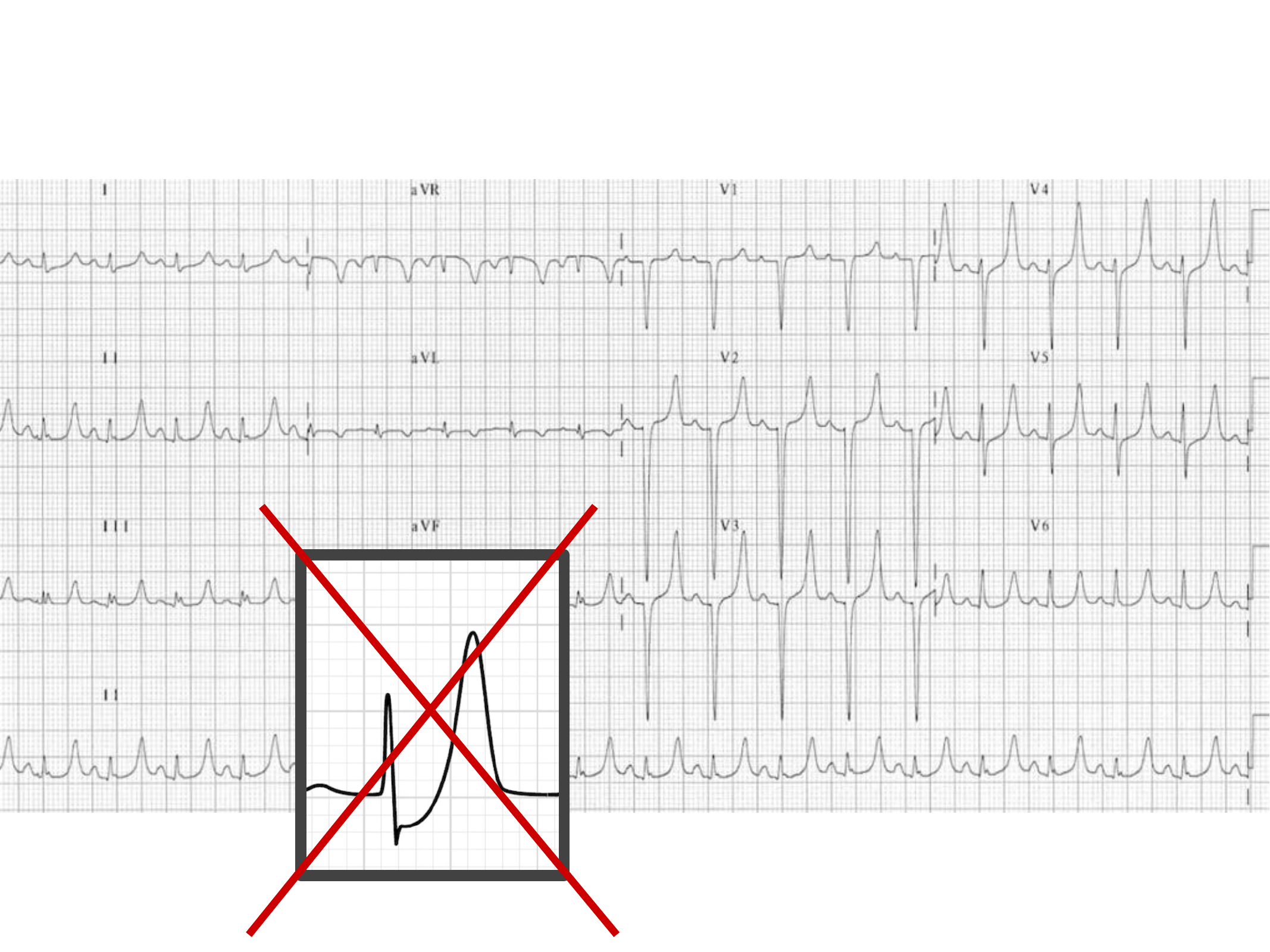

## Slide 56
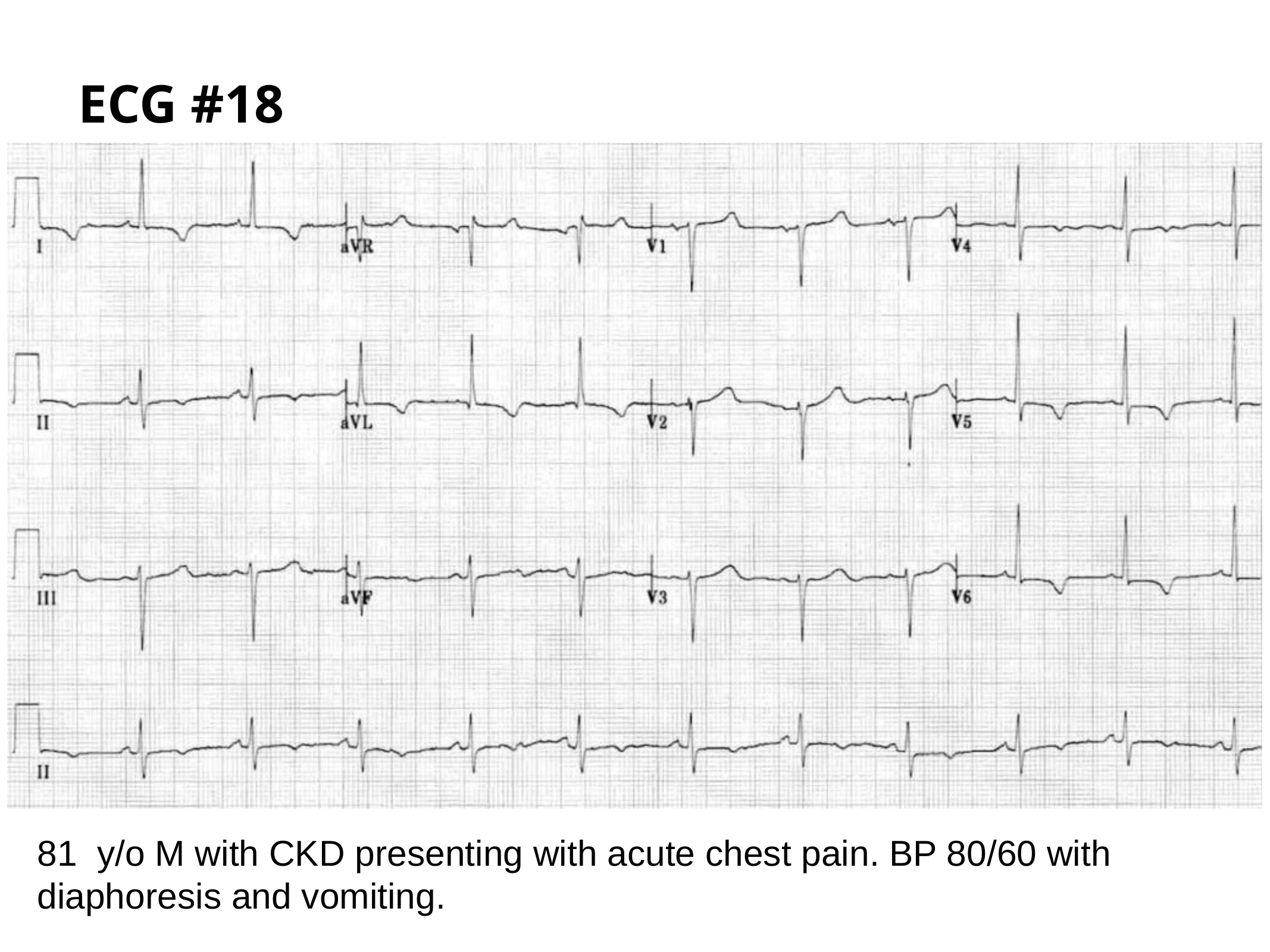

ECG #18
81 y/o M with CKD presenting with acute chest pain. BP 80/60 with diaphoresis and vomiting.

## Slide 57
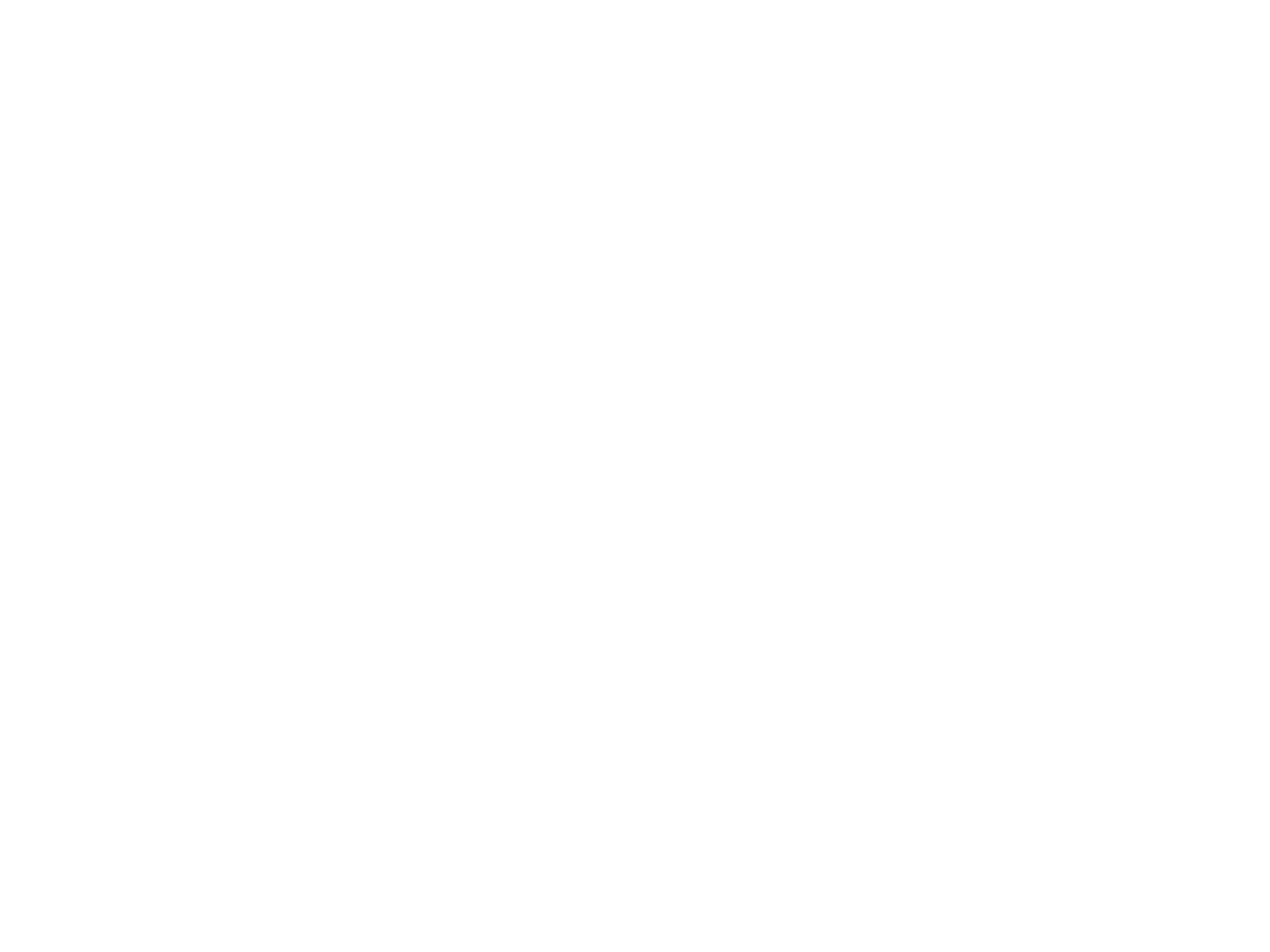

## Slide 58
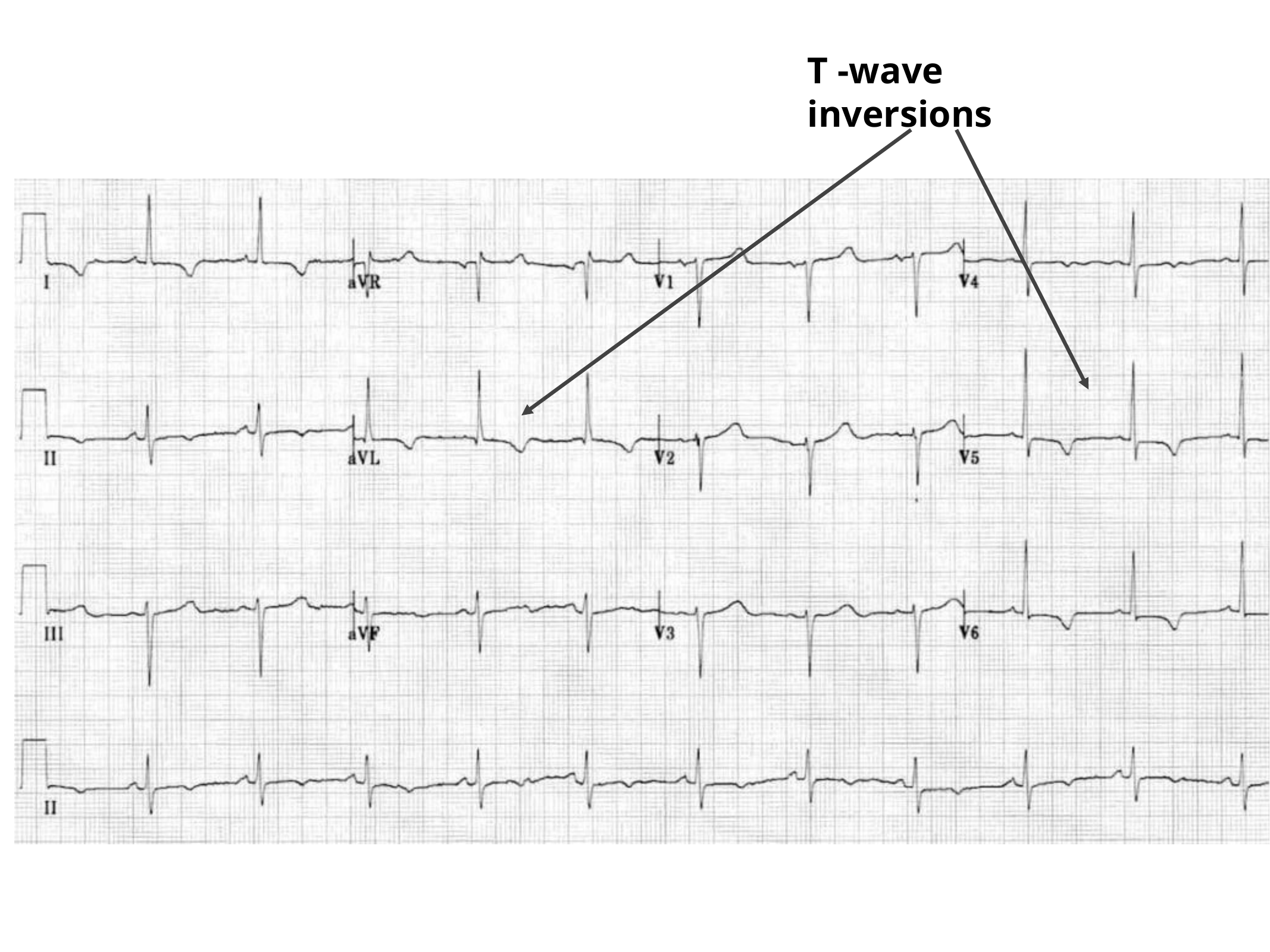

T -wave inversions

## Slide 59
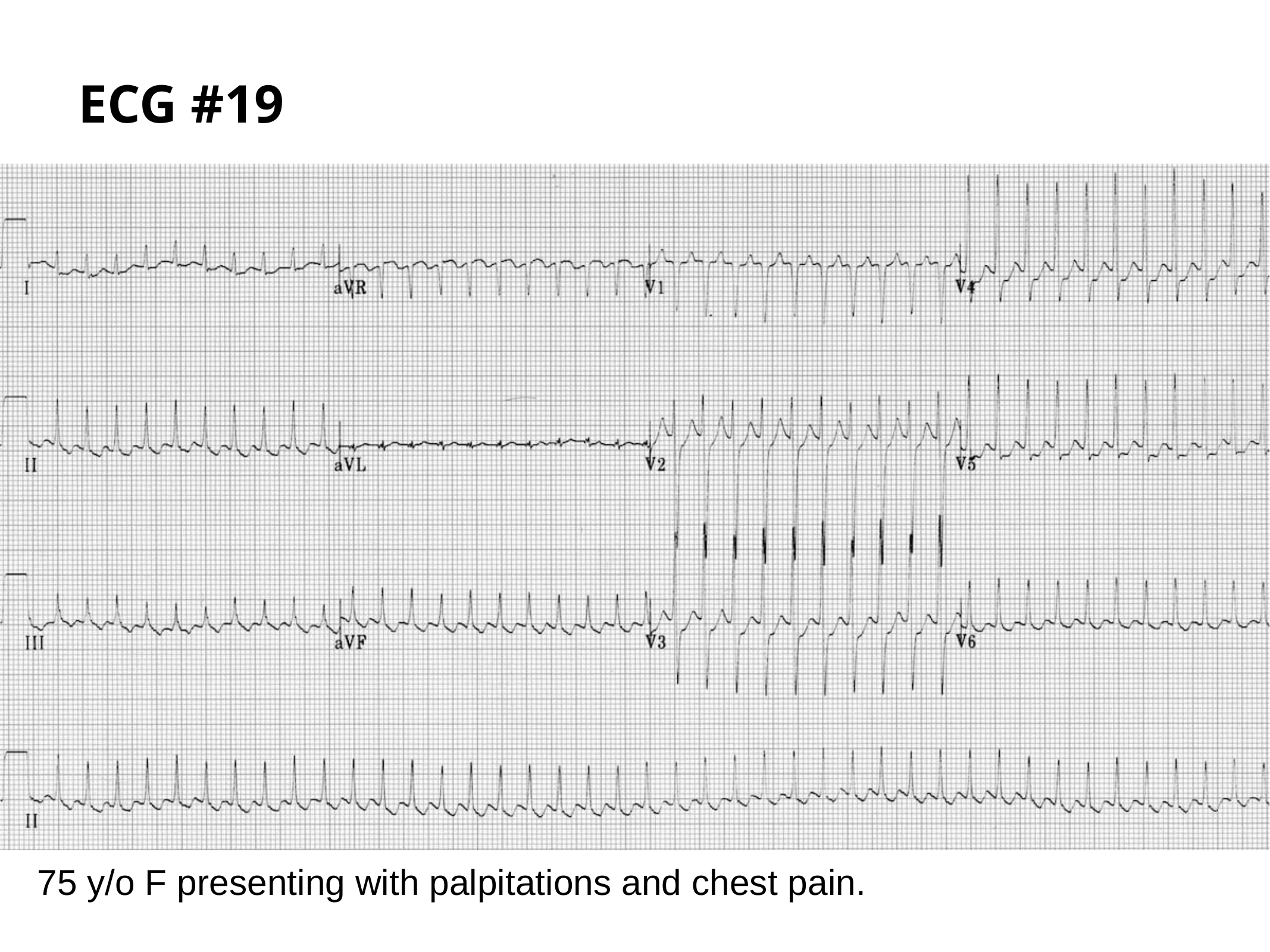

ECG #19
75 y/o F presenting with palpitations and chest pain.

## Slide 60
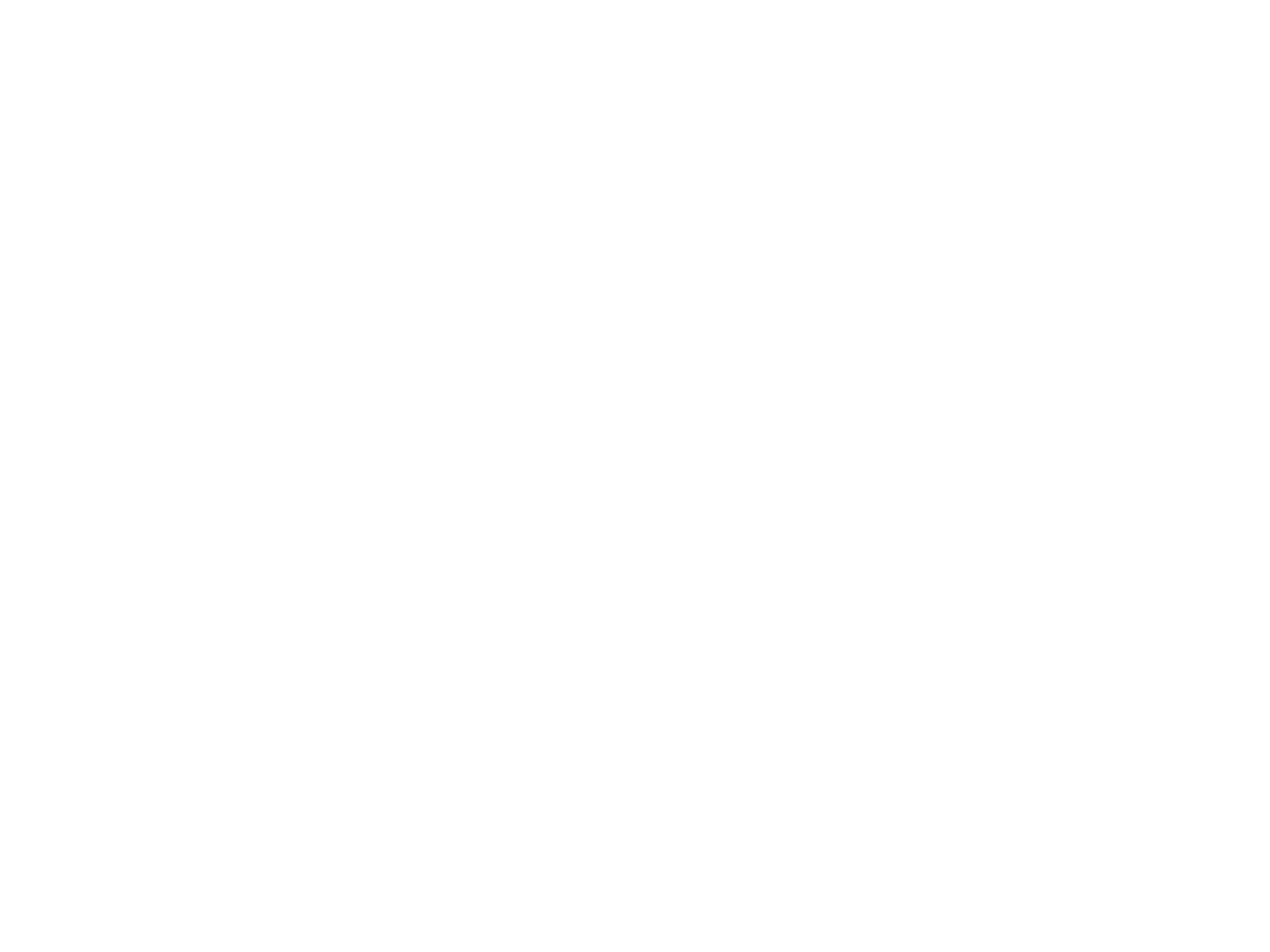

## Slide 61
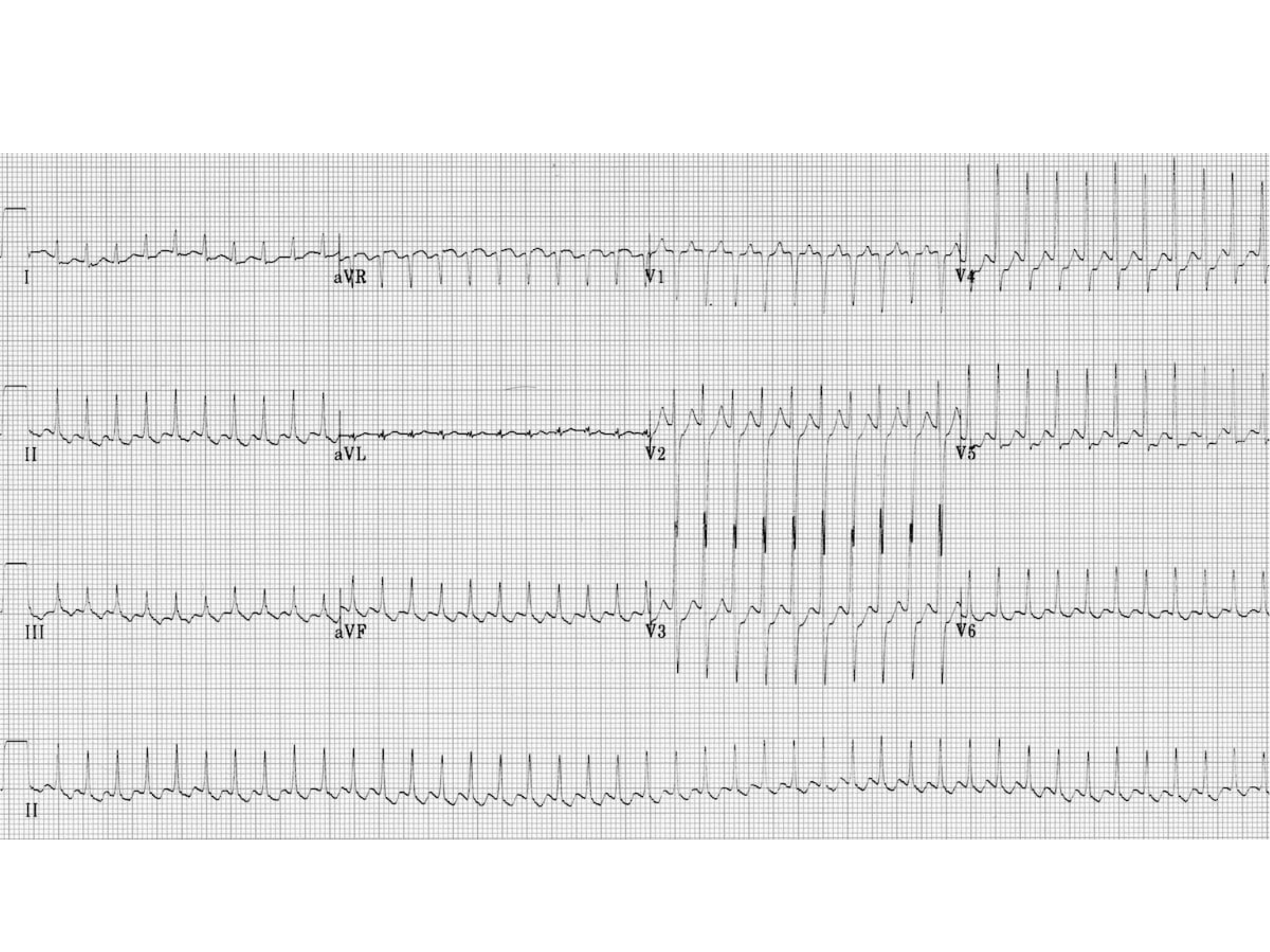

## Slide 62
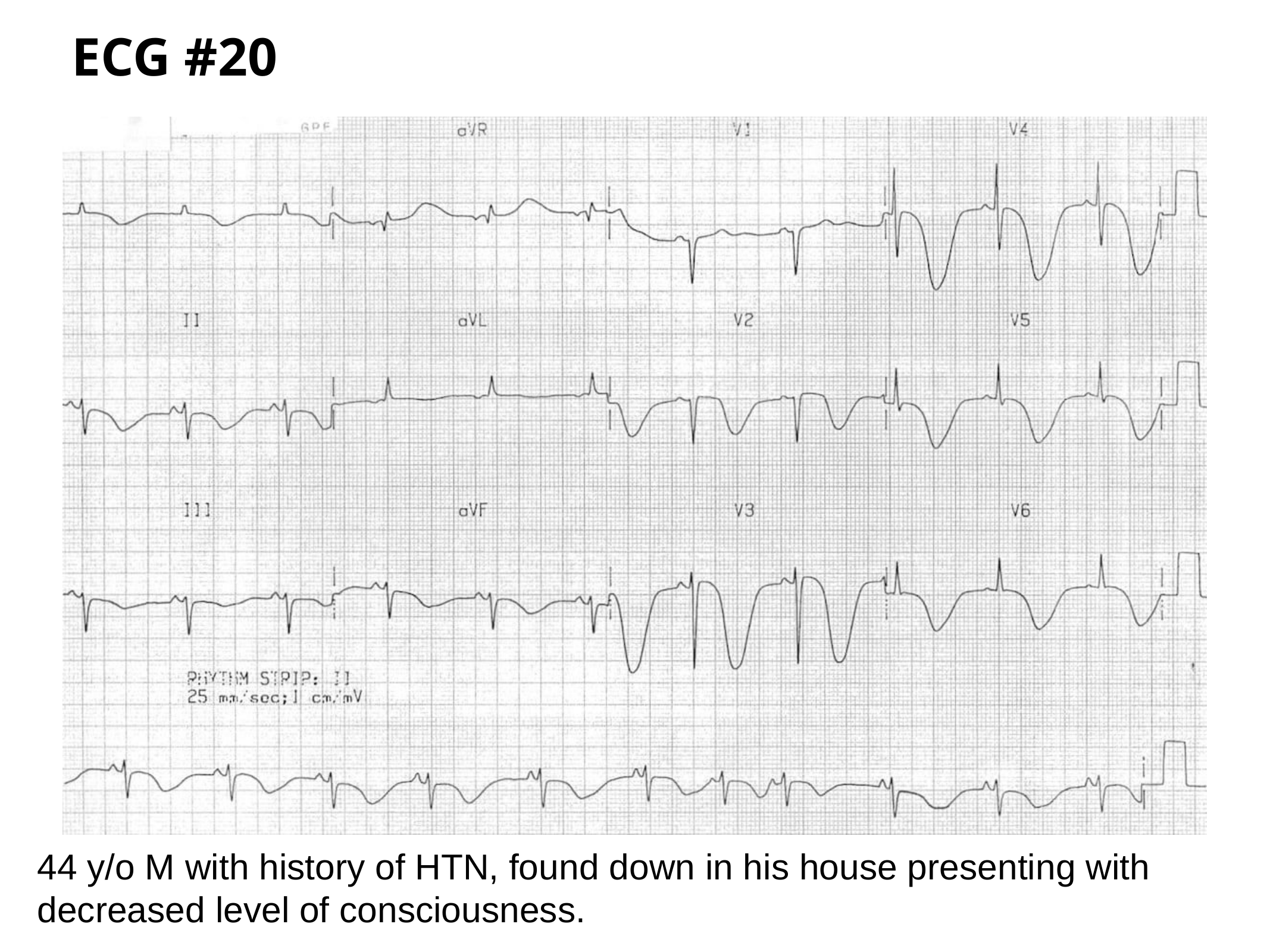

ECG #20
44 y/o M with history of HTN, found down in his house presenting with decreased level of consciousness.

## Slide 63
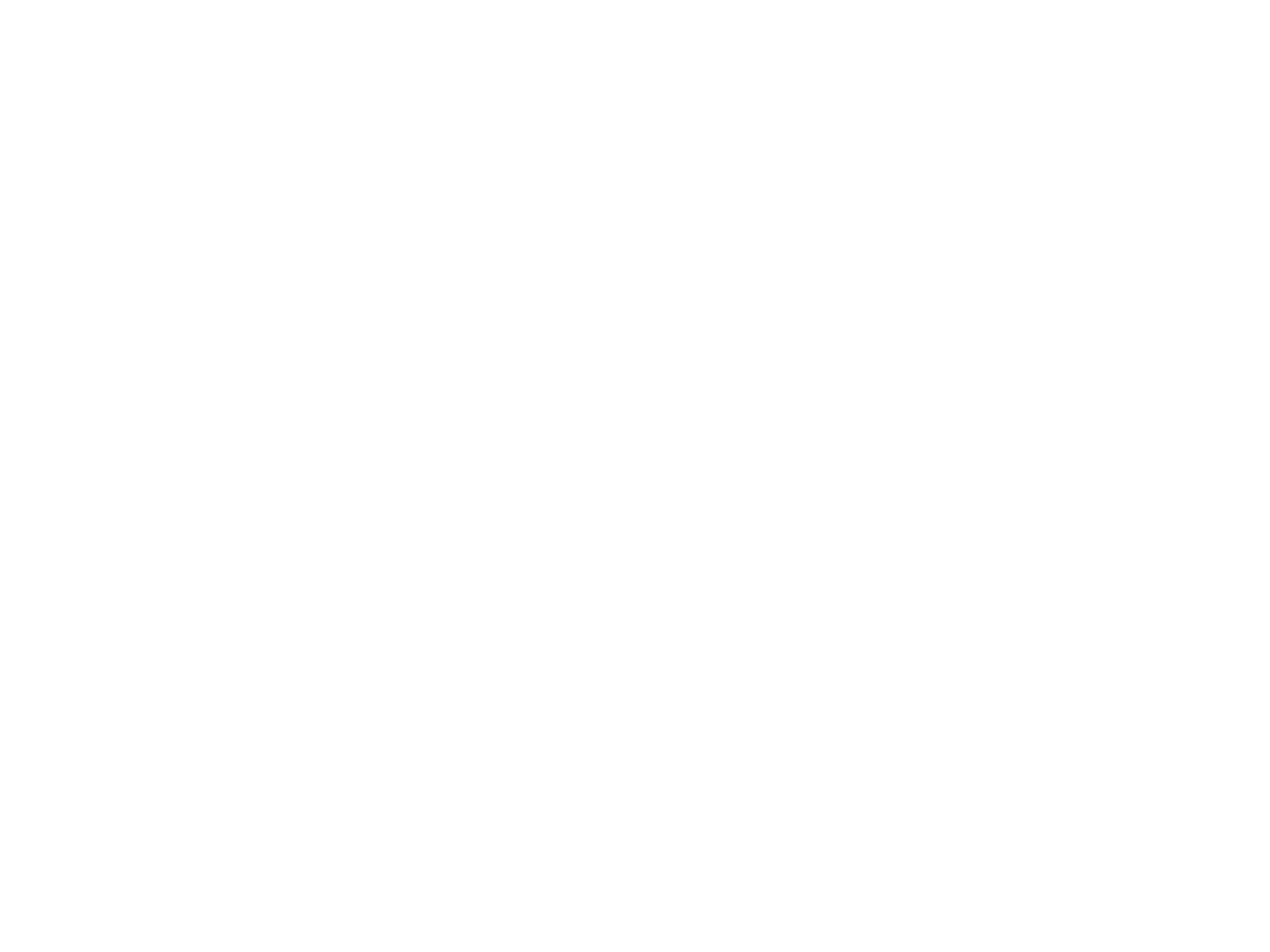

## Slide 64
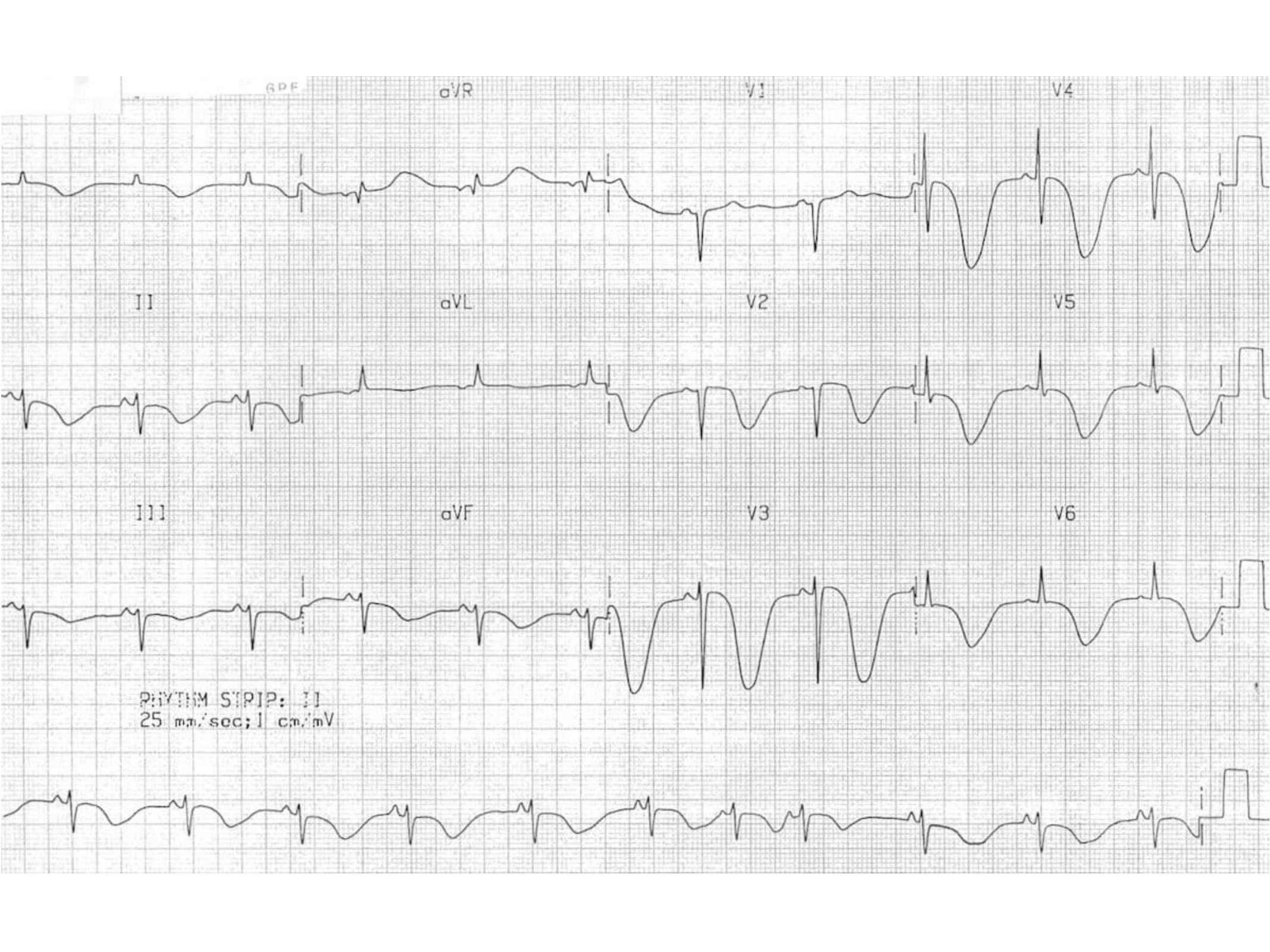

## Slide 65
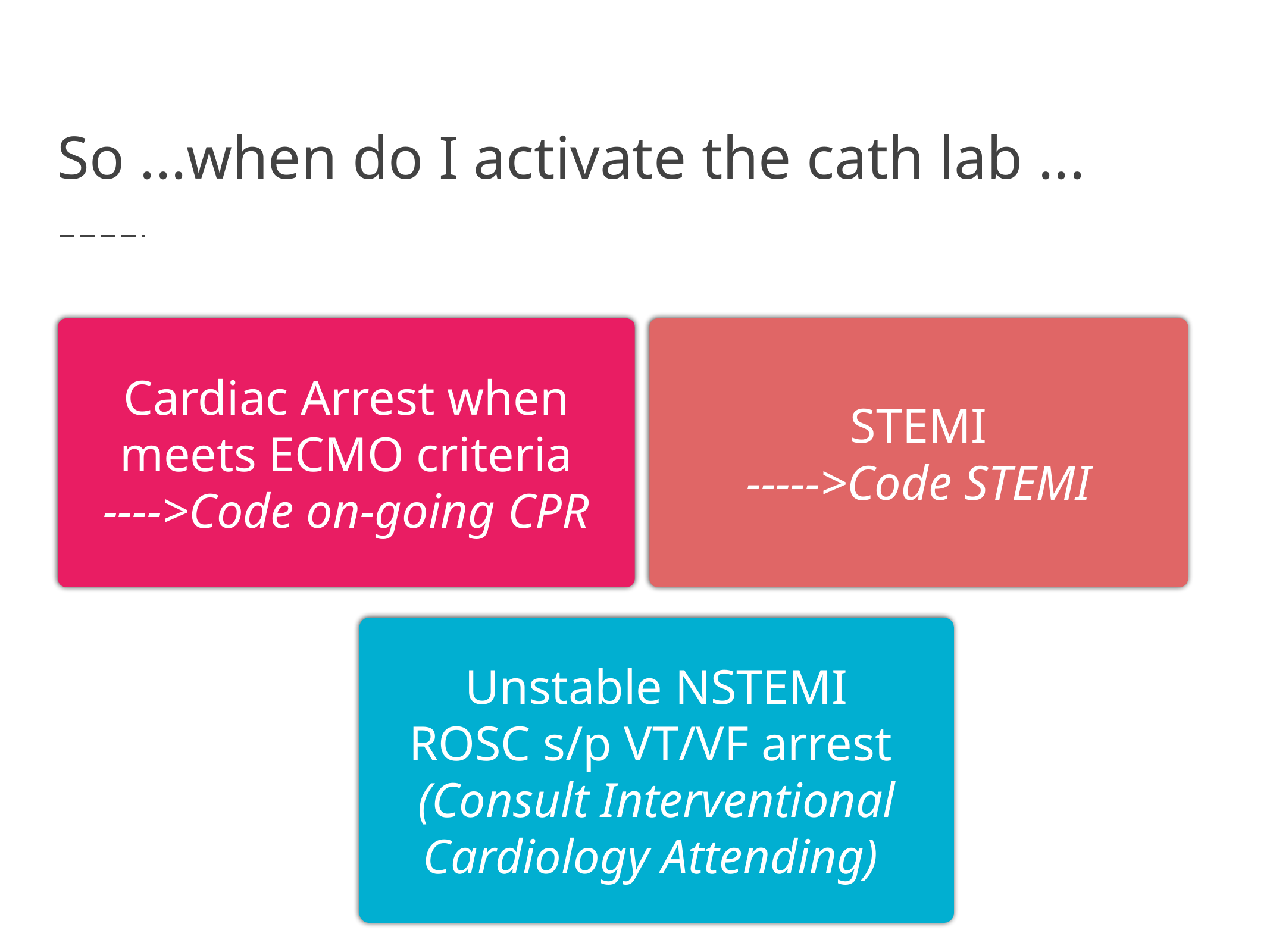

# So ...when do I activate the cath lab ...
Cardiac Arrest when meets ECMO criteria
---->Code on-going CPR
STEMI
----->Code STEMI
Unstable NSTEMI
ROSC s/p VT/VF arrest
(Consult Interventional Cardiology Attending)

## Slide 66
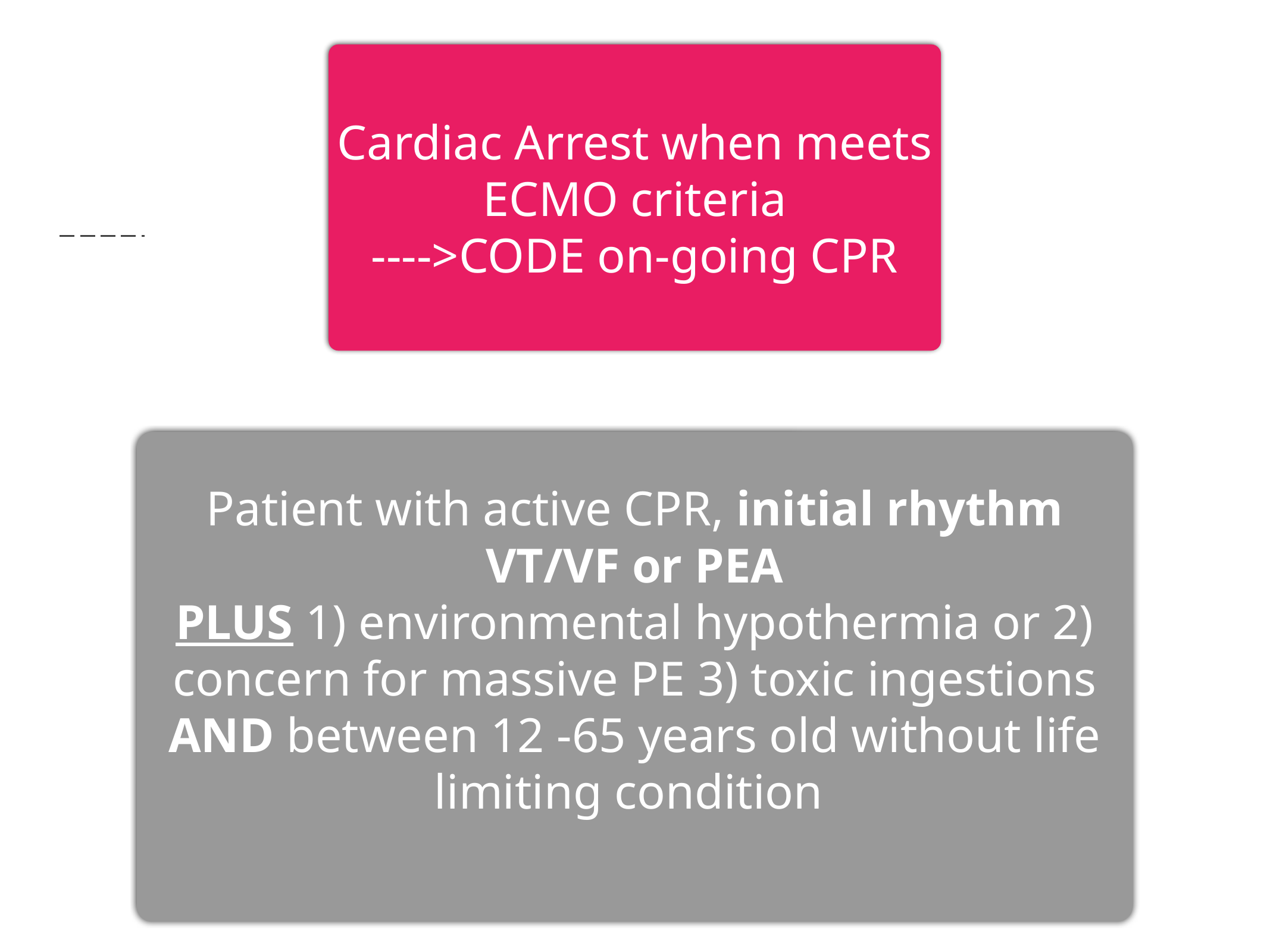

Cardiac Arrest when meets ECMO criteria
---->CODE on-going CPR
Patient with active CPR, initial rhythm VT/VF or PEA
PLUS 1) environmental hypothermia or 2) concern for massive PE 3) toxic ingestions
AND between 12 -65 years old without life limiting condition
No
No
Yes
Yes

## Slide 67
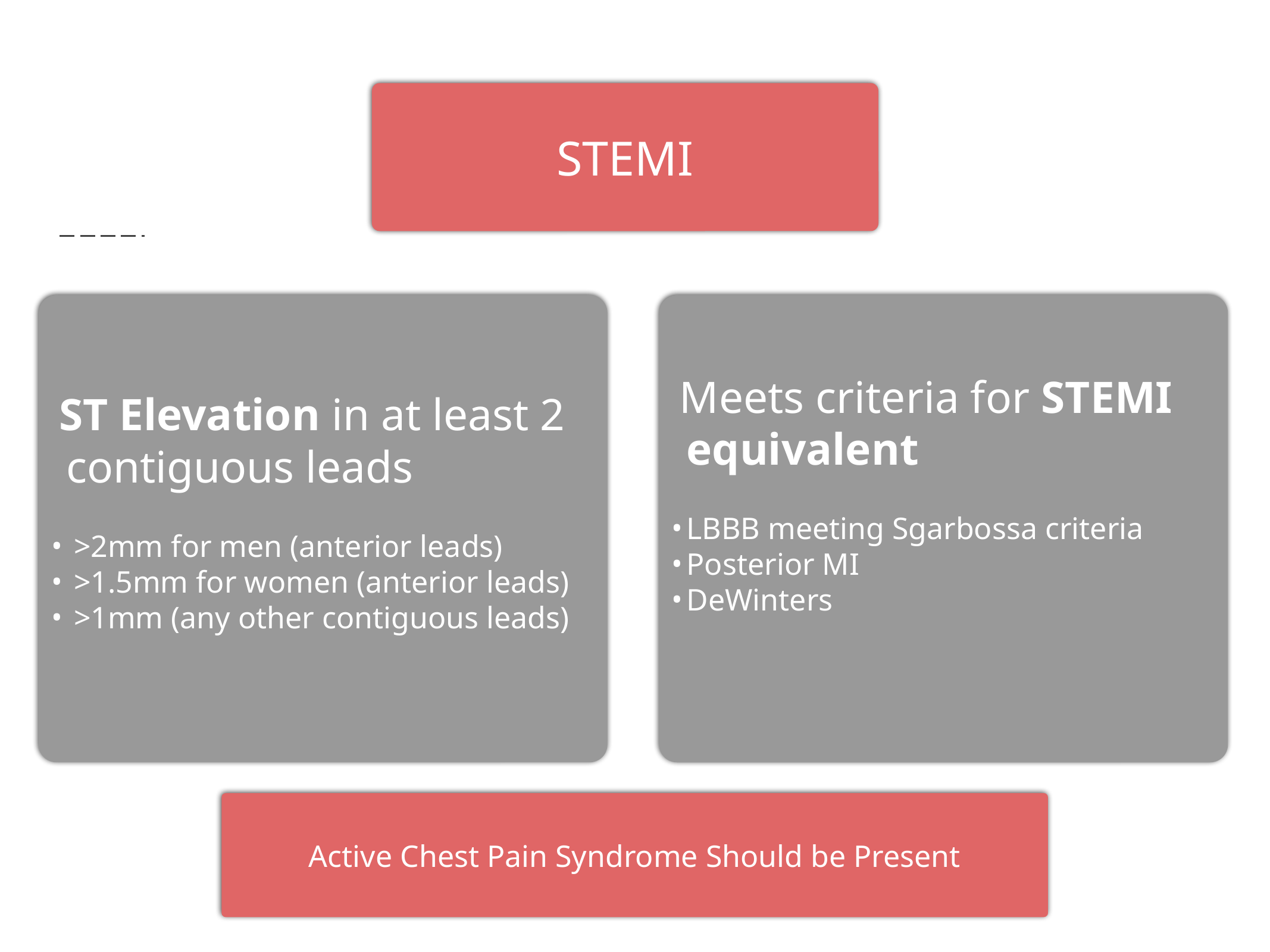

STEMI
ST Elevation in at least 2 contiguous leads
 >2mm for men (anterior leads)
 >1.5mm for women (anterior leads)
 >1mm (any other contiguous leads)
Meets criteria for STEMI equivalent
LBBB meeting Sgarbossa criteria
Posterior MI
DeWinters
Active Chest Pain Syndrome Should be Present

## Slide 68
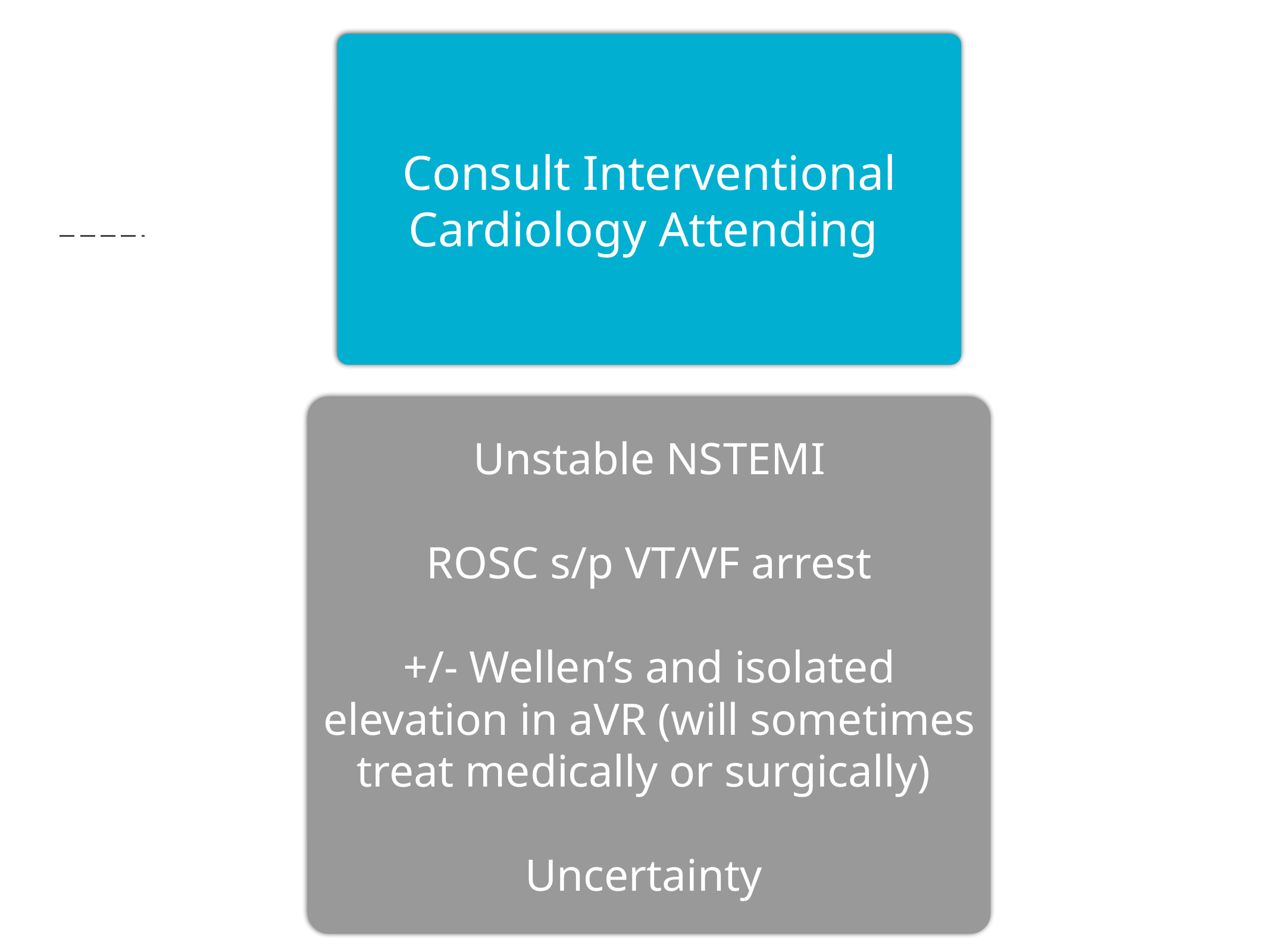

Consult Interventional Cardiology Attending
Unstable NSTEMI
ROSC s/p VT/VF arrest
+/- Wellen’s and isolated elevation in aVR (will sometimes treat medically or surgically)
Uncertainty

## Slide 69
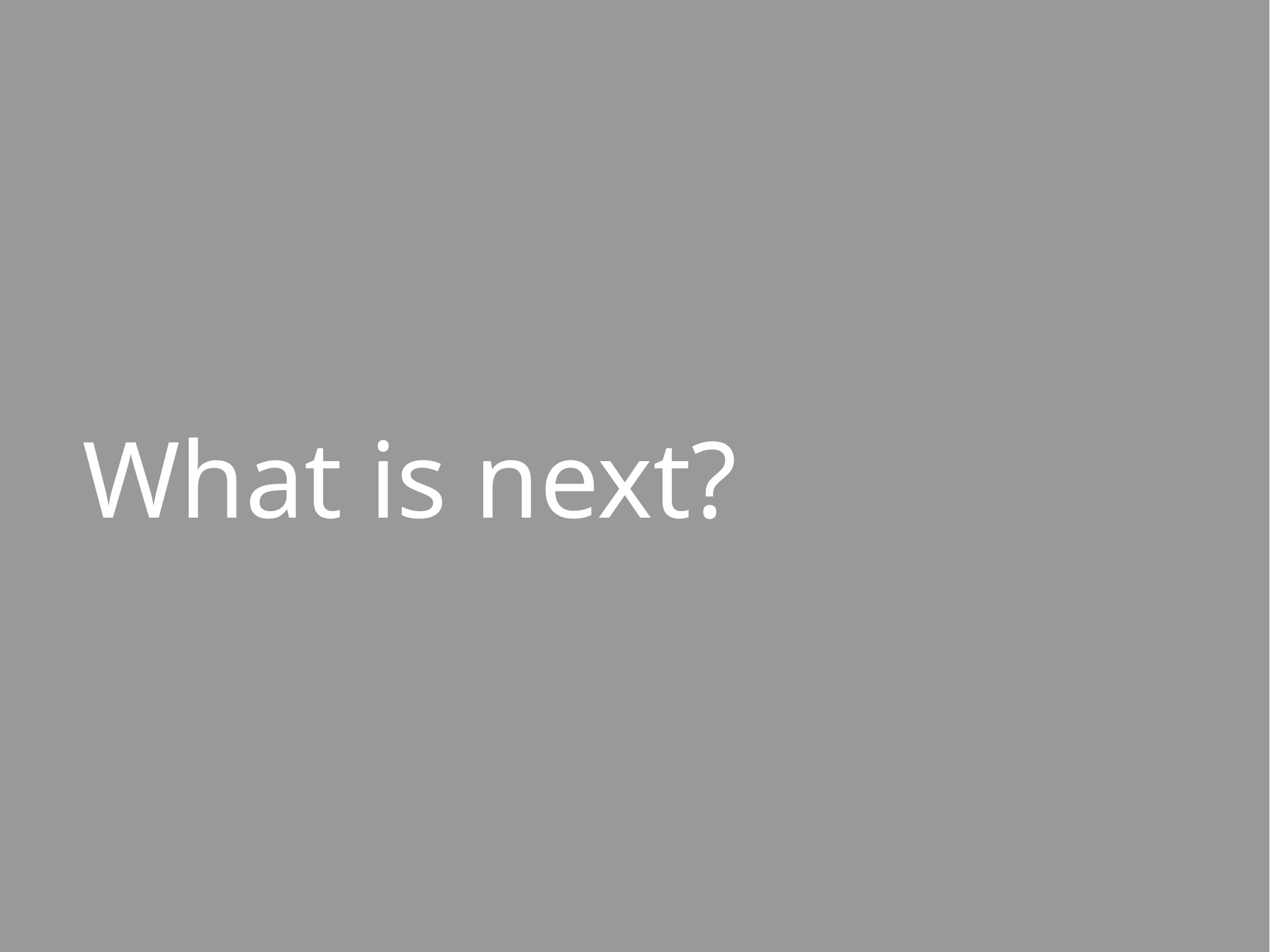

# What is next?
