# Supplementary material for: Ischemic ECG Pattern Recognition to Facilitate Interpretation While Task Switching: A Parallel Curriculum
Source: MedEdPORTAL. 2021 Sep 7;17:11182. doi: 10.15766/mep_2374-8265.11182 (PMC8421424; doi:10.15766/mep_2374-8265.11182)
Supplement: Supplementary file 1 — Introduction Lecture.pptxKnowledge Pretest Answer Sheet.docxECG Handout.docxECG Handout Answers.docxReview Lecture.pptxPresurvey of Confidence.docxPostsurvey of Confidence.docxCourse Evaluation.docxDelayed Knowledge Posttest.docx [file mep_2374-8265.11182-s001.zip › B. Knowledge Pretest Answer Sheet.docx]

**APPENDIX B: Pre-Test Answer Sheet**

Thank you for your participation in today’s class. Participation in this test is optional. Your responses will be used to improve teaching for trainees.

In order to link your responses to this survey with future surveys, please provide your initials and the last four digits of your cell phone (i.e., CS-3227)

**Initials:____________________ Last 4 digits of cell phone: _____________________**

**For each ECG displayed on the PowerPoint for ten seconds. You will then decide if you would 1) activate the cath lab, 2) not activate the cath lab, or 3) call cardiology (for possible cath lab activation). Place an “X” indicating your answer for each ECG.**

| **ECG Number** | **Activate cath lab** | **Do not activate the cath lab** | **Call cardiology** |
| --- | --- | --- | --- |
| **1** |  |  |  |
| **2** |  |  |  |
| **3** |  |  |  |
| **4** |  |  |  |
| **5** |  |  |  |
| **6** |  |  |  |
| **7** |  |  |  |
| **8** |  |  |  |
| **9** |  |  |  |
| **10** |  |  |  |
| **11** |  |  |  |
| **12** |  |  |  |
| **13** |  |  |  |
| **14** |  |  |  |
| **15** |  |  |  |
| **16** |  |  |  |
| **17** |  |  |  |
| **18** |  |  |  |
| **19** |  |  |  |
| **20** |  |  |  |
