# Supplementary material for: Ischemic ECG Pattern Recognition to Facilitate Interpretation While Task Switching: A Parallel Curriculum
Source: MedEdPORTAL. 2021 Sep 7;17:11182. doi: 10.15766/mep_2374-8265.11182 (PMC8421424; doi:10.15766/mep_2374-8265.11182)
Supplement: Supplementary file 1 — Introduction Lecture.pptxKnowledge Pretest Answer Sheet.docxECG Handout.docxECG Handout Answers.docxReview Lecture.pptxPresurvey of Confidence.docxPostsurvey of Confidence.docxCourse Evaluation.docxDelayed Knowledge Posttest.docx [file mep_2374-8265.11182-s001.zip › E. Review Lecture.pptx]

## Slide 1
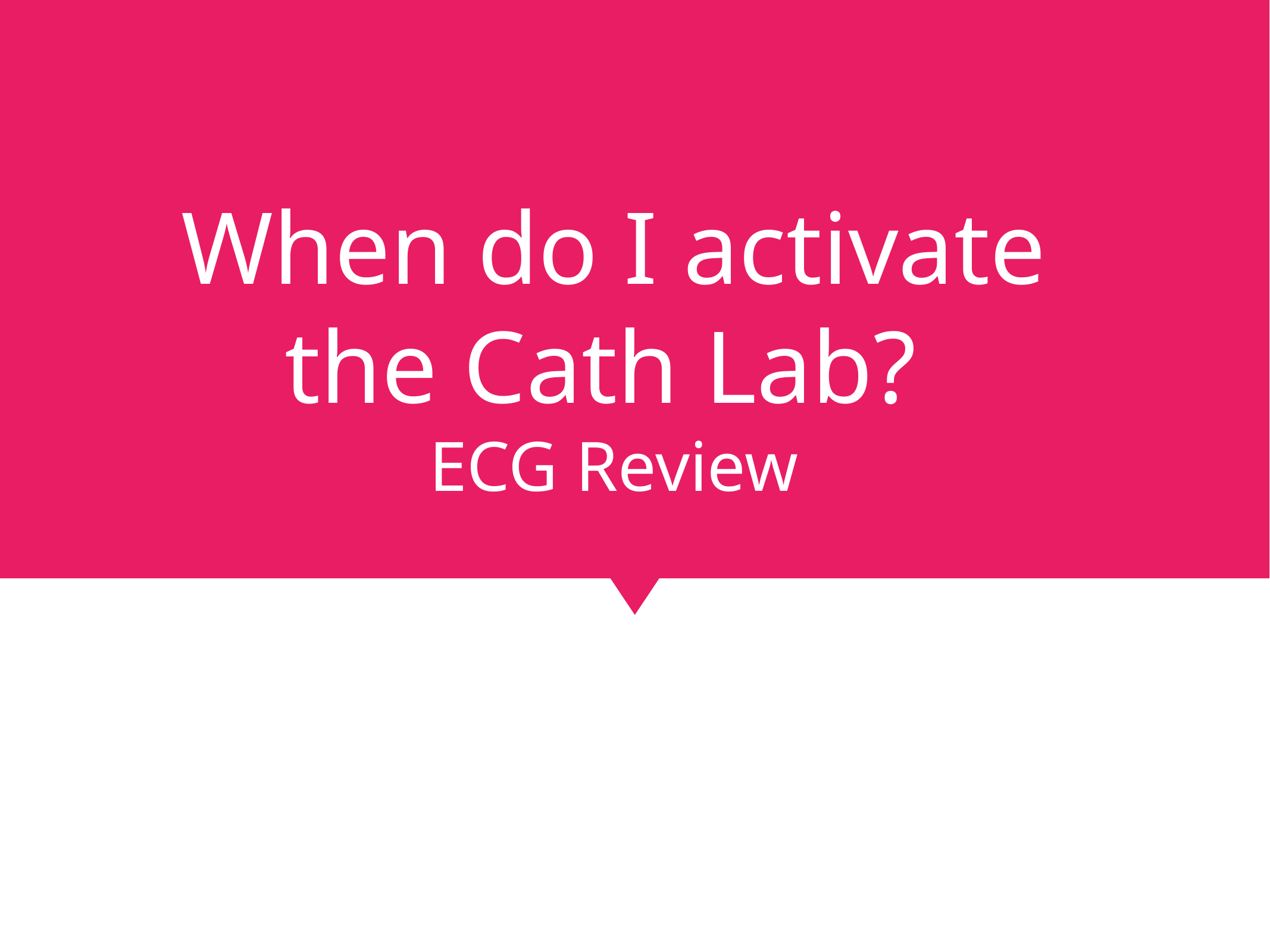

# When do I activate the Cath Lab?
ECG Review

## Slide 2
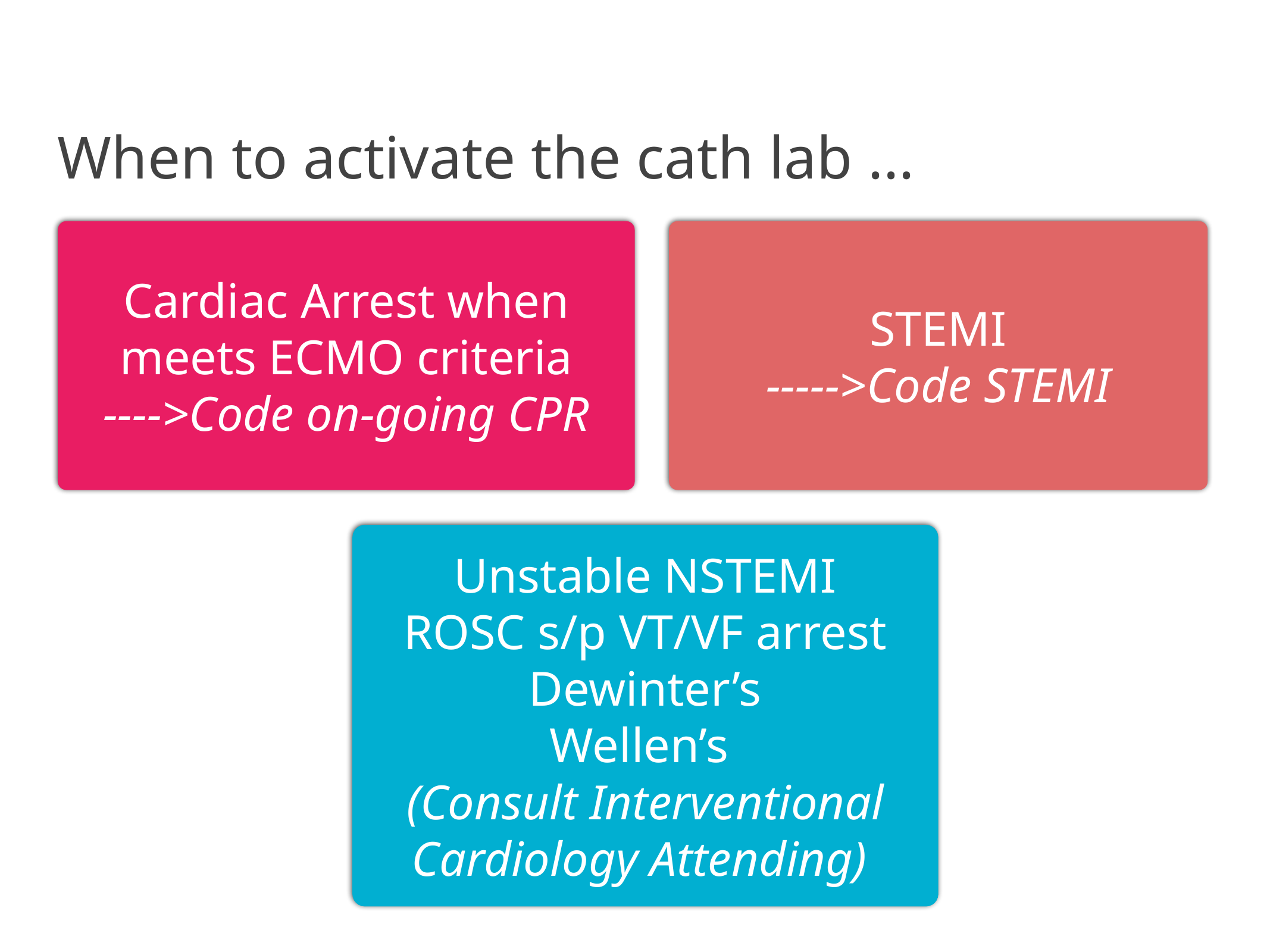

# When to activate the cath lab ...
Cardiac Arrest when meets ECMO criteria
---->Code on-going CPR
STEMI
----->Code STEMI
Unstable NSTEMI
ROSC s/p VT/VF arrest
Dewinter’s
Wellen’s
(Consult Interventional Cardiology Attending)

## Slide 3
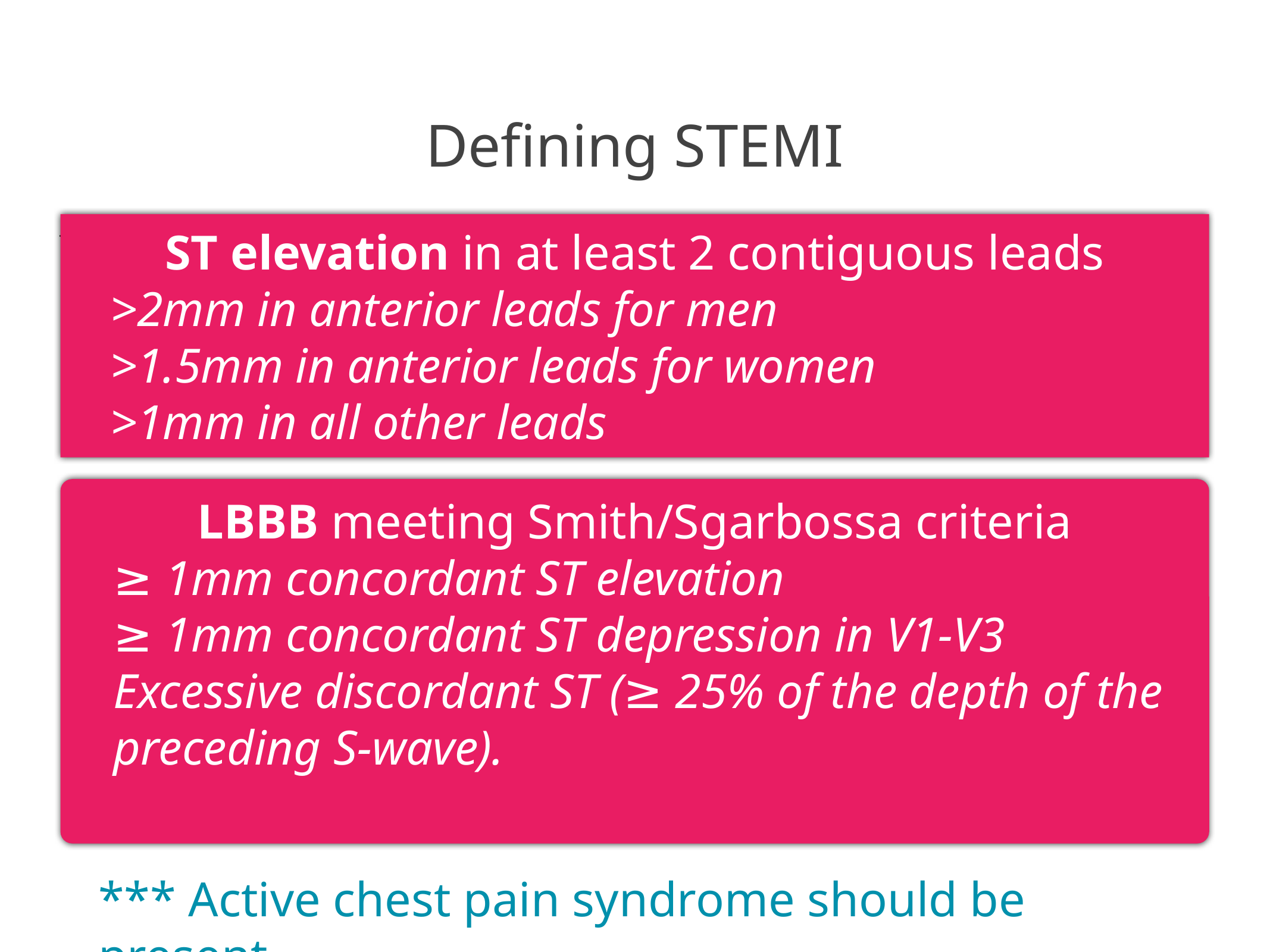

# Defining STEMI
ST elevation in at least 2 contiguous leads
>2mm in anterior leads for men
>1.5mm in anterior leads for women
>1mm in all other leads
LBBB meeting Smith/Sgarbossa criteria
≥ 1mm concordant ST elevation
≥ 1mm concordant ST depression in V1-V3
Excessive discordant ST (≥ 25% of the depth of the preceding S-wave).
*** Active chest pain syndrome should be present

## Slide 4
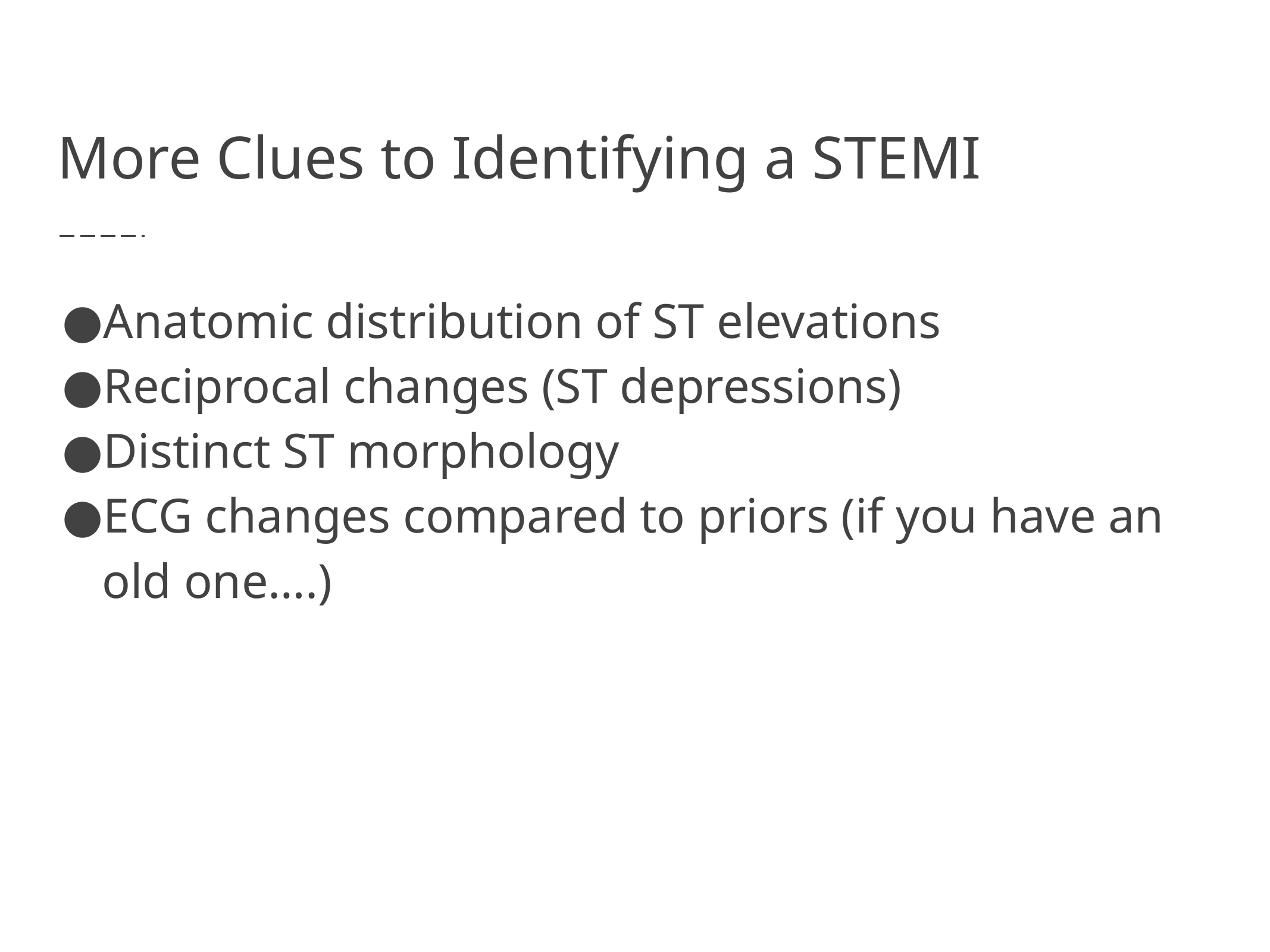

# More Clues to Identifying a STEMI
Anatomic distribution of ST elevations
Reciprocal changes (ST depressions)
Distinct ST morphology
ECG changes compared to priors (if you have an old one….)

## Slide 5
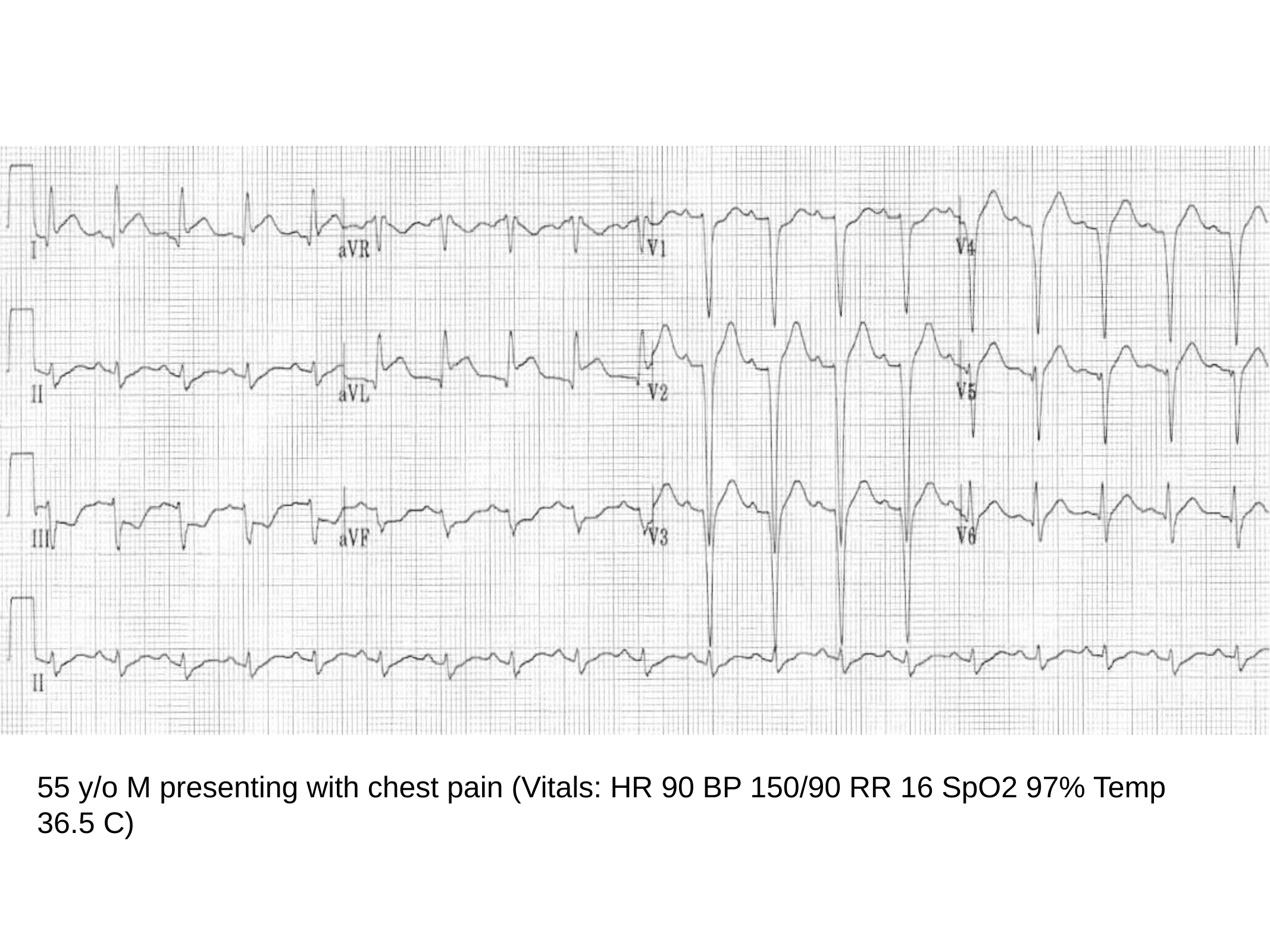

55 y/o M presenting with chest pain (Vitals: HR 90 BP 150/90 RR 16 SpO2 97% Temp 36.5 C)

## Slide 6
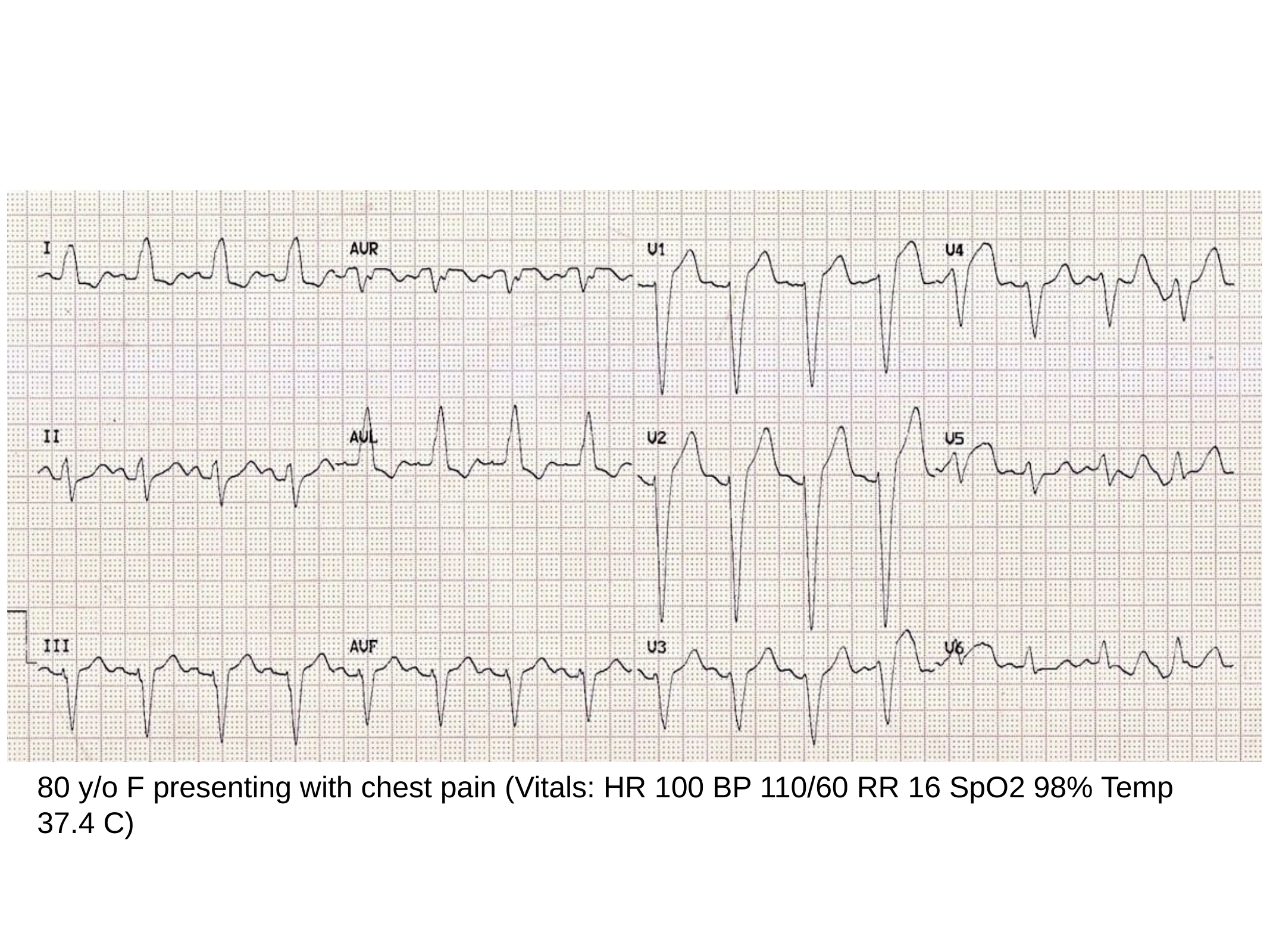

80 y/o F presenting with chest pain (Vitals: HR 100 BP 110/60 RR 16 SpO2 98% Temp 37.4 C)

## Slide 7
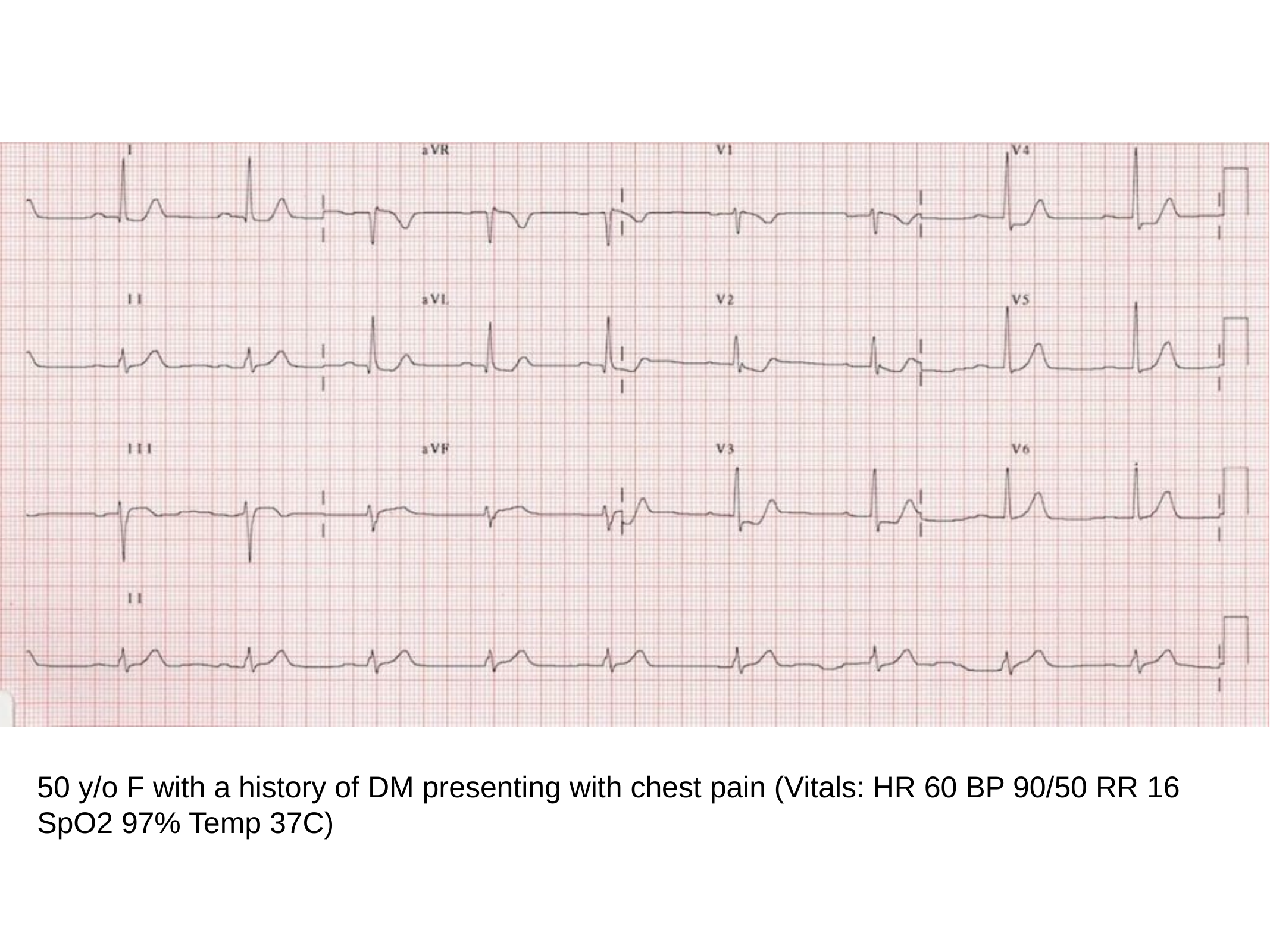

50 y/o F with a history of DM presenting with chest pain (Vitals: HR 60 BP 90/50 RR 16 SpO2 97% Temp 37C)

## Slide 8
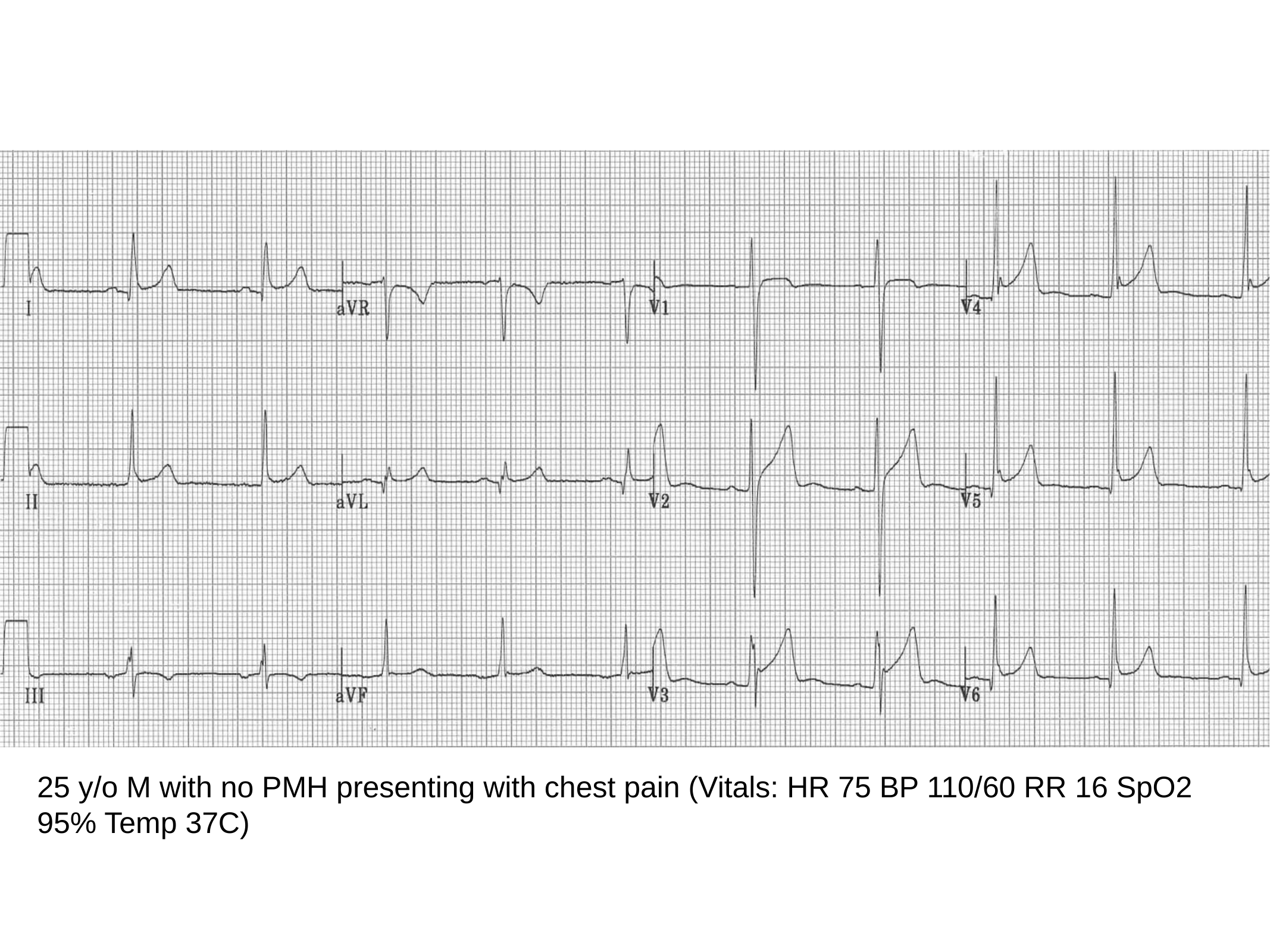

25 y/o M with no PMH presenting with chest pain (Vitals: HR 75 BP 110/60 RR 16 SpO2 95% Temp 37C)

## Slide 9
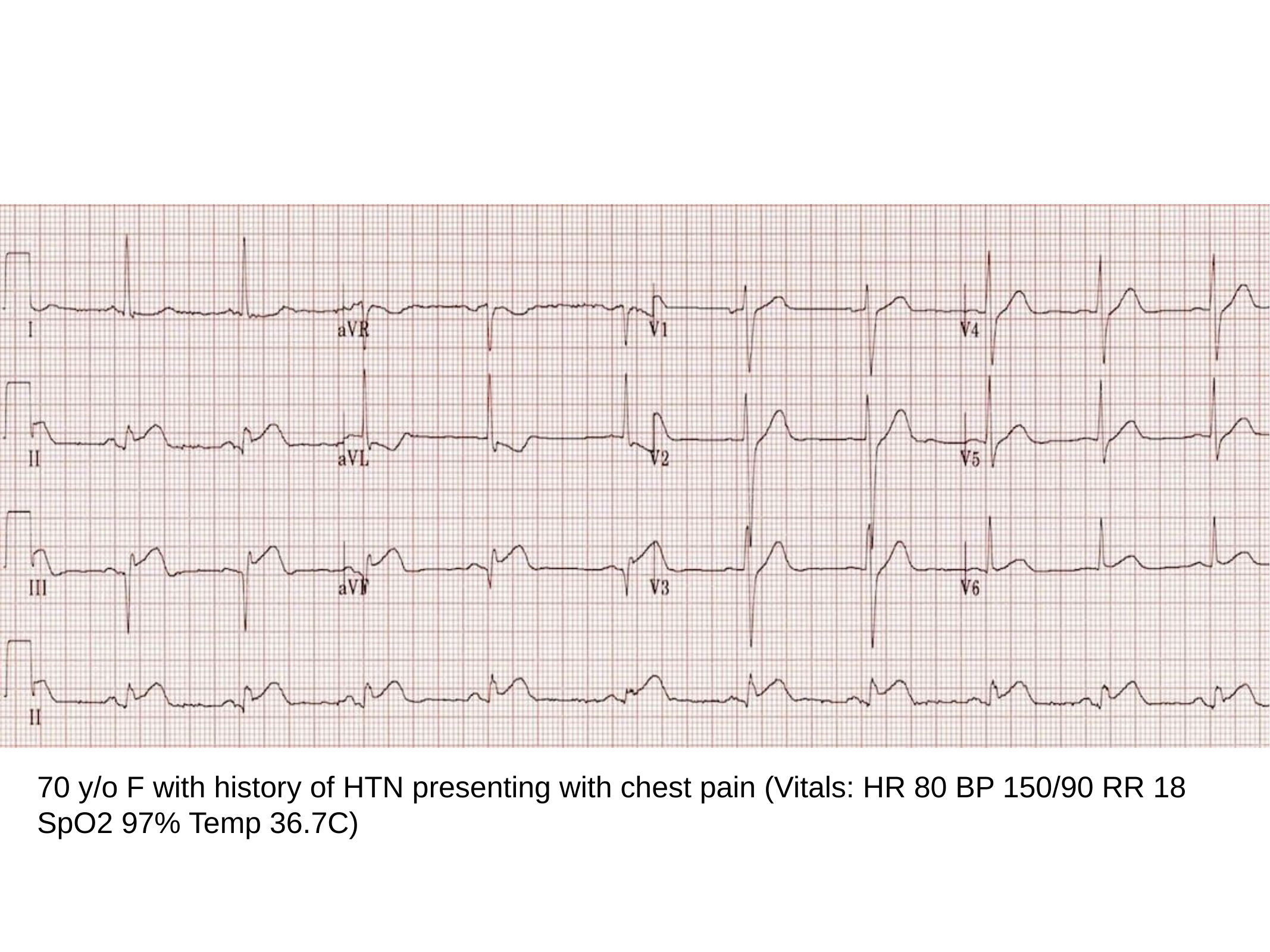

70 y/o F with history of HTN presenting with chest pain (Vitals: HR 80 BP 150/90 RR 18 SpO2 97% Temp 36.7C)

## Slide 10
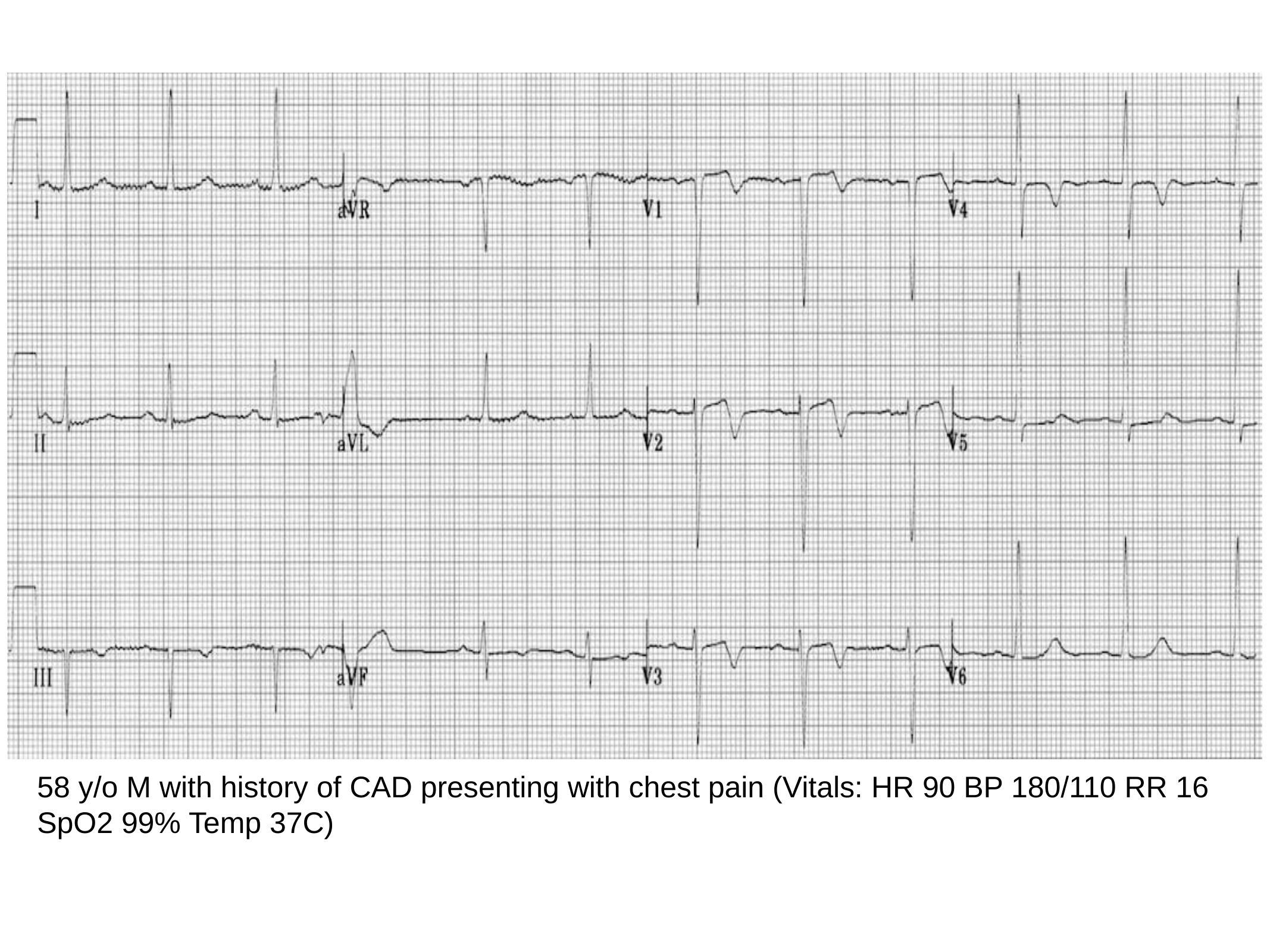

58 y/o M with history of CAD presenting with chest pain (Vitals: HR 90 BP 180/110 RR 16 SpO2 99% Temp 37C)

## Slide 11
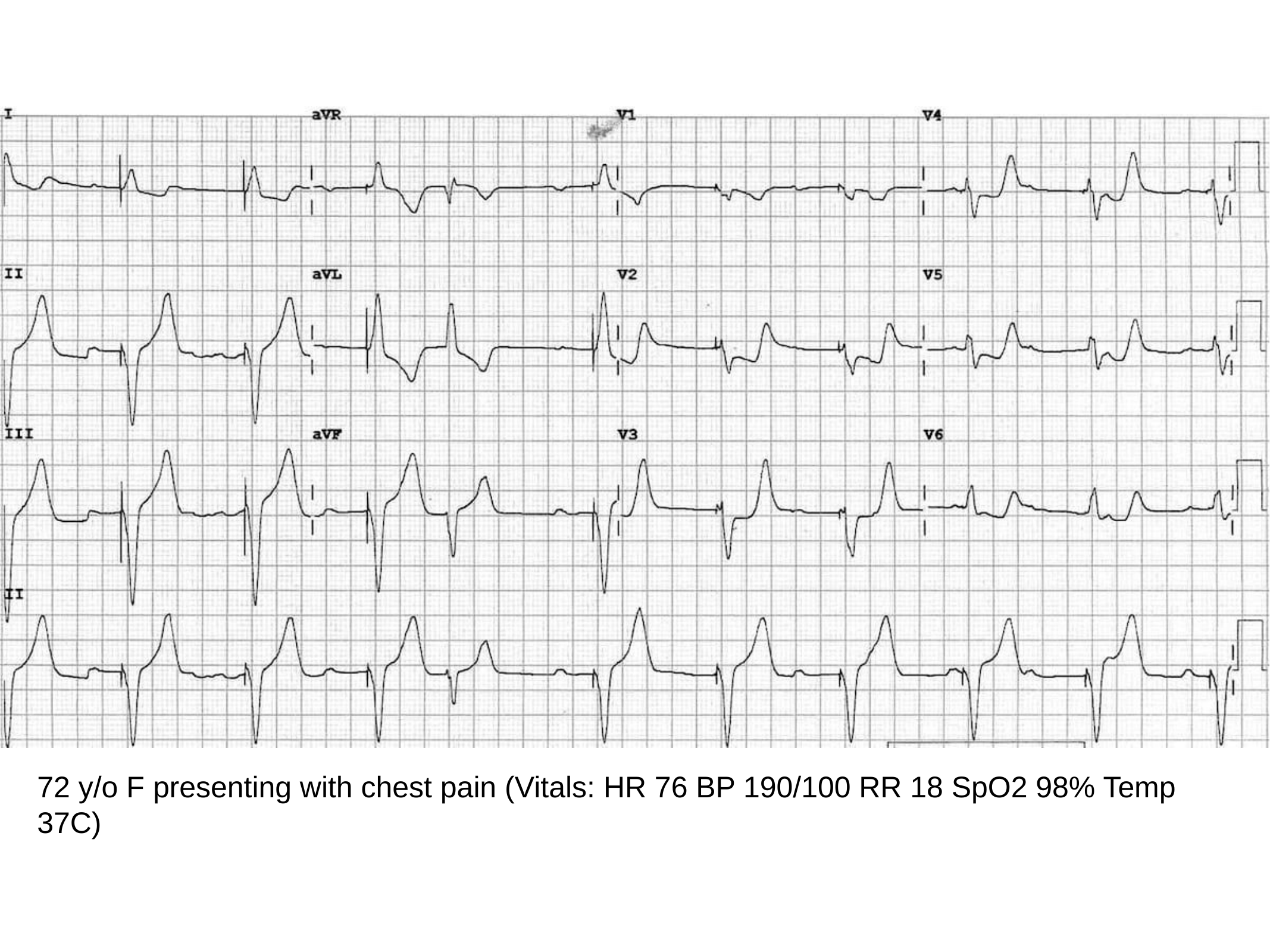

72 y/o F presenting with chest pain (Vitals: HR 76 BP 190/100 RR 18 SpO2 98% Temp 37C)

## Slide 12
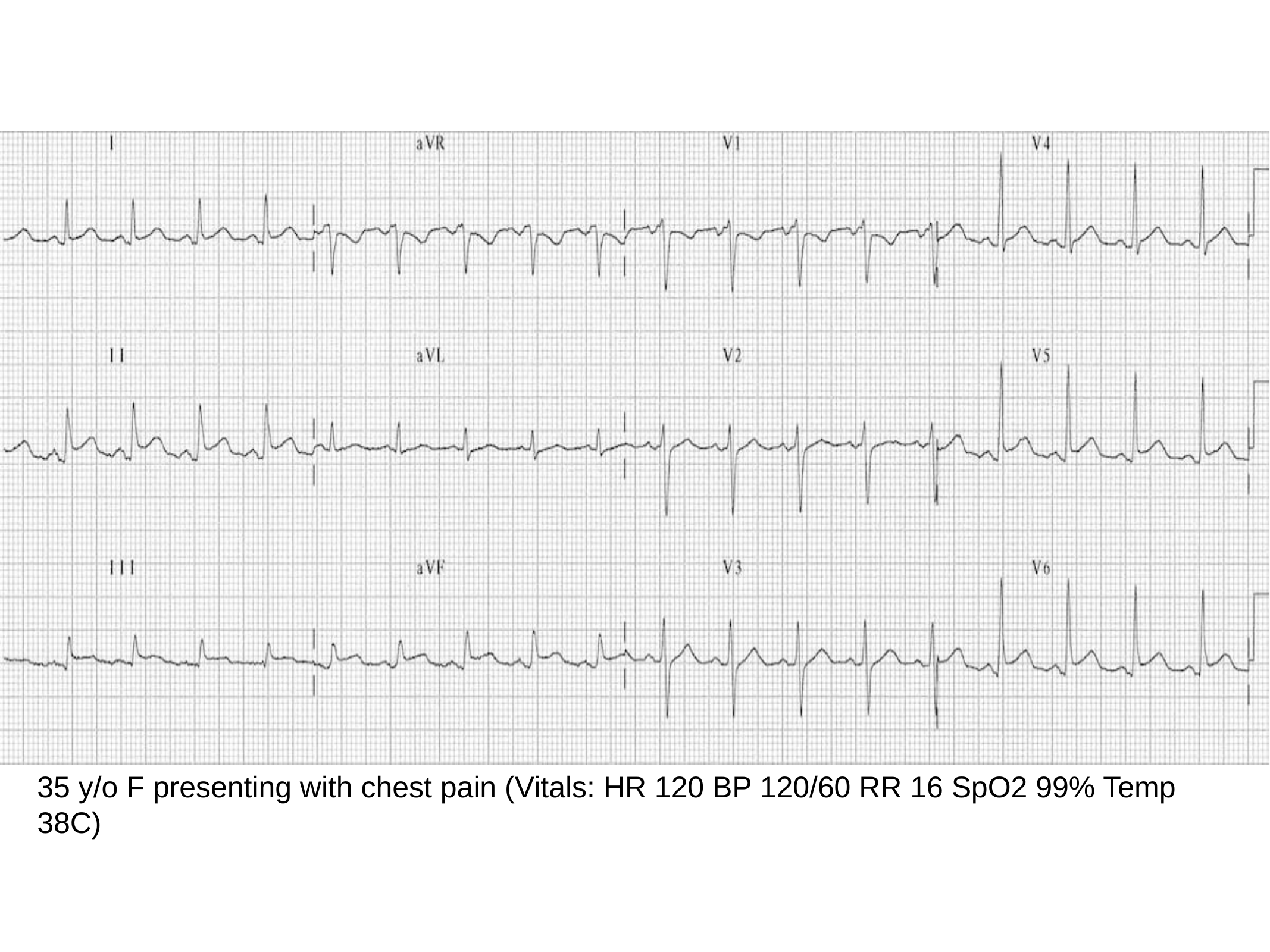

35 y/o F presenting with chest pain (Vitals: HR 120 BP 120/60 RR 16 SpO2 99% Temp 38C)

## Slide 13
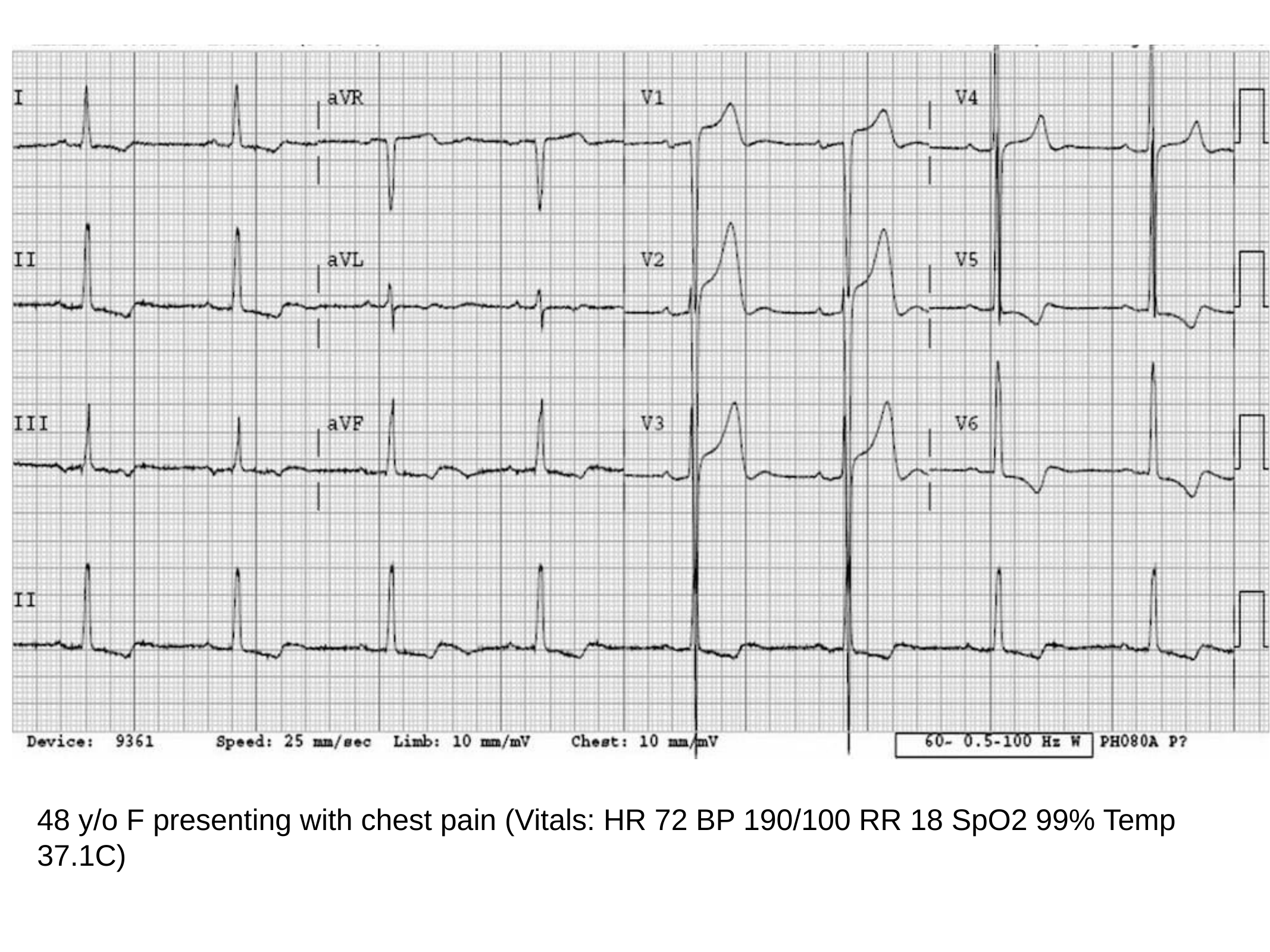

48 y/o F presenting with chest pain (Vitals: HR 72 BP 190/100 RR 18 SpO2 99% Temp 37.1C)

## Slide 14
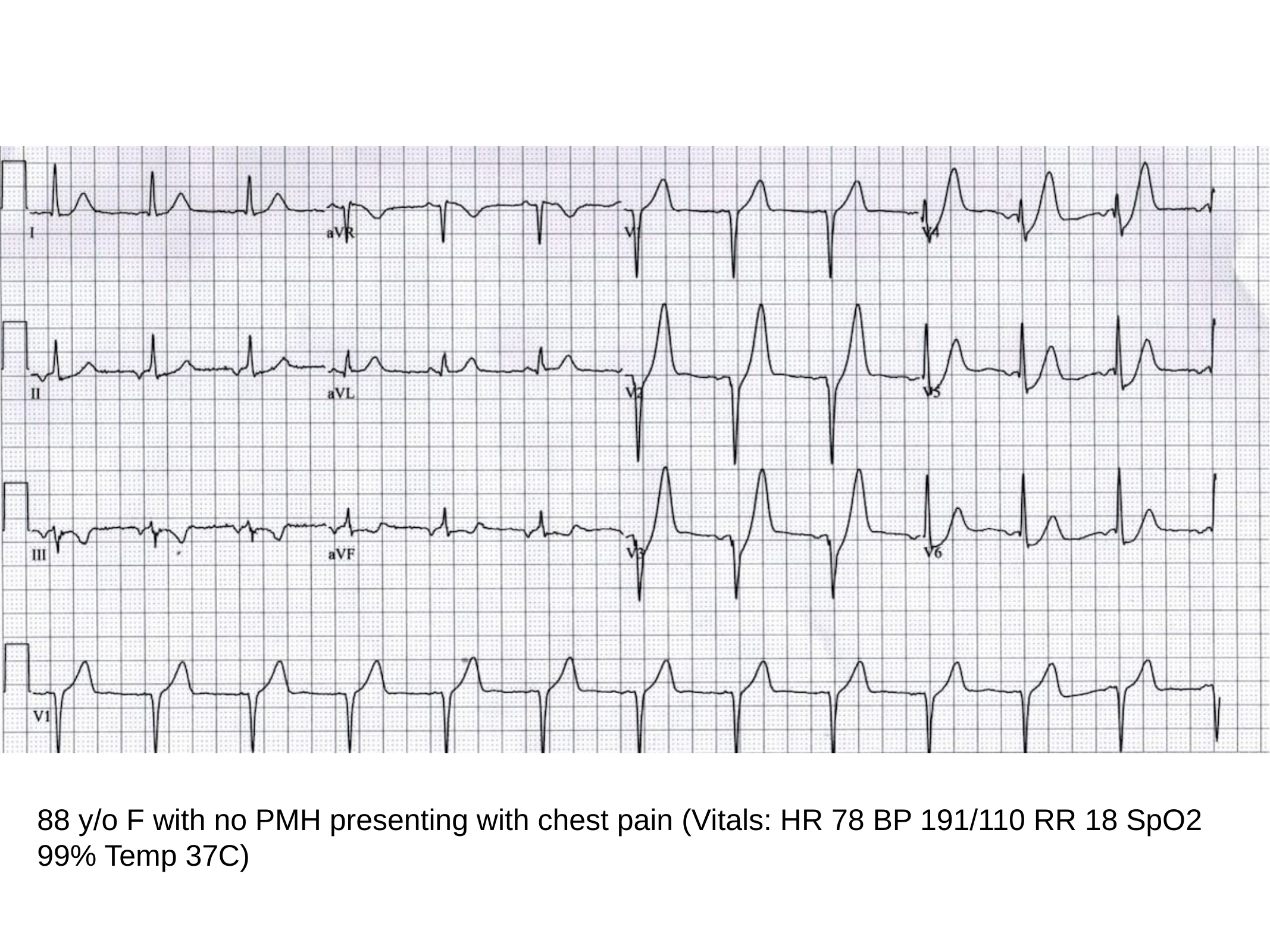

88 y/o F with no PMH presenting with chest pain (Vitals: HR 78 BP 191/110 RR 18 SpO2 99% Temp 37C)

## Slide 15
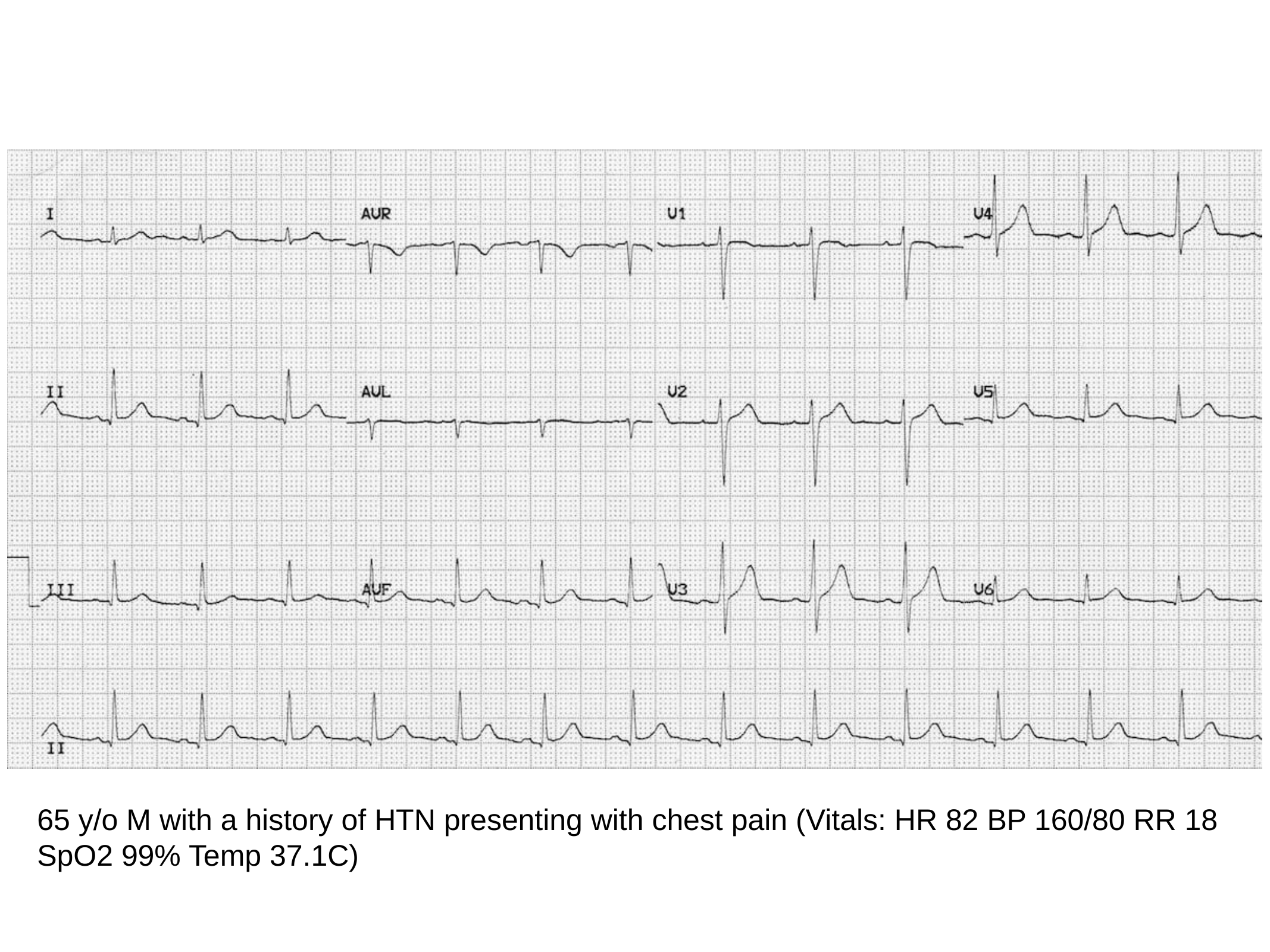

65 y/o M with a history of HTN presenting with chest pain (Vitals: HR 82 BP 160/80 RR 18 SpO2 99% Temp 37.1C)

## Slide 16
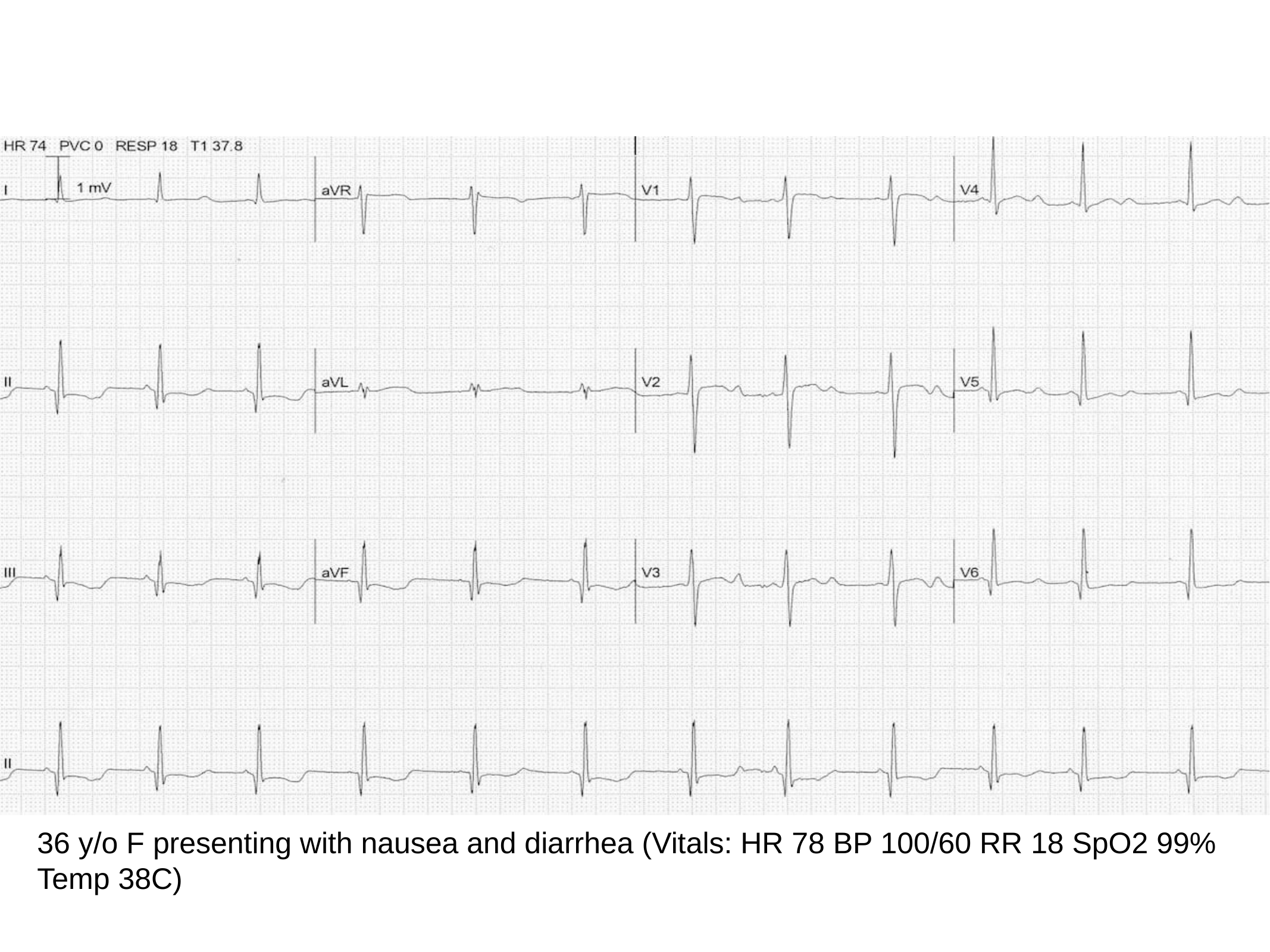

36 y/o F presenting with nausea and diarrhea (Vitals: HR 78 BP 100/60 RR 18 SpO2 99% Temp 38C)

## Slide 17
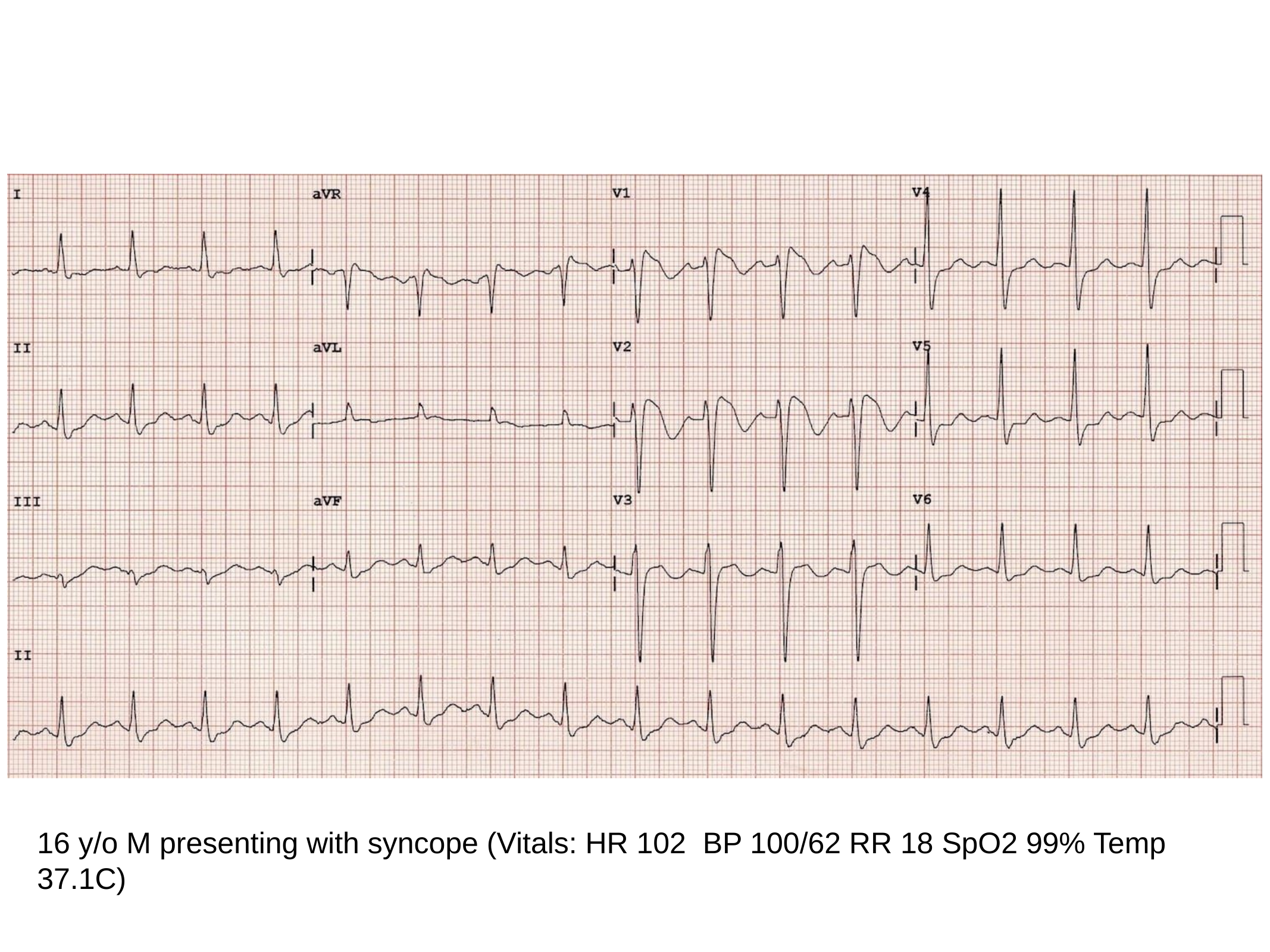

16 y/o M presenting with syncope (Vitals: HR 102 BP 100/62 RR 18 SpO2 99% Temp 37.1C)

## Slide 18
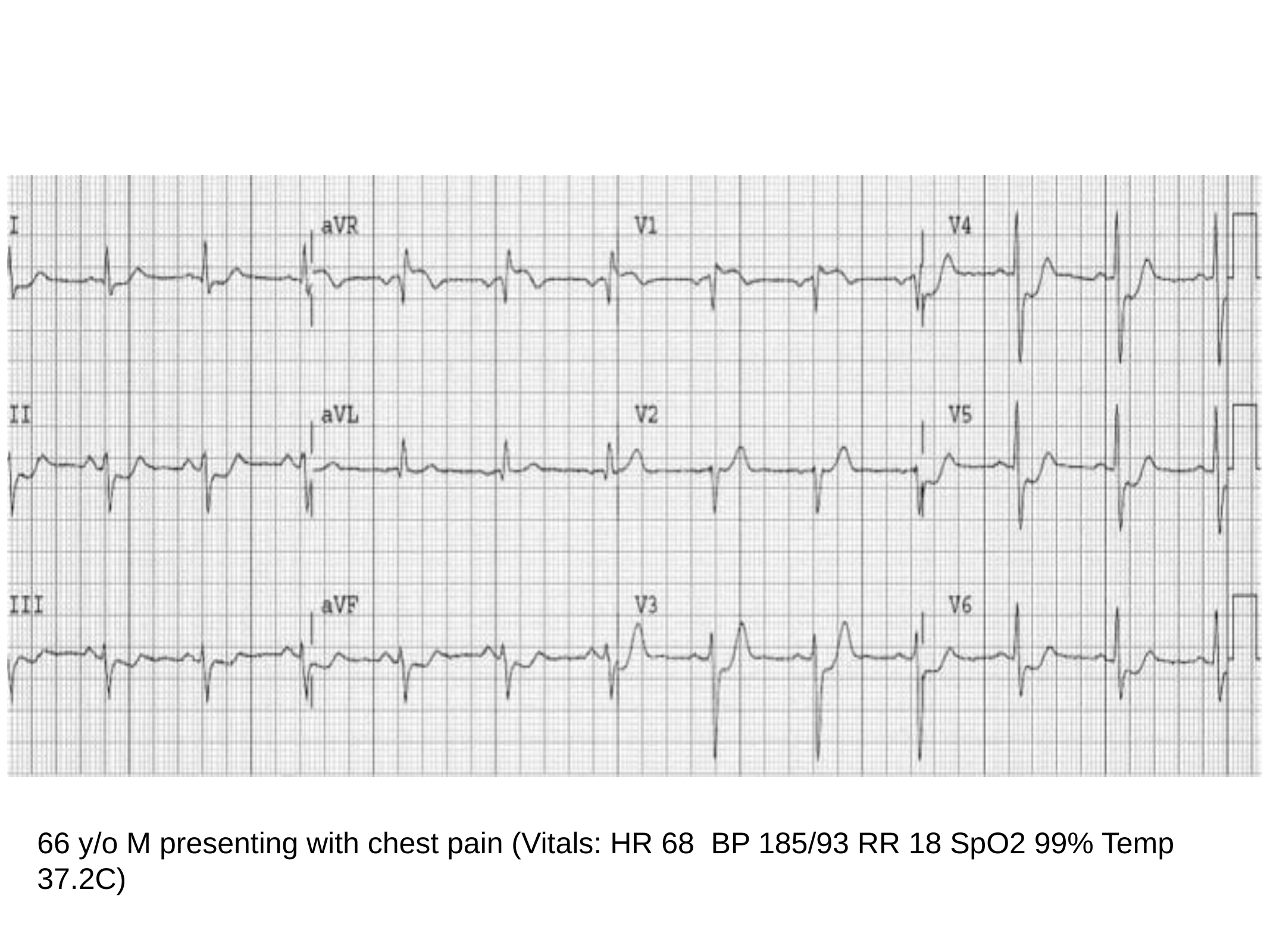

66 y/o M presenting with chest pain (Vitals: HR 68 BP 185/93 RR 18 SpO2 99% Temp 37.2C)

## Slide 19
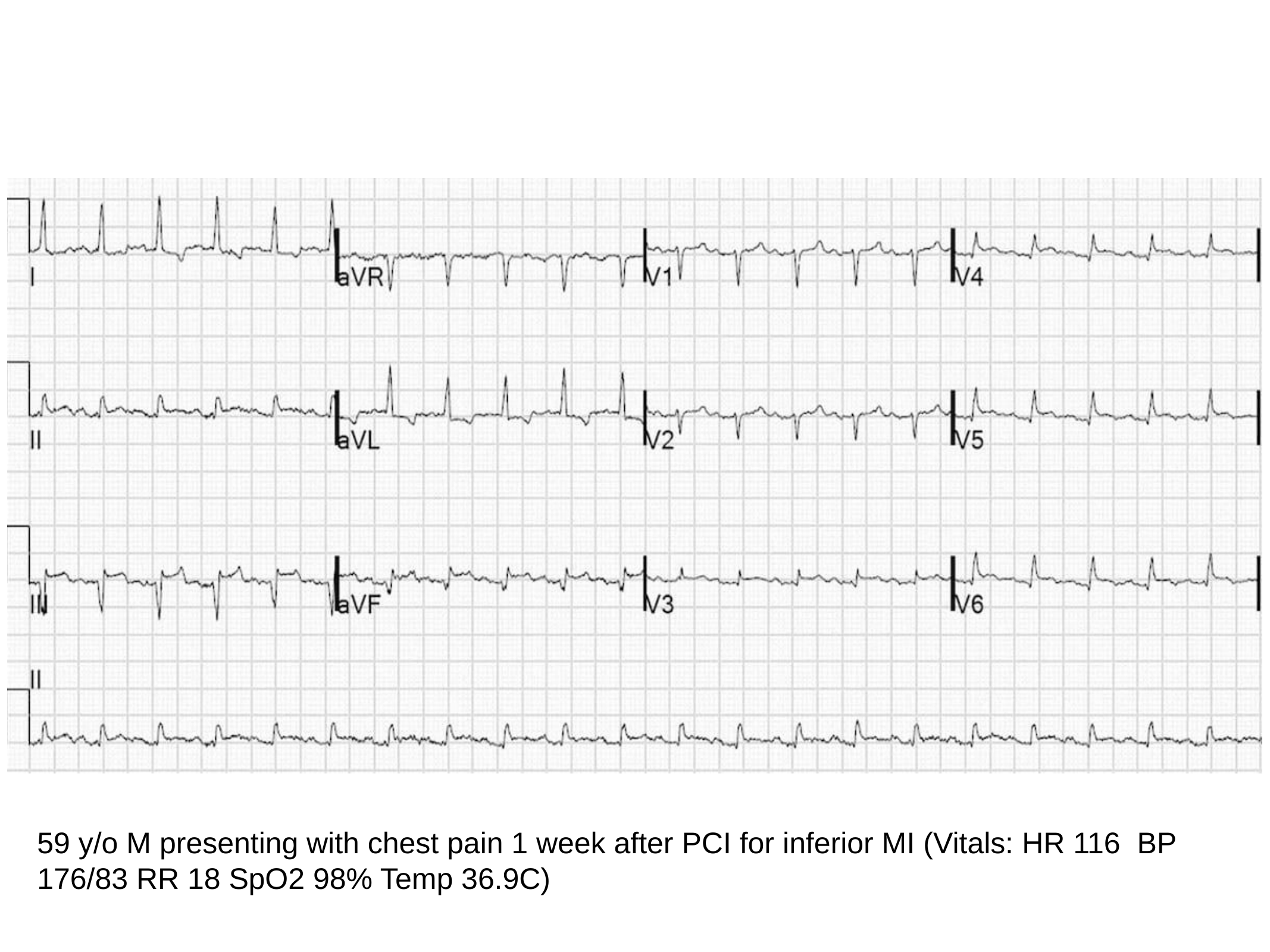

59 y/o M presenting with chest pain 1 week after PCI for inferior MI (Vitals: HR 116 BP 176/83 RR 18 SpO2 98% Temp 36.9C)

## Slide 20
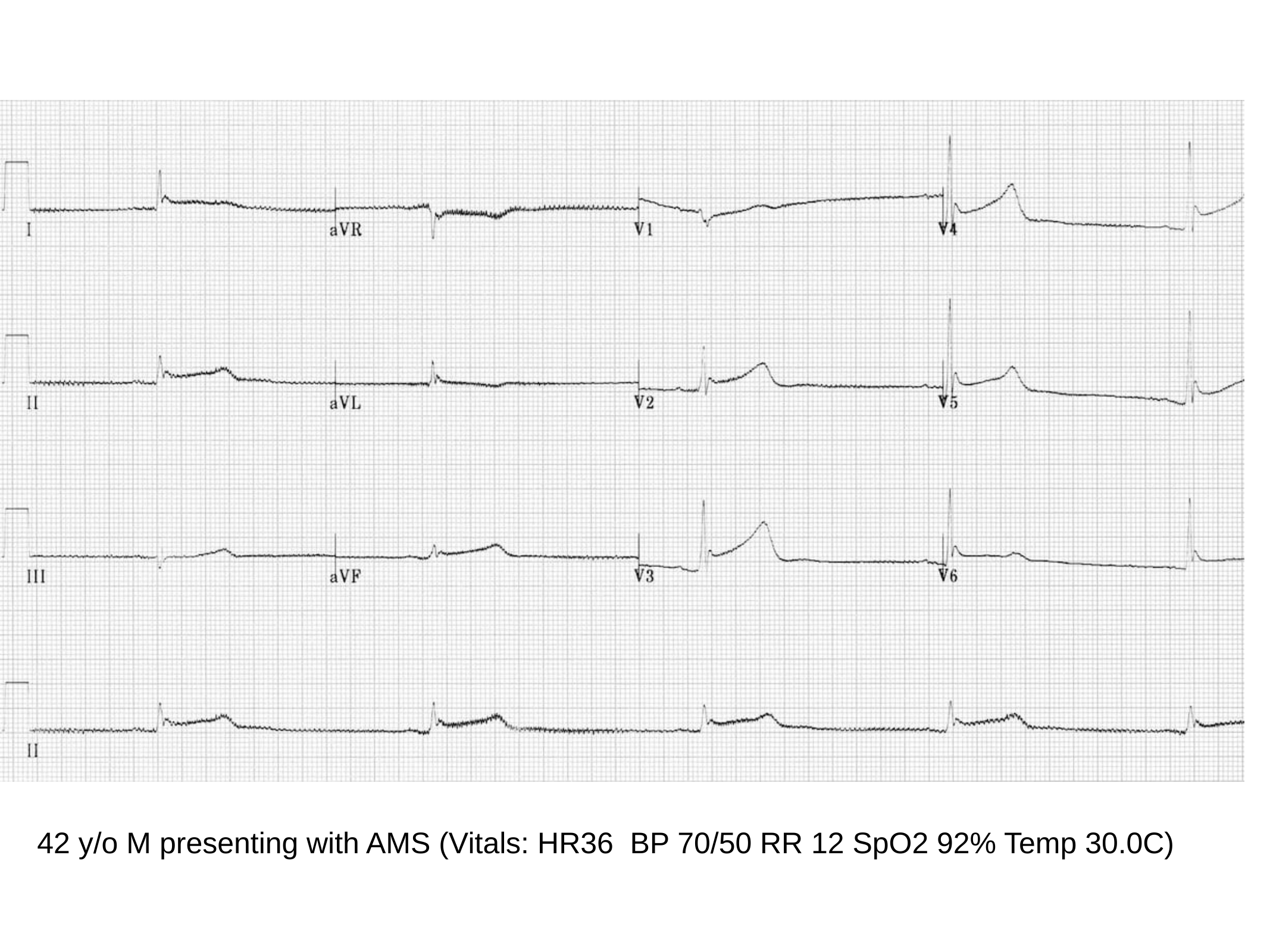

42 y/o M presenting with AMS (Vitals: HR36 BP 70/50 RR 12 SpO2 92% Temp 30.0C)

## Slide 21
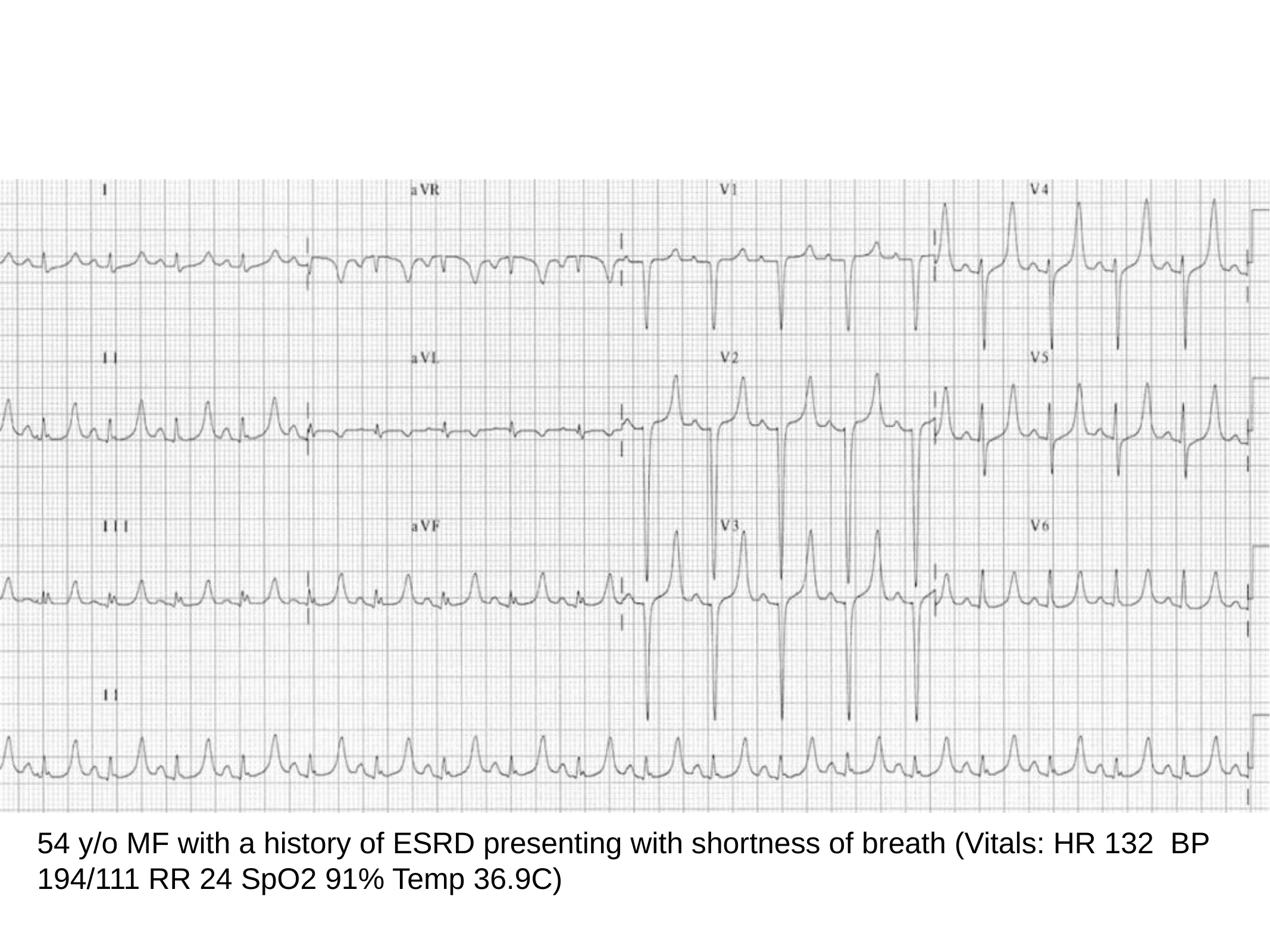

54 y/o MF with a history of ESRD presenting with shortness of breath (Vitals: HR 132 BP 194/111 RR 24 SpO2 91% Temp 36.9C)

## Slide 22
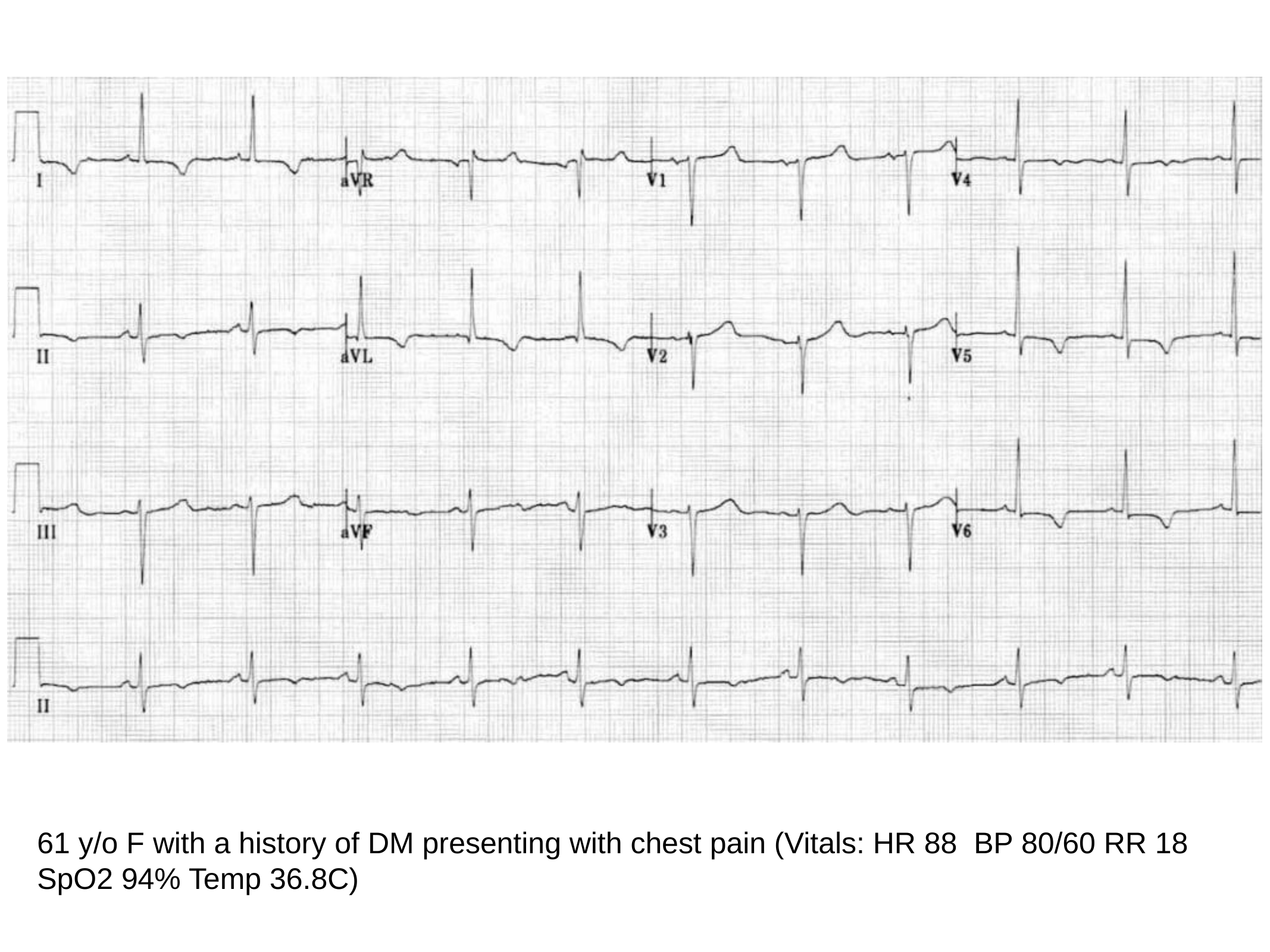

61 y/o F with a history of DM presenting with chest pain (Vitals: HR 88 BP 80/60 RR 18 SpO2 94% Temp 36.8C)

## Slide 23
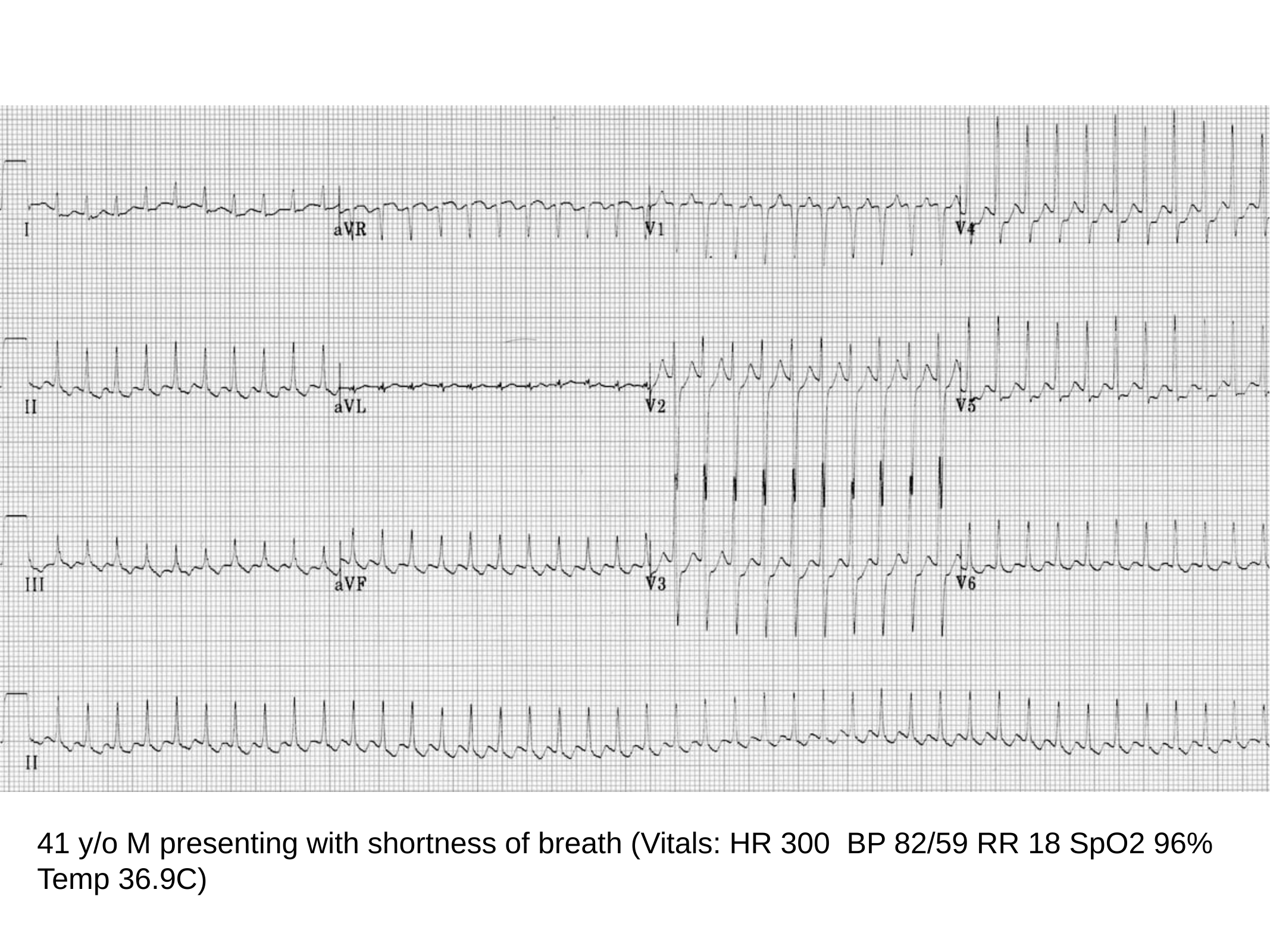

41 y/o M presenting with shortness of breath (Vitals: HR 300 BP 82/59 RR 18 SpO2 96% Temp 36.9C)

## Slide 24
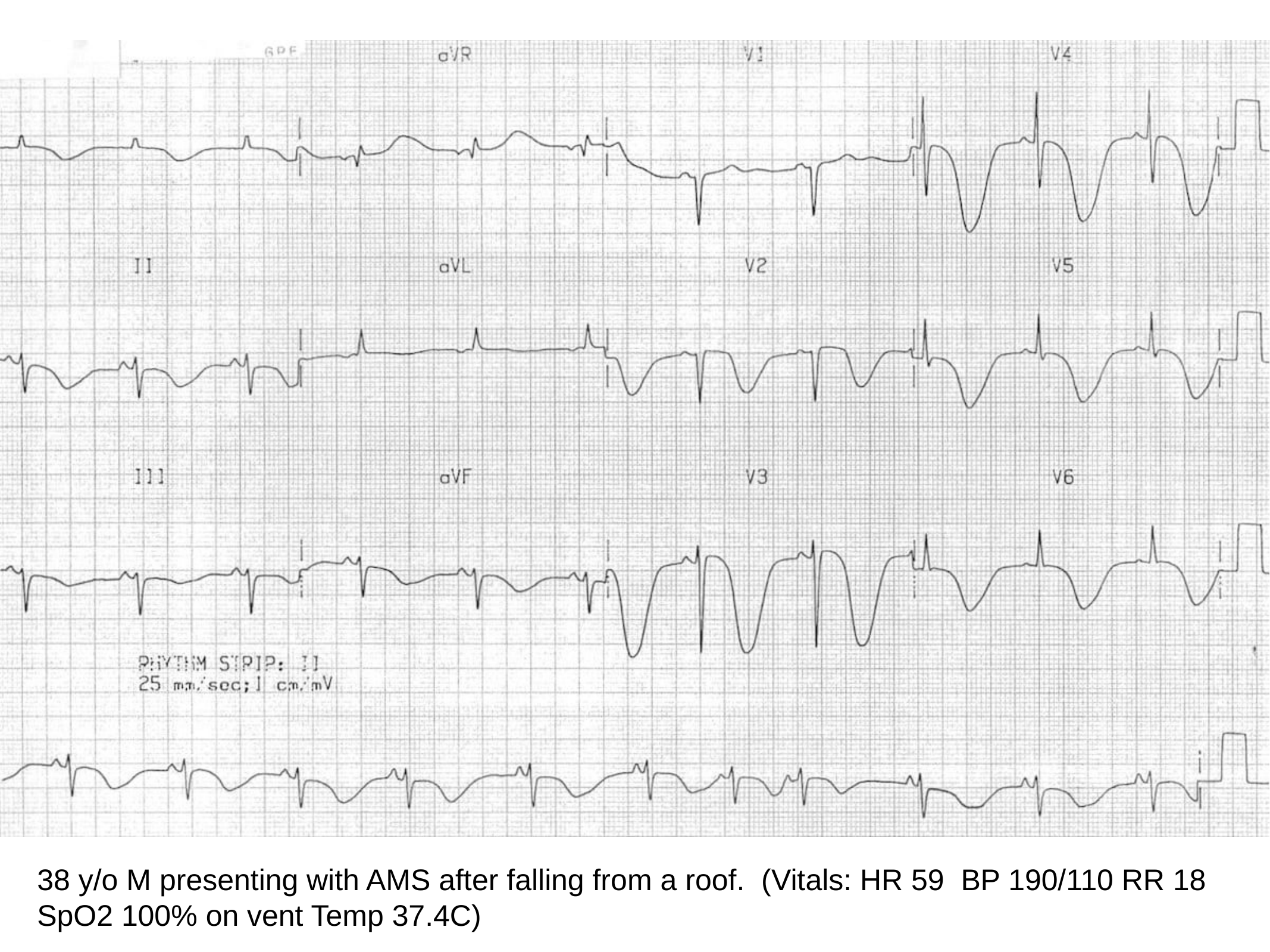

38 y/o M presenting with AMS after falling from a roof. (Vitals: HR 59 BP 190/110 RR 18 SpO2 100% on vent Temp 37.4C)

## Slide 25
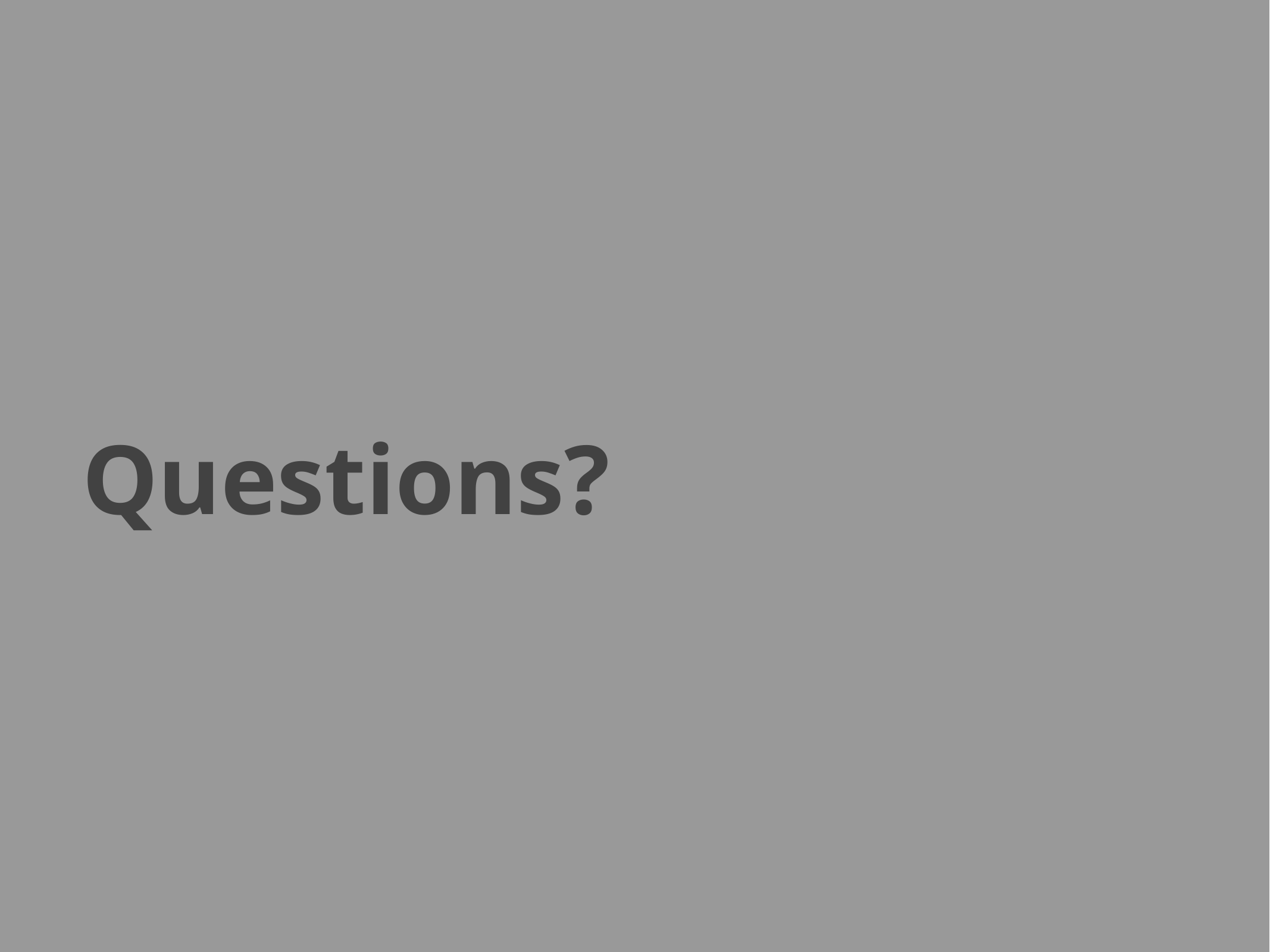

# Questions?
