# Supplementary material for: Ischemic ECG Pattern Recognition to Facilitate Interpretation While Task Switching: A Parallel Curriculum
Source: MedEdPORTAL. 2021 Sep 7;17:11182. doi: 10.15766/mep_2374-8265.11182 (PMC8421424; doi:10.15766/mep_2374-8265.11182)
Supplement: Supplementary file 1 — Introduction Lecture.pptxKnowledge Pretest Answer Sheet.docxECG Handout.docxECG Handout Answers.docxReview Lecture.pptxPresurvey of Confidence.docxPostsurvey of Confidence.docxCourse Evaluation.docxDelayed Knowledge Posttest.docx [file mep_2374-8265.11182-s001.zip › H. Course Evaluation.docx]

Course Evaluation Form

**EM Boot Camp Evaluation**

**Overall assessment of R2 Boot Camp**

1. Overall, how would you rate this experience? (Circle one)

Poor Fair Good Very Good Excellent

2. Please provide your overall thoughts about R2 Boot Camp:

**Didactic Series: Rapid Interpretation of ECGs**

1. Overall, how would you rate this session? (Circle one)

Poor Fair Good Very Good Excellent

2. Please provide any comments about this specific session and suggestions for how it could be improved:
