# Supplementary material for: Ischemic ECG Pattern Recognition to Facilitate Interpretation While Task Switching: A Parallel Curriculum
Source: MedEdPORTAL. 2021 Sep 7;17:11182. doi: 10.15766/mep_2374-8265.11182 (PMC8421424; doi:10.15766/mep_2374-8265.11182)
Supplement: Supplementary file 1 — Introduction Lecture.pptxKnowledge Pretest Answer Sheet.docxECG Handout.docxECG Handout Answers.docxReview Lecture.pptxPresurvey of Confidence.docxPostsurvey of Confidence.docxCourse Evaluation.docxDelayed Knowledge Posttest.docx [file mep_2374-8265.11182-s001.zip › G. Postsurvey of Confidence.docx]

**APPENDIX G: Post-survey**

Thank you for your participation in today’s class. This survey is optional. Your responses will be used to improve teaching for trainees.

In order to link your responses to this survey with future surveys, please provide your initials and the last four digits of your cell phone (i.e., CS-3227)

**Initials:__________________ Last 4 digits of cell phone: _____________________**

**On a scale of 1 to 5, how confident are you in your ability to (1 = not at all confident; 5 = extremely confident):**

| **Identify classic ECGs (e.g. anterior STEMI) that require emergent cardiac catheterization** | **1** | **2** | **3** | **4** | **5** |
| --- | --- | --- | --- | --- | --- |
| **Identify STEMI equivalents on ECGs (e.g. LBBB with Sgarbossa) that require emergent cardiac catheterization** | **1** | **2** | **3** | **4** | **5** |
| **Task switch from another critical task to interpret an ECG** | **1** | **2** | **3** | **4** | **5** |
| **Task switch from another critical task to interpret an ECG without missing an ECG that requires emergent cardiac catheterization** | **1** | **2** | **3** | **4** | **5** |

The survey was created by the author group by adapting a survey used by prior educators when evaluating confidence following an educational intervention.^19^
