# Supplementary material for: Ischemic ECG Pattern Recognition to Facilitate Interpretation While Task Switching: A Parallel Curriculum
Source: MedEdPORTAL. 2021 Sep 7;17:11182. doi: 10.15766/mep_2374-8265.11182 (PMC8421424; doi:10.15766/mep_2374-8265.11182)
Supplement: Supplementary file 1 — Introduction Lecture.pptxKnowledge Pretest Answer Sheet.docxECG Handout.docxECG Handout Answers.docxReview Lecture.pptxPresurvey of Confidence.docxPostsurvey of Confidence.docxCourse Evaluation.docxDelayed Knowledge Posttest.docx [file mep_2374-8265.11182-s001.zip › Educational_Summary_Report_11182.pdf]

MedEdPORTAL® The AAMC Journal of Teaching and Learning Resources

Original Publication Open Access

Ischemic ECG Pattern Recognition to Facilitate Interpretation While Task Switching: A Parallel Curriculum

Caitlin Schrepel, MD*, Ashley E. Amick, MD, MS, Madeline Sayed, Anne K. Chipman, MD, MS

*Corresponding author: caipsch@uw.edu

Abstract

Introduction: Interruptions are an inevitable part of working as an emergency physician, yet these can increase cognitive load and precipitate medical error. Emergency physicians learn to balance these responsibilities using a process called task switching. Yet residents have little exposure to exercises that purposefully integrate task switching during their training. We addressed this gap by exposing emergency medicine (EM) trainees to task-switching events in the form of critical ECG interpretation while they were engaged in concurrent, parallel activities. **Methods:** The curriculum was carried out in three phases. First, 12 PGY 2 residents engaged in a small-group session testing their baseline confidence and ECG interpretation skills. The second phase was longitudinal: During concurrent educational activities, investigators interrupted tasks and asked trainees to interpret ECGs in 10 seconds or less. The curriculum's final phase was used to review the ECGs and answer any questions. **Results:** Confidence and percentage of correct interpretations were compared from phase 1 to phase 2. Participants showed improved confidence ($M = 2.5$, $SD = 0.6$, to $M = 2.9$, $SD = 0.6$; $p = .02$; 5-point Likert scale) and increased mean percent correct ($M = 0.7$, $SD = 0.1$, to $M = 0.8$, $SD = 0.1$; $p = .01$) following the curriculum. **Discussion:** Our curriculum provides a pragmatic, reproducible approach to enhancing critical ECG interpretation with task switching in a way that mirrors the EM practice environment, promoting a reduction of cognitive load and highlighting the skills learners will need as they develop expertise.

Keywords
Task Switching, ECG Interpretation, Cognitive Load, Emergency Medicine, Clinical Training, Procedural Skills Training

Educational Objectives

By the end of this course, learners will be able to:

1. Recognize which ECGs require immediate cardiac catheterization (cath) lab activation in less than 10 seconds.
2. Recognize which ECGs require immediate cath lab activation in less than 10 seconds while task switching from a parallel activity.
3. Report increased confidence in interpretation of ECGs in less than 10 seconds while task switching from a parallel task.

Citation:
Schrepel C, Amick AE, Sayed M, Chipman AK. Ischemic ECG pattern recognition to facilitate interpretation while task switching: a parallel curriculum. *MedEdPORTAL*. 2021;17:11182.
https://doi.org/10.15766/mep_2374-8265.11182

Introduction

Interruptions are a common[1] reality for emergency physicians and can lead to cognitive and medical errors.[2,3] Learning to deal with these interruptions is a necessary skill for emergency medicine (EM) trainees to master since accurately interpreting critical information in a time-sensitive manner while still attending to the current task at hand is crucial to providing patient care. For example, guidelines recommend that any patient who presents to the emergency department (ED) with chest pain must have a screening ECG performed and interpreted by a physician within 10 minutes of arrival to prevent delays in diagnosis of critical ischemia and associated mortality and morbidity.[4,5] As a consequence, physicians in the ED are interrupted multiple times a shift for ECG interpretation. Rather than simply multitasking, these interruptions are managed with a process known as task switching.[6,7] When task switching, the physician shifts attention from their current task to address the interruption. Only when the interrupting task is dealt with do they return their attention to the primary task. The process of task switching is complex and contributes to overall cognitive load. Cognitive load theory[8] suggests that these types of interruptions

Copyright © 2021 Schrepel et al. This is an open-access publication distributed under the terms of the Creative Commons Attribution license.

place increased stress on working memory, increasing the possibility of error. The principles of cognitive load theory and task switching are important concepts for medical educators to understand as they work to find innovative ways to reduce cognitive load. In fact, there is a push for educators to create educational experiences that help learners integrate the skills and knowledge necessary for effective task switching.[9] While this approach to education has been done previously with high-fidelity simulation,[10-12] there is a continued need for pragmatic, easy-to-implement curricular innovations to address this challenge.

Additionally, while previous medical educators have recognized the importance of teaching the skills needed for rapid and accurate interpretation of ECGs, their curricula have focused primarily on teaching ECG basics to novices[13,14] or finding innovative online formats to supplement the core curriculum.[15-17] While one curriculum currently available in *MedEdPORTAL*[16] presents a creative way to use online modules to increase accuracy of ECG interpretation in an emergency setting, the focus is on pediatric emergency patterns rather than the ischemic patterns often seen in adult patients. Additionally, its ECGs are presented as part of online modules, rather than interruptions requiring task switching for interpretation. Our curriculum adds to this body of work by demonstrating that core content, such as ischemic ECG interpretation, can effectively be taught in a manner that mirrors the EM practice environment and promotes skills needed for task switching without requiring extensive resources or high-fidelity simulation.

Methods

All EM residents at our institution participate in a 4-day boot camp prior to their PGY 2 year. This 4-day session involves lectures, small-group discussions, simulation, and procedure lab. All sessions are meant to prepare students for their new role as a senior resident. We implemented our ECG curriculum over the course of this boot camp, but it was designed to be used as a parallel activity to any aspect of the curriculum over any time frame. Our target audience included PGY 2 EM residents as they transitioned to a senior resident role. However, the curriculum could also be used with PGY 1 EM residents or residents in other fields who are required to interpret ECGs while task switching, including residents working in the ICU. The only materials needed were an audiovisual setup, a computer that could run PowerPoint, pen/pencils, and printed ECGs. The study of this curriculum was deemed exempt by the University of Washington Institutional Review Board (STUDY00009976).

All ECGs were obtained from the open-access website Life in the Fast Lane and its ECG library.[18] We selected which ECG patterns to include based on previous core content at our institution, our institutional protocols, and our expertise in ECG interpretation. ECGs were typical representations of the pattern with minimal ambiguity. All authors had to agree on the diagnosis for the ECG to be included. The same set of ECGs was used for all aspects of this curriculum in order to minimize the ambiguity of the diagnosis and maximize our ability to compare pre- and posttests in this study setting.

The curriculum consisted of three components. First, residents engaged in an interactive, 1-hour, small-group session using PowerPoint, which focused on pattern recognition for ECGs requiring emergent cardiac catheterization (cath) and common ST-segment elevation myocardial infarction (STEMI) mimics (Appendix A). During this part of the course, we presented the ECGs on slides and residents had 10 seconds to decide if an ECG required (1) cath lab activation, (2) a call to cardiology for possible cath lab activation, or (3) no activation. They recorded their answer on the provided answer sheet (Appendix B). Of note, the choice of “a call to cardiology for possible cath lab activation” was added as an option given that there were patterns that were not universally part of institutional STEMI protocols (e.g., isolated elevation in aortic valve replacement) in order to allow flexibility when used at other institutions. As each institution has different guidelines on when to activate the cath lab for critical ischemia, these materials may need to be modified in some instances to reflect those differences. Only 10 seconds were given for interpretation as the goal was to emphasize those patterns that need to be distinguished quickly, while understanding that more subtle findings would require more time to interpret. The group then discussed the ECG and the important aspects of the pattern. Residents each had an opportunity to participate in the discussion of the ECG patterns. To maximize participation, we asked all residents to tell the group how they had answered by raising their hand. We then asked for volunteers to explain their reasoning. This interactive gamification approach made use of small-group learning techniques to both engage learners and promote debate and critical reasoning.

The second part of the curriculum was longitudinal in nature. As residents completed the various activities of the boot camp (e.g., lectures, simulation, and procedure lab), we randomly handed them ECGs to interpret (Appendices C and D). For example, a resident might have been participating in a trauma simulation as part of the structured boot camp activities. We interrupted them during their resuscitation of a simulated patient to ask them to

Copyright © 2021 Schrepel et al. This is an open-access publication distributed under the terms of the Creative Commons Attribution license.

interpret an unrelated ECG. These interruptions did not always occur during simultaneous simulations, but also could take place during procedure lab, small groups, and lectures. In each case, the learner had to switch attention away from the primary task to interpret the ECG. When handed the ECG, they had 10 seconds to make the decision regarding cath lab activation. They circled their choice with a pen. All residents received all 20 ECGs in a random and distinct order. This pragmatic simulation was chosen as an instructional technique to promote the skills needed for rapid ECG interpretation while managing other tasks, as is common in the ED. The final component of the curriculum was a 1-hour review session using PowerPoint to discuss the answers for the ECGs handed out during the longitudinal phase. We included notes for the instructors to emphasize important points (Appendix E).

We then evaluated the curriculum with pre- and posttests of confidence (Appendices F and G) and accuracy of ECG interpretation during the pretest (Appendix B) and task-switching activity (Appendix C). The pretest was given prior to any instruction, while the task-switching exercise was used as the posttest to assess whether learners could apply the information they had learned in the classroom while being interrupted. The impact of task switching on accuracy was not directly measured. We created the survey by adapting one used by prior educators when evaluating confidence following an educational intervention.[19] All residents completed our standard course evaluation (Appendix H). Finally, we sent learners a follow-up quiz 1 month later to assess for retention (Appendix I). Data was collected in Microsoft Excel. Mean percent correct and confidence were compared before and after the curriculum using a paired *t* test. A Pearson correlation coefficient was used to explore the relationship between confidence and accuracy on the knowledge assessment.

Results

This curriculum was first implemented in 2018, with three iterations thus far, each with 12 residents. While it has been highly rated on past institutional course evaluations, 2020 was the first year that data regarding accuracy and confidence in interpretation of ECGs were collected. The exercise was completed by all 12 PGY 2 residents in June 2020.

Following the introduction session on pattern recognition, participants demonstrated an increased mean percent correct ($M = 0.7$, $SD = 0.1$, to $M = 0.8$, $SD = 0.1$; $p = .01$) despite the addition of task switching during the posttest. Participants also reported improved overall confidence ($M = 2.5$, $SD = 0.6$, to $M = 2.9$, $SD = 0.6$; $p = .02$; 5-point Likert scale) following the curriculum. Pretest results demonstrated a lack of correlation between confidence and accuracy ($r = .04$, $R^2 = .001$, $p = .92$). Following the curriculum, there was improvement in the correlation between confidence and accuracy, although it was not statistically significant ($r = .53$, $R^2 = .28$, $p = .07$). Unfortunately, the response rate for the 1-month follow-up course was only 16% (two of 12) and therefore could not be used to meaningfully contribute to our results. However, course evaluations for the 2020 class remained extremely positive, with comments from learners such as “This is how all programs should teach ECG” and “This is how we should teach all core content.”

Discussion

Our curriculum is among the first demonstrating that core content can be taught in a manner that mirrors the practice environment, promotes the skills necessary for task switching, and can be easily embedded parallel to existing didactic and simulation curricula, with very little additional resource cost. While we were encouraged by the finding that our learners improved in both accuracy and confidence, this experience also served as an important exercise wherein residents came to understand the unique challenge of performing critical high-stakes tasks while under time and cognitive constraints.

In order to promote the integration of information and reduce cognitive error, it is our role as educators to help learners manage cognitive load while working in the interruption-heavy learning environment of the ED. Our curriculum provides an example of how core content can be taught in a manner that promotes task switching. While not directly measured by our results, the design of our curriculum addresses both intrinsic and extraneous components of cognitive load. The intrinsic load of a task depends on the complexity of the task and the proficiency of the individual.[8] We addressed the intrinsic load of ECG interpretation by harnessing the power of repetition to increase proficiency and by focusing on ischemic pattern recognition to simplify the task and increase automaticity. Extraneous load, on the other hand, which refers to the load imposed on a learner’s working memory that is not necessary for the task at hand,[8] was introduced into the curriculum in the form of distractions. By interrupting residents who were engaged in parallel activities, we were purposefully increasing the extraneous load to emphasize to the learner the skills needed for effective task switching. As trainees develop expertise, they also need to learn the skill of “knowing when to slow down,” as described by Moulton, Regehr, Mylopoulos, and MacRae.[20] In their work, they argue that expertise should be judged by an individual’s ability to respond

Copyright © 2021 Schrepel et al. This is an open-access publication distributed under the terms of the Creative Commons Attribution license.

effectively in the moment using automatic resources and also by their ability to transition to effortful processes when needed. While this ability to switch to effortful processing may take time to develop, our curriculum represents a practical way for educators to begin teaching this crucial skill by forcing trainees to practice task switching. Additionally, as emphasized by researchers such as Ratwani, Fong, Puthumana, and Hettinger,[21] interruption-management skills can be used to prioritize tasks and improve patient safety. As Ratwani and colleagues argue, physicians should be taught the skills needed to effectively resume tasks following an interruption and to delay or avoid interruptions when appropriate. Curricula such as ours could be used to teach learners these techniques while simultaneously emphasizing core content.

In addition to emphasizing the role of cognitive load and task switching on decision-making, our curriculum can provide an opportunity for an important reality check for trainees. While confidence improved following the completion of the sessions overall, it is important to note that confidence was not correlated with accuracy. The lack of correlation was most striking prior to instruction. This may not be surprising to educators who understand that many learners suffer from an illusion of competence.[22] However, it can be surprising to the learners. In fact, several of the learners who struggled the most reached out to leadership for further help after realizing that their confidence did not correlate with their skills. Finally, given the ever-expanding amount of material that is being integrated into resident curricula, it is crucial to continue to explore innovative and practical methods to teach core content. We accomplished this goal in creating a curriculum that requires very few material and personnel resources and can also be modified easily to fit in with existing course structure.

In implementing this course, we learned several lessons. First, we were surprised by the results of the knowledge pretest, which demonstrated a surprising lack of accuracy in recognizing ECGs requiring cath lab activation. This finding suggests that perhaps we need to emphasize this crucial skill more thoroughly during the PGY 1 year. Our learners also experienced some confusion over the option of “a call to cardiology for possible cath lab activation.” Because we think it is important to have this option available given the reality of clinical uncertainty and variability of institutional guidelines in some cases, we will need to spend more time explaining the option to ensure understanding.

While we were encouraged by the improvement of trainee accuracy seen during this course, we must note some important limitations. First, while we intended to use interruptions to mimic the workplace environment and add extraneous load, the simulation of this process was distinct from the interruptions seen in the ED. In the simulated environment, there are fewer competing interruptions, and the stakes linked to the decision are much lower. Additionally, the ECGs selected for this course did not involve ambiguity, and the same set was used for all sessions. These ECGs were selected from Life in the Fast Lane[18] to provide other educators with a starter pack of ECGs; however, this limited our ability to determine how learners would do with more nuanced findings on ECGs. We also do not have any data to determine whether the encouraging results of this curriculum are lasting. We did send out a 1-month follow-up quiz to answer this question, but despite multiple reminders, we did not receive enough completed quizzes to comment on durability of the results. We suspect that our low response rate stemmed from the fact that we utilized an online platform not frequently used by residents for other activities. Additionally, the follow-up quiz was not a required event and had no incentive given for completion. In order to increase the response rate, we would recommend using an in-person format for a follow-up quiz or providing learners with an incentive for completion. Finally, our pre- and postcourse confidence surveys (Appendices F and G) were adapted from a similar survey in the literature, but we did not independently explore its validity in this educational context.

Regardless, we believe that curricula such as this are beneficial as we strive to both teach core content and encourage residents to develop the skills they will need to develop into experts. Future research could include adding more nuanced ECG findings to the course or performing the task-switching task while learners work a real clinical shift. This technique could be expanded to other diagnoses that require a rapid triage evaluation by a provider, such as stroke or arrhythmia. Finally, curricula such as this could be used to teach learners the skills they might need to manage interruptions as they occur.

Appendices

A. Introduction Lecture.pptx
B. Knowledge Pretest Answer Sheet.docx
C. ECG Handout.docx
D. ECG Handout Answers.docx
E. Review Lecture.pptx
F. Presurvey of Confidence.docx

Copyright © 2021 Schrepel et al. This is an open-access publication distributed under the terms of the Creative Commons Attribution license.

G. Postsurvey of Confidence.docx

H. Course Evaluation.docx

I. Delayed Knowledge Posttest.docx

All appendices are peer reviewed as integral parts of the Original Publication.

Caitlin Schrepel, MD: Acting Instructor, Department of Emergency Medicine, University of Washington School of Medicine; ORCID: https://orcid.org/0000-0003-0204-7657

Ashley E. Amick, MD, MS: Acting Instructor, Department of Emergency Medicine and Internal Medicine, University of Washington School of Medicine

Madeline Sayed: Residency Education Manager, Department of Emergency Medicine, University of Washington School of Medicine

Anne K. Chipman, MD, MS: Assistant Professor and Assistant Program Director, Department of Emergency Medicine, University of Washington School of Medicine

Disclosures
None to report.

Funding/Support
None to report.

Ethical Approval
The University of Washington Institutional Review Board deemed further review of this project not necessary.

References

1. Chisholm CD, Collison EK, Nelson DR, Cordell WH. Emergency department workplace interruptions: are emergency physicians "interrupt-driven" and "multitasking"? *Acad Emerg Med*. 2000;7(11):1239-1243. https://doi.org/10.1111/j.1553-2712.2000.tb00469.x

2. Johnson M, Sanchez P, Langdon R, et al. The impact of interruptions on medication errors in hospitals: an observational study of nurses. *J Nurs Manag*. 2017;25(7):498-507. https://doi.org/10.1111/jonm.12486

3. Institute of Medicine. *To Err Is Human: Building a Safer Health System*. National Academies Press; 2000.

4. Pope JH, Aufderheide TP, Ruthazer R, et al. Missed diagnoses of acute cardiac ischemia in the emergency department. *N Engl J Med*. 2000;342(16):1163-1170. https://doi.org/10.1056/NEJM200004203421603

5. 2013 ACCF/AHA guideline for the management of ST-elevation myocardial infarction: executive summary: a report of the American College of Cardiology Foundation/American Heart Association Task Force on Practice Guidelines. *Catheter Cardiovasc Interv*. 2013;82(1):E1-E27. https://doi.org/10.1002/ccd.24776

6. Benda NC, Meadors ML, Hettinger AZ, Ratwani RM. Emergency physician task switching increases with the introduction of a commercial electronic health record. *Ann Emerg Med*. 2016;67(6):741-746. https://doi.org/10.1016/j.annemergmed.2015.07.514

7. Walter SR, Raban MZ, Dunsmuir WTM, Douglas HE, Westbrook JI. Emergency doctors' strategies to manage competing workload demands in an interruptive environment: an observational workflow time study. *Appl Ergon*. 2017;58:454-460. https://doi.org/10.1016/j.apergo.2016.07.020

8. Young JQ, Van Merrienboer J, Durning S, Ten Cate O. Cognitive load theory: implications for medical education: AMEE Guide no. 86. *Med Teach*. 2014;36(5):371-384. https://doi.org/10.3109/0142159X.2014.889290

9. Weidman J, Baker K. The cognitive science of learning: concepts and strategies for the educator and learner. *Anesth Analg*. 2015;121(6):1586-1599. https://doi.org/10.1213/ANE.0000000000000890

10. Soares WE III, Price LL, Prast B, Tarbox E, Mader TJ, Blanchard R. Accuracy screening for ST elevation myocardial infarction in a task-switching simulation. *West J Emerg Med*. 2019;20(1):177-184. https://doi.org/10.5811/westjem.2018.10.39962

11. Turner J, Drew J, Mugele J, Duncan T, Blickendorf R, Cooper D. R2 emergency medicine multipatient simulation. *MedEdPORTAL*. 2015;11:10321. https://doi.org/10.15766/mep_2374-8265.10321

12. Turner J, Wilbur L, Kirschner J, Knopp J, Rodgers K, Cooper D. Third-year emergency medicine multiple-patient simulation. *MedEdPORTAL*. 2014;10:9948. https://doi.org/10.15766/mep_2374-8265.9948

13. Zachow R, Coromilas J, Sadley C. Introduction to electrocardiography diagnostic skills. *MedEdPORTAL*. 2012;8:9159. https://doi.org/10.15766/mep_2374-8265.9159

14. Roberts J. It's all connected: coronary anatomy, EKG interpretation, and acute myocardial infarction. *MedEdPORTAL*. 2015;11:10202. https://doi.org/10.15766/mep_2374-8265.10202

15. Klein AJ, Berlacher M, Doran JA, Corbelli J, Rothenberger SD, Berlacher K. A resident-authored, case-based electrocardiogram email curriculum for internal medicine residents. *MedEdPORTAL*. 2020;16:10927. https://doi.org/10.15766/mep_2374-8265.10927

Copyright © 2021 Schrepel et al. This is an open-access publication distributed under the terms of the Creative Commons Attribution license.

16. Gopwani P, Patel S, Greene EA, Chapman J. A module-based pediatric ECG curriculum to improving accuracy of ECG interpretation by pediatric emergency medicine providers. *MedEdPORTAL*. 2014;10:9779. https://doi.org/10.15766/mep_2374-8265.9779

17. Weinberg J, Ottolini M, Sestokas J, Greene EA. Using a case scenario-based self-teaching module to increase overall skill in ECG interpretation for pediatric residents. *MedEdPORTAL*. 2013;9:9648. https://doi.org/10.15766/mep_2374-8265.9648

18. ECG library. Life in the Fast Lane. Accessed August 22, 2019. https://litfl.com/ecg-library/

19. Triantafylidis LK, Phillips SC, Hawley CE, Schwartz AW. Finding the Sweet Spot: an interactive workshop on diabetes management in older adults. *MedEdPORTAL*. 2019;15:10845. https://doi.org/10.15766/mep_2374-8265.10845

20. Moulton CAE, Regehr G, Mylopoulos M, MacRae HM. Slowing down when you should: a new model of expert judgment. *Acad Med*. 2007;82(10)(suppl):S109-S116. https://doi.org/10.1097/ACM.0b013e3181405a76

21. Ratwani RM, Fong A, Puthumana JS, Hettinger AZ. Emergency physician use of cognitive strategies to manage interruptions. *Ann Emerg Med*. 2017;70(5):683-687. https://doi.org/10.1016/j.annemergmed.2017.04.036

22. Koriat A, Bjork RA. Illusions of competence in monitoring one's knowledge during study. *J Exp Psychol Learn Mem Cogn*. 2005;31(2):187-194. https://doi.org/10.1037/0278-7393.31.2.187

Received: January 13, 2021
Accepted: June 16, 2021
Published: September 7, 2021

Copyright © 2021 Schrepel et al. This is an open-access publication distributed under the terms of the Creative Commons Attribution license.
